# Neuromodulatory effects of audio-proprio-phonatory reinforcement training on subjective tinnitus, a HD-EEG study protocol

**DOI:** 10.1371/journal.pone.0357455

**Published:** 2026-11-06

**Authors:** Isabelle Marié-Bailly, Robin Guillard, Mathilde Wanneveich, Barbara De Dieuleveult, Mahmoud Hassan, Marc Vérin

**Affiliations:** 1 ENT Department, Orléans University Hospital, Loiret, Orléans, France; 2 Mignot Lab, Stanford University, Stanford, California, United States of America; 3 Siopi SAS, Paris, France; 4 Université Paris Cité, Institut Pasteur, AP-HP, INSERM, CNRS, Fondation Pour l'Audition, Institut de l’Audition, IHU reConnect, F-75012, Paris, France; 5 Department of Research and Innovation, Orléans University Hospital, Loiret, Orléans, France; 6 Department of Neurology, Orléans University Hospital, Loiret, Orléans, France; 7 MINDIG Company, Rennes, France; 8 University of Reykjavik, Reykjavik, Iceland; 9 Orléans University Hospital, Loiret, Orléans, France; LSU Health Shreveport, UNITED STATES OF AMERICA

## Abstract

Tinnitus affects 14% of the global population, with 2% experiencing a severe form of the condition. Sound therapy using white noise emitted by hearing aids is a common intervention, supported by the phenomenon of residual inhibition observed during tinnitus assessment. This study aims to investigate whether audio-proprio-phonatory reinforcement training can improve the quality of life of patients with chronic subjective tinnitus by enabling them to listen to their own phonation, which may mask the tinnitus or induce residual inhibition. We will conduct a prospective, controlled, monocentric, 2-arm block-randomized study, stratified by the Tinnitus Handicap Inventory (THI) score, involving two groups for two sequential phases (Phase 1 and Phase 2). Fifty-six patients with tinnitus, recruited from the Ear Nose Throat (ENT) Department of Orléans University Hospital, will undergo a five-week training program, randomized into weekly remote group workshops, and daily individual sessions using specially developed videos. Training efficacy will be assessed through self-report questionnaires. The primary endpoint, the Tinnitus Handicap Inventory, will be assessed at three ENT medical visits: at inclusion, end of phase 1 and end of phase 2. Additionally, patients in both groups will regularly complete Visual Analog Scales for Tinnitus and five related questions (noise, cervical, jaw, sleep, stress-anxiety) via a secure digital platform throughout the study. High-density electroencephalograms (HD-EEG) will be performed in a blinded manner before and after Phase 1 at the Neurology Department of Orléans University Hospital. This pilot study aims to evaluate the clinical efficacy of audio-proprio-phonatory training in patients with chronic subjective tinnitus and to objectively measure its effects using HD-EEG. We will explore potential correlations between changes brain activity (such as functional brain connectivity) and clinical responses to the intervention.

## Introduction

Tinnitus is defined as the subjective perception of sound in the absence of any external auditory stimulus. It arises from dysfunctions affecting the auditory system and related neural structures. This condition can be distressing and may significantly impair an individual’s quality of life [1]. Approximately 14% of the global population is affected by tinnitus, with 2% experiencing a severe form of the condition [2]. Despite its prevalence, effective therapeutic solutions remain limited and fail to address all cases.

Tinnitus can be classified into two categories. Objective tinnitus is associated with internal acoustic stimulation within the body, frequently resulting from vascular abnormalities; this type of tinnitus is relatively rare, affecting only 5% of individuals. In contrast, subjective tinnitus is exclusively perceived by the affected individual and accounts for 95% of all tinnitus cases [3]. It may present acutely with the possibility of spontaneous resolution, or it may develop into a chronic condition. Chronic subjective tinnitus is defined as lasting longer than six months without interruption for a full day. This symptom frequently indicates an underlying health issue, commonly following a peripheral injury, such as sound-induced trauma that damages hair cells in the cochlea [4]. It is also crucial to recognize the connections between auditory pathways and other sensory systems, notably neck and jaw proprioception, a phenomenon referred to as somatosensory tinnitus [5].

The experience of subjective tinnitus is thought to emerge from a complex interplay between peripheral and central auditory dysfunctions. However, the precise mechanisms remain only partially understood [6]. Various models have been proposed. One influential model, the central gain adaptation model (Noreña, 2011) [7], suggests that tinnitus results from increased neuronal activity in the central auditory system following reduced input from the auditory nerve. This imbalance leads to the recruitment of adjacent cortical frequencies, over-amplifying the affected range and generating a phantom auditory perception [5,7]. In 1987, Tonndorf [8] drew an analogy between tinnitus and refractory pain, based on the concept of deafferentation. Later, in 2015, De Ridder [9] proposed an integrative framework combining deafferentation models with a dysfunctional noise cancellation theory, implicating a network of subcortical structures known as the “tinnitus core network.”

Neuroimaging techniques such as high-density electroencephalography (HD-EEG), functional magnetic resonance imaging (fMRI), and functional near-infrared spectroscopy (fNIRS) have revealed that tinnitus onset involves abnormal local neural activity and disrupted functional connectivity within broader brain networks. HD-EEG offers an objective means of assessing symptom severity and monitoring treatment efficacy. Recent studies [10,11] indicate that individuals with chronic tinnitus exhibit increased connectivity between the parahippocampal gyrus, posterior cingulate cortex, and precuneus—regions that form part of the default mode network. These abnormalities are most frequently observed in the right temporal lobe, parietal, and frontal regions. In 2024, Vanneste et al. [12] identified a correlation between heightened alpha activity in the dorsal anterior cingulate cortex and distress, mirroring mechanisms observed in chronic neuropathic pain. They suggest that neuromodulatory treatments should aim to increase alpha activity in the pregenual anterior cingulate cortex while reducing it in the dorsal region.

The neuromodulation process underlying our audio-proprio-phonatory reinforcement training (APPR) protocol may contribute to reducing the distress associated with the perception of phantom sounds, offering a simple and non-invasive therapeutic alternative for individuals with tinnitus.

The audio-phonatory loop is a cognitive attribute that regulates vocal and articulatory output through auditory feedback [13]. This feedback originates from both internal sources (e.g., proprioception, bone conduction) and external sources (e.g., airborne hearing). Proprioceptive feedback plays a crucial role, emphasizing awareness of postures, movements, and vibrations associated with vocal gestures [14].

One widely used intervention for tinnitus alleviation is Tinnitus Retraining Therapy (TRT), typically administered by audiologists [15]. It often includes white noise generators, alone or coupled with hearing aids. Our study proposes a novel cognitive training method - audio-proprio-phonatory reinforcement (APPR) – that integrates principles from phoniatrics and music therapy to address the multifactorial nature of tinnitus. This approach shares features with auditory discrimination training, a form of sound therapy that uses attention redirection via Zen-like sounds [16], which can reduce tinnitus perception and enhance quality of life [17]. Self-vocalization to create a personalized sound environment may facilitate habituation, encourage attentional defocusing, and lessen tinnitus-related psychological distress.

In the fields of Tinnitus and auditory neuroscience, tinnitus masking and residual inhibition (RI) are psychoacoustic constructs based on the use of continuous or pulsed acoustic stimuli. RI reflects the efficacy of masking therapy, especially when the masking sound closely matches the tinnitus frequency [18], highlighting the importance of tinnitus assessment in characterizing symptom severity.

The use of voice to induce residual inhibition remains largely unexplored. However, instances of self-masking have been observed in recently developed therapies [19], which involved producing white noise like the tinnitus percept. It is feasible to manipulate vocal production to align with the spectral characteristics of the tinnitus. Spectrographic analysis can provide visual feedback on voice parameters [20], helping generate sound reinforcements that target tinnitus frequencies and influence perception. Beyond symptomatic relief, using one’s own voice to achieve residual inhibition could improve our understanding of tinnitus pathophysiology and enhance sound-based interventions – such as auditory discrimination training – by reinforcing the audio-proprio-phonatory loop and optimizing its inhibitory potential.

An initial Single-Case Experimental Design (SCED) study was conducted with only three patients. The dual objective was to investigate: (1) whether strengthening the audio-proprio-phonatory loop reduces discomfort from chronic subjective tinnitus, and (2) whether exposure to self-generated sound reinforcements can elicit residual inhibition. The encouraging preliminary findings led to the development of the present clinical study, which aims to explore the impact of a structured training programme designed to improve coordination between auditory, proprioceptive, and phonatory sensory systems on both the perception and management of tinnitus.

### Study objectives and evaluation criteria

The primary objective of this clinical study is to assess the effectiveness of audio-proprio-phonatory reinforcement (APPR) training in reducing tinnitus-related disability in daily life, compared to individuals who have not undergone the training. The Tinnitus Handicap Inventory (THI) – an internationally validated questionnaire – will serve as the primary outcome measure to assess the impact of APPR training on the quality of life of patients with tinnitus.

Secondary objectives include determining whether APPR exercises can provide short-term relief from tinnitus-related annoyance, as measured by the Visual Analog Scale for Tinnitus Annoyance (VAS-A), relative to a control group not receiving APPR training. The study will also examine the long-term maintenance of quality of life (THI scores) and tinnitus-related discomfort following the intervention. In addition, it will monitor the trajectory of patient-reported outcomes over time, including symptoms frequently associated with tinnitus, such as noise sensitivity, cervical pain, jaw complaint, sleep disturbances, and stress-anxiety, comparing participants in the APPR group to those in the control group.

Furthermore, exploratory analyses using HD-EEG (256 channels) will be conducted to investigate the effects of APPR training on neural activity (quantified using several features such as spectral, functional connectivity and dynamic microstate). These analyses will also track longitudinal changes in THI scores and HD-EEG patterns before and after APPR training, in comparison to participants not exposed to the intervention.

## Materials and methods

### Ethics statement

#### Reference methodology.

This study falls within the scope of the ‘Reference Methodology’ (MR-001), pursuant to Deliberation No. 2018−153 of 3 May 2018, which approved a reference methodology for the processing of personal data in the context of health research involving the collection of consent from data subjects and repealed Deliberation No. 2016−262 of 21 July 2016. The study sponsor, Orléans CHU, signed a commitment to comply with this ‘Reference Methodology’ on 10 March 2017.

The study is also subject to the General Data Protection Regulation (GDPR), which came into force on 25 May 2018. An information letter with a consent form gives to the patients the identity of the data controller, the CHU's Data Protection Officer, their right to lodge a complaint with the National Commission on Informatics and Liberty (CNIL), as well as their right of access and rectification, data portability, erasure, and restriction of processing of their personal data.

#### Committee for the protection of individuals.

The protocol, patient information sheet, and all patient-facing documents have been submitted for review to a Committee for the Protection of Individuals selected by random draw. Notification of the Committee's favourable opinion will be sent to both the study sponsor and the competent authority. According to French regulatory authorities, this is a Category 2 study, involving minimal risks and constraints, and requiring the free, informed and explicit consent of all participants. Patients will be fully and clearly informed, in accessible language, of the study’s objectives and constraints, potential risks, necessary monitoring and safety measures, their right to refuse participation, and the possibility of withdrawing consent at any time. All this information is provided in a dedicated information sheet given to the patient. The patient's written consent will be obtained by the investigator, or a physician acting on their behalf, prior to the performance of any study-specific procedures. This protocol has been submitted and approved by a French ethical committee (CPP) under reference 24.05562.00054.

### Study design

This research uses a prospective, controlled, block-randomized, two-sequence, two-period crossover design at a single center. Participants will be randomly allocated evenly to one of two groups: Group A starts with 5 weeks of RAPP training before moving to 5 weeks without training, while Group B begins with 5 weeks without training and then transitions to 5 weeks of RAPP training. Randomization is stratified by THI scores (grade 3 versus grades 4 and 5). Neurology department staff at Orléans University Hospital will conduct HD EEGs, remaining blinded to which treatments participants receive.

This study chose a delayed-intervention control group due to ethical concerns and practical reasons. The main goal is not to show that RAPP training works better than other treatments like standard TRT or sound therapy, but to prove that it is effective and could be a good alternative, especially when there is no hearing loss or only mild deafness that does not require hearing aids.

This crossover design was chosen for ethical reasons to ensure that all participants have access to the RAPP training. However, the duration and persistence of the intervention effects are currently unknown, and a carry-over effect between periods cannot be excluded. Therefore, all outcomes comparing APPR training versus no-training conditions will be based on the first study period only (T0 to T1) between the two group. The full crossover structure will be used to explore the temporal dynamics and persistence of the intervention effects.

A schematic overview of the study design is provided in Table 1.

**Table 1:** Overview of the clinical study design. Fifty-six patients with tinnitus lasting more than three months and a THI score greater than 38 will be randomly assigned at baseline (T0) to either Group A or Group B. Both groups will complete a five-week audio-proprio-phonatory reinforcement training program, delivered in a delayed-start design. THI scores and high-density electroencephalography (HD-EEG) recordings will be collected at T0 and T1 to compare outcomes between Group A (after training) and Group B (prior to training). At T2, THI scores will be reassessed to evaluate the persistence of the intervention effects for group A.

56 patients with tinnitus THI sup 38	*Group A* *28 patients*	Week1 Ex 1a Ex 1b Ex 2a	Week2 Ex 1a Ex 1b Ex 2a	Week3 Ex 2b Ex 3a Ex 3b	Week4 Ex 2b Ex 3a Ex 3b	Week5 Endpoint	EEG-HD THI	Group A: No training					End of Following THI	End of study
Audiometric tests APPR test THI	EEG-HD	**Group A: Audio-Proprio-Phonatory Reinforcement training** VAS-A each Monday and Friday, morning and evening					**Group B: Audio-Proprio-Phonatory Reinforcement training** VAS-A each Monday and Friday, morning and evening				
		VAS-A	VAS-A	VAS-A	VAS-A	VAS-A	VAS-A	VAS-A	VAS-A	VAS-A	VAS-A
	**Group B** **28 patients**	Group B: No training					**Week1** **Ex 1a Ex 1b Ex 2a**	**Week2** **Ex 1a** **Ex 1b** **Ex 2a**	**Week3** **Ex 2b** **Ex 3a** **Ex 3b**	**Week4** **Ex 2b** **Ex 3a** **Ex 3b**	**Week5** **Endpoint**
**T0**		Phase 1					**T1**	Phase 2					**T2**	
	Max 30 days	D1				D35	D42	D49				D85	D92	D99

### Participant timeline: Schedule of enrollment, interventions, and assessments

A schematic overview of the participant timeline, interventions with assessment conducted during medical visits and training periods, is provided in Fig 1.

**Fig 1:**
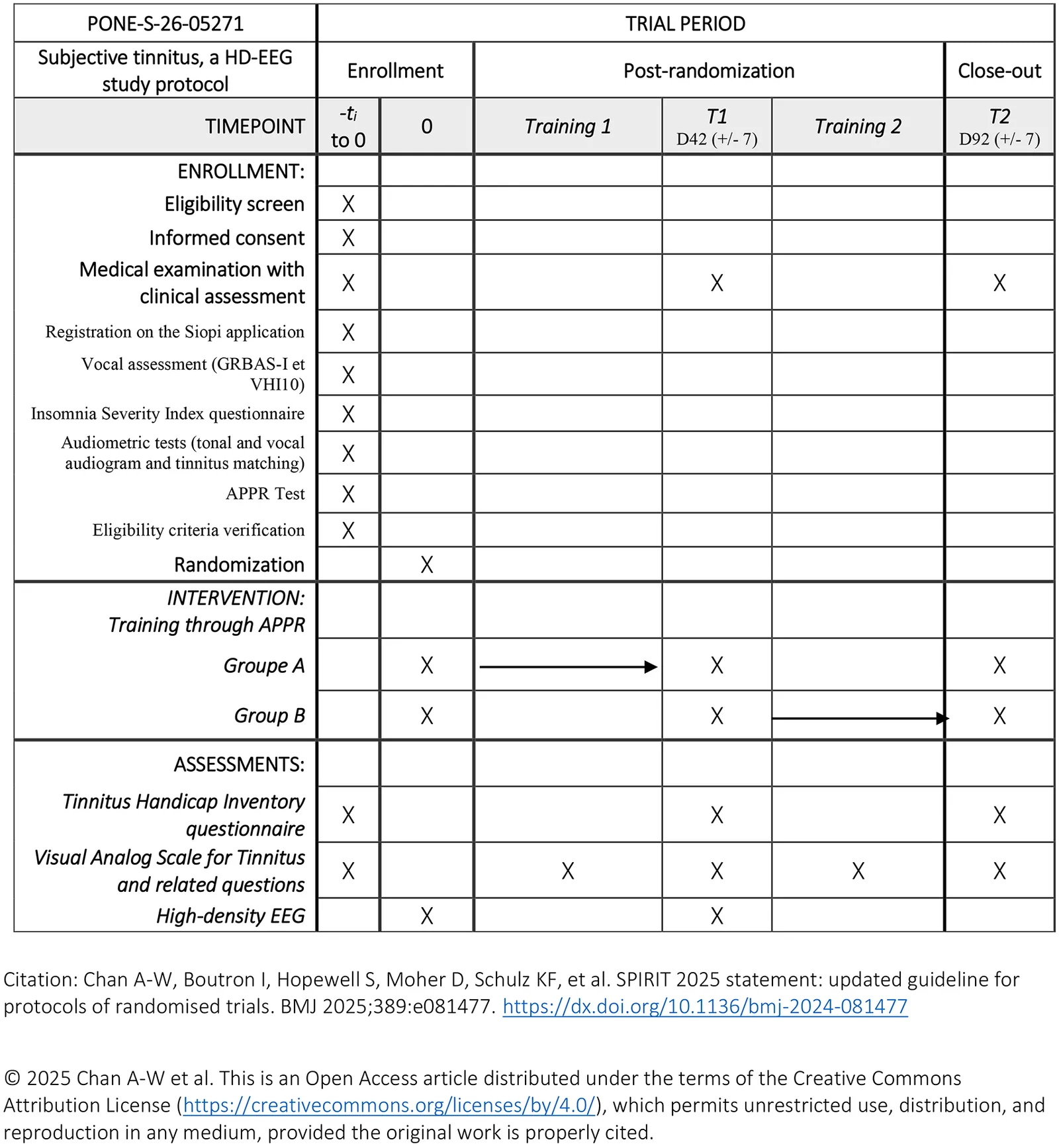
Summary table of assessments conducted during medical visits and training periods. This table outlines the examinations carried out at each stage of the study. Self-assessment questionnaires, administered via the Siopi application, are completed at baseline (T0), after training (T1) and at follow-up (T2), as well as throughout the two training periods. Medical visits at T0, T1, and T2 include APPR tests. At baseline (T0), tinnitus audiometry with residual inhibition (RI) assessment is also conducted. Highdensity *electroencephalography (HD-EGG) recordings are performed at T0 and T1.*

### Participants

Based on data from the literature showing a significant difference of 20 points (SD = 15) between a group receiving transcranial stimulation and a placebo group (Yadollahpour et al., 2024), and on the article by Zeeman et al. (2011), which reports a minimum clinically significant difference on the THI of 7 points (with a similar SD of approximately 14), we hypothesise that five weeks of APPR training will lead to a 12-point difference in THI score at T1 (SD = 14). With a 5% alpha risk and 80% power, and accounting for an estimated 20% of participants with missing THI scores at T0 and T1 (non-analysable), we will include a total of 56 tinnitus patients in the study (28 per arm).

Zeeman et al. (2011) report a minimum clinically important difference (MCID) of 7 for the Tinnitus Handicap Inventory (THI), with a standard deviation of 14. Since no studies have assessed RAPP training's effect on THI, we hypothesize that participants in the 5-week RAPP program will show a 12-point greater reduction in THI scores from T0 to T1 compared to controls—a meaningful difference exceeding the MCID. Using a standard deviation of 14, a 5% significance level, and 80% power, 22 patients per group are needed. With a 20% allowance for non-evaluable cases, the total sample size is set at 56.

#### Recruitment procedures.

Patient recruitment will take place in the ENT department of Orléans University Hospital, primarily by the principal investigator during phoniatric consultations, and through posters. It will be complemented by announcements on the Siopi platform (a French community for sharing information on tinnitus), as well as on the websites of the Orléans Tinnitus Multidisciplinary Team and the MUS’E association (which organizes the group workshops via videoconference), and on social media. Patients will be informed about the study and its objectives through oral explanations provided by the investigator and will receive an information letter. They will have the opportunity to ask any questions directly to the study investigator. If they agree to participate, they will sign a consent form and may then be randomised.

#### Study location.

Patients will attend Orléans University Hospital for medical check-ups and audiometric tests in the ENT department, and for HD-EEG recordings in the neurology department. APPR training will be carried out at home over a period of five consecutive weeks.

#### Study schedule.

The inclusion period will last 36 months. Each participant will be followed for a period of five to six months. The 56 patients to be included in the study will be assigned to different training sessions (each session comprising 6–10 patients), scheduled progressively based on the recruitment and randomisation group. The start of the study is defined as the date the first patient signs the informed consent form. The end of the study corresponds to the date of the last visit by the last patient. The total duration of the study is estimated at 42 months. We estimate that participant recruitment will be completed end of March 2028, that the data collection will be completed end of December 2028 and that we will be able to achieve the expected results end of April 2029.

#### Inclusion criteria.

Participants in this study will be people of indifferent sex between 18 and 85 years, experiencing subjective unilateral or bilateral tinnitus for more than three months, with a THI questionnaire score at the inclusion visit (T0) greater than or equal to 38. They must be able to understand and follow assessment instructions, and they must have access to a computer with an internet connection for the video conference sessions. They must also have a smartphone that is compatible with the Siopi application, on which they will have completed the following questionnaires: THI, VAS-A and associated questions, and the Insomnia Severity Index. Patients will be eligible if they provide informed consent to participate in the study and commit to following the study protocol diligently, including autonomous training, and to refraining from starting any new tinnitus treatments for the duration of the study.

#### Non-inclusion criteria.

Patients will be excluded from the study if they present with objective tinnitus, average bilateral hearing loss exceeding 40 dB on an uncorrected audiogram, or if they report severe to very severe hearing loss on the Siopi questionnaire – regardless of whether they use hearing aids. The use of hearing aids with white noise generators is also an exclusion criterion. Patients diagnosed with psychiatric disorders that cause auditory hallucinations will likewise be excluded. Moreover, participation is not permitted for individuals who have consulted a phoniatric specialist more than twice, attended more than two sound-mediated group workshops, or begun a new therapy less than two months prior to inclusion. Furthermore, the study will not include protected adults (under guardianship or curatorship), individuals under legal protection, persons deprived of liberty, individuals not affiliated with a social security scheme, pregnant or breastfeeding women, or anyone currently enrolled in a drug trial.

While tinnitus is believed to stem from abnormal neuroplastic changes within the central nervous system, its exact cause is still unknown (Zhang et al., 2020). Factors such as hearing loss, hyperacusis, hypertension, or depression further complicate the search for effects specific to tinnitus. These uncontrolled factors may account for inconsistent findings in imaging studies connecting tinnitus with neural irregularities (Vanneste & De Ridder, 2015; Vanneste et al., 2010; Vanneste et al., 2011). A recent clinical trial (Chang-Dong Wang, 2023) excluded participants with tinnitus linked to conditions like Meniere’s disease, pulsatile tinnitus, disorders of the central nervous system, otosclerosis, or a history of middle ear surgery.

Our clinical research investigates how individual and group RAPP training influences psychological, physiological, and neuromodulatory outcomes. Therefore, we do not exclude patients with organic, metabolic, or psychological disorders, except for those experiencing auditory hallucinations, as these may impact EEG-HD data.

During medical recruitment consultation, audiometry with acouphenometry assesses the side, intensity, and frequency of tinnitus, residual inhibition, and somatosensory factors like SADAM, SROS, bruxism, and cervical disorders. The EVA discomfort test tracks changes in tinnitus-related discomfort and five other criteria (hyperacusis, jaw issues, cervical problems, sleep, anxiety) throughout the protocol, highlighting symptoms that may influence RAPP training outcomes.

### Intervention

The experimental phase will begin following randomisation, once enough patients (6–8) have been recruited to form a study group. During Phase 1, patients in group A will undergo APPR training for five weeks. After the first assessment (T1), patients in group B will then train for five weeks between T1 and T2. Regardless of whether they are in a training or non-training period, participants in both groups may continue any current therapies they have been following for at least two months prior to inclusion. During medical visits at T0, T1, and T2, the investigator will document the presence or absence of such ongoing treatments.

#### Audio-proprio-phonatory reinforcement training (APPR).

APPR is a form of cognitive training designed to promote neuroplasticity by strengthening and coordinating connectivity between the auditory, proprioceptive somatosensory, phonatory, and articulatory systems. The training is structured around three categories of exercises in which the patient actively generates multisensory stimuli. Firstly, through silent movements aimed at enhancing proprioception and promoting relaxation of the oro-maxillo-facial region. Then, through self-phonation exercises that encourage defocusing of attention and strengthen auditory and somatosensory discrimination. Finally, through targeted self-phonations at the tinnitus frequency, aiming to elicit residual inhibition. Auditory feedback from these self-phonations is intensified by placing the hands over the ears, either as an amplifying shell (enhancing air conduction) or by occluding the ears (enhancing bone conduction). Additionally, auditory discrimination is supported through visual feedback using a spectrogram provided by Voce Vista software. The training protocol spans five weeks, with daily sessions from Monday to Sunday (seven sessions per week). The exercises are to be carried out every day. Each week begins with a group workshop held via videoconference (on Monday or Tuesday), followed by six independent sessions using pre-recorded videos that reinforce the exercises introduced during the workshop.

#### APPR group workshops.

These remote group workshops, aimed at supporting hearing and communication disorders and originally developed by the MUS’E association during the March 2020 lockdown – have been part of the Orléans Multidisciplinary Tinnitus Team's therapeutic pathway since September 2021. As part of the study, patients will participate in five videoconference workshops via a secure Jitsi link

#### Independent APPR sessions.

The principal investigator has pre-recorded six 30-minute videos. These videos describe in detail each of the six APPR exercises and enable patients to continue their training between group sessions. Each evening at approximately 8 pm, the patient will watch a different video. They will receive the internet link to download this video every day of the week, except the day of the group workshop. During the video, they will perform the suggested exercises, which they will then repeat for around 30 minutes on their own, resulting in approximately one hour of training per day outside of the group sessions. Ten minutes after completing each evening session, patients will record their Tinnitus VAS-A score and their responses to five related questions using the Siopi application. During the next group session, the investigator will assess each patient's engagement by discussing their experiences, and observing their understanding of, and ability to perform, the exercises.

### Commitment of participants

Only patients whose tinnitus significantly impacts their quality of life (THI score greater than 38) will be included in the study, which is expected to enhance compliance with the protocol.

The enjoyable and relaxing nature of APPR training – conducted at home through a combination of group sessions and independent exercises – is also expected to promote participant engagement. Patients will be able to monitor the impact of the training on the perceived tinnitus discomfort, as well as on other associated symptoms (e.g., noise, neck, jaw, sleep, stress-anxiety), by recording their responses twice weekly on the various Visual Analogue Discomfort Scales (VAS) via the Siopi application, downloaded onto their mobile phone, which requires only a few seconds to complete.

We anticipate a high level of patient involvement, as all participants will undergo APPR training, whether assigned to group A (immediate phase 1 intervention from T0 to T1) or group B (delayed intervention phase 2, from T1 to T2).

### Outcome measures throughout the study

Primary endpoint – We will use the international Tinnitus Handicap Inventory (THI) questionnaire to assess the effect of training on the impact of tinnitus on patients’ quality of life. We will compare the change in THI score from T0 to T1 in group A, which receives APPR training, with the change in THI score from T0 to T1 in group B, which does not receive training.

Firstly, we will compare the average daily difference in tinnitus discomfort (evening VAS-A tinnitus score minus morning VAS-A tinnitus score), recorded by patients on the Siopi application four times per week over five weeks, between T0 and T1 for group A (with APPR training) and group B (without training). We will then compare the overall changes over the 5-week period between T0 and T1 for both groups.

Secondary endpoints, to assess the stability of the effects over time, we will use data from group A during the T1-T2 period, in which no training is administered. Results at T2 will be compared to those at T1 for the VAS-A tinnitus scores (measured weekly) and the THI score (measured at T1 and T2).

Additionally, we will compare changes in VAS scores for associated symptoms (noise, neck, jaw, sleep, stress-anxiety), using the daily mean VAS scores over the 5-week period from T0 and T1 for both groups.

Changes in the THI score over time will be evaluated at T0, T1 and T2 for both groups.

These subjective measures will be supplemented by exploratory analyses of the various HD-EEG parameters, recorded at T0 and T1 in both groups. We will compare changes in HD-EEG connectivity and microstate parameters, including network efficiency, shortest network path length, microstate duration, and microstate distribution between the groups with and without APPR training. Finally, we will explore whether changes in these connectivity parameters correlate with changes in quality of life, as measured by the THI score.

### Feasibility

A schematic overview of the different criteria exploring the feasibility of the study, intervention, study design and procedures is provided in Table 2.

**Table 2:** Summary table of the different criteria exploring the feasibility of the study.

	Outcome	Description	Analysis
**Feasibility of intervention**	Safety	Adverse events (mild, moderate, or severe) caused by the intervention	Total number; compare differences in adverse events between the 2 arms
	Compliance/ Adherence	Rate of scheduled and completed sessions	Total n sessions completed /total n sessions scheduled (%) group and individual sessions
	Acceptability	Useability	Conduct qualitative semi-structured interviews to investigate matters related to exercises during group or individual sessions.
	Amount	Training duration in individual sessions	Average duration of training in individual sessions for each patient. Maximum training duration discrepancy in independent sessions among patients.
	Equipment	Function of mobile technology in participants’homes	Number of issues with equipment, such as break downs, shortage of equipment, internet access
**Feasibility of study design** **and procedures**	Acceptability	Encouragement to complete self-assessment surveys	THI and EVA-gene Participation Scale Scores on the Siopi platform. (Compare mean scores between arms)
	Recruitment rates	Number of people approached who consent to take part.	Refusal rates; reasons for non-participation Are the eligibility criteria sufficient or too restrictive
		Collaboration between the ENT department and the neurology department	Number of patients not included due to the inability to schedule an HD-EEG appointment.
	Equipment	Accessibility of high-density EEG measures at the neurology department of the University Hospital Center of Orléans.	The number of EEG-HD headsets available from the beginning to the end of the protocol. The setup time required for each EEG-HD measurement.
	Participation rates	Do all qualified participants who consent to participate engage in the training intervention?	Total number who undergo baseline assessment compared to total number who complete final testing.
	Assessment time scale	Can follow up data be collected within 2 weeks of completing the 5 weeks of training?	Number of participants whose follow-up data were collected within 2 weeks after the 5-week training period.

### Standardisation

Upon completion of the clinical study, our six daily training videos will be released as open source to facilitate standardisation of RAPP exercises through progressive stages. The initial stage consists of proprioception exercises (1a, 1b), followed by sound exercises (2a, 2b), and concluding with residual inhibition exercises (3a, 3b).

Despite careful planning, standardising five consecutive weekly group sound mediation workshops is challenging. Variations in group dynamics, stemming from the therapists’ and patients’ personalities, attitudes, and engagement, significantly affect each session, regardless of hearing impairment.

### Attendance

Adherence to both individual and group training is monitored post-completion using two approaches: patients fill out a follow-up sheet during their consultation and they also complete an online questionnaire. The questionnaire records the number of times training videos were viewed, the exercises practiced from six instructional videos, experiences gained during five group sessions, and main insights from each session's video.

Regarding the strategy for handling missing data, participants will receive verbal reminders during study visits and interactions only. More intensive reminders were deliberately avoided, as repeatedly prompting participants to assess their tinnitus may increase their focus on it and interfere with the habituation process (which is a key objective of the intervention).

### Data collection and management

Most of the data required by the protocol will be recorded in an electronic case report form (eCRF) created by the Orléans CHU using Ennov Clinical software. Only authorized personnel (investigators, clinical research staff, etc.) with a personal and secure password (in compliance with CNIL MR001) will be permitted to enter data in the eCRF. The accuracy of the data will be verified through automatic consistency checks, and a clinical research associate will ensure compliance with data collection standards. Data will be backed up daily. During the study or at its conclusion, all data collected on participants and transmitted to the sponsor by the investigators (or other qualified personnel) will be pseudonymised, with only the patient's identification code displayed.

Data generated during HD-EEG recordings or on SIOPI application will be pseudonymised directly by the recording system, stored securely at Orléans University Hospital, and made accessible only to authorised personnel for further analysis.

#### Statistical analyses.

The analysis will use a modified intention-to-treat group, which includes all participants who were randomly assigned and completed the THI score both at the start (T0) and at the end (T1) of the intervention period, following the study protocol.

Quantitative variables will be compared between groups (A and B) using Student’s t-test, provided its validity conditions are satisfied. If these conditions are not met, the Student’s t-test for unequal variances or the nonparametric Wilcoxon/Mann-Whitney test will be employed. To adjust for potential confounding variables, a linear regression model will be applied, with verification of all relevant model assumptions.

Longitudinal data will be evaluated through mixed-effects models that include fixed effects and a random intercept for each participant.

Within-group comparisons will utilize paired Student’s t-tests, or the signed Wilcoxon test if the t-test assumptions are violated. Pearson’s correlation coefficient will be used to assess correlations.

The primary outcome will serve as the sole confirmatory hypothesis test. Analyses involving secondary outcomes are considered exploratory. When multiple comparisons arise within a family of secondary outcomes (such as VAS scales), p-values will be adjusted using the Holm-Bonferroni method. For mixed-effects models, applicable post hoc pairwise comparisons will be adjusted for multiple testing according to procedures implemented in the statistical software.

The EVA scales are filled out four times each week. During RAPP training weeks, evaluations take place on group workshop days (Monday or Tuesday) both in the morning and evening, as well as on Friday. In control weeks, when there is no RAPP training, assessments are done every Monday and Friday, both in the morning and evening.

For the analysis of the first secondary outcome, two calculation methods are employed. The first method calculates the five-week average of daily differences on each VAS-G scale for each group: this is derived by summing the differences between the morning and evening VAS-G measurements across five weeks, then dividing by the total number of measurements (5 weeks × 2 measurements per week). The second method determines the daily average of each VAS for each group by averaging the morning and evening VAS-G scores: (morning VAS-G + evening VAS-G) / 2.

The primary outcome is THI. Group differences in THI scores at T1 will be compared using a specified statistical test, with regression to adjust for confounders like background treatment or RAAP score. Box plots will show group THI scores.

#### SIOPI application data management.

Data on the SIOPI platform is managed and stored by the system of record. Patient information will be pseudonymized using a study-specific page, where each participant enters their patient number in PHONACOU. Study data is stored on SIOPI, and at the study's conclusion, only coded data will be delivered in person to the biostatistician at Orléans University Hospital.

#### Data management and quality control.

Prospective data will be gathered through an electronic data entry system (eCRF, Siopi). To make sure the information is consistent and reliable, a quality control plan has been established. This includes automatic validation checks when entering data (such as acceptable value ranges, required fields, and duplicate detection), independent review by a designated data manager, as well as regular monitorings during recruitment. Any errors or missing data will be examined, documented, and tracked. The information will be kept on a secure server that meets current regulations (like GDPR) and only authorized staff will have access. A comprehensive Data Management Plan (DMP) can be provided if needed.

#### HD-EEG data management.

HD-EEG data will be analysed using both spectral and functional connectivity analysis [21]. This includes data pre-processing, feature extraction, statistical analysis and result evaluation. Correlations between changes in EEG features and the THI score (between T0 and T1) will be explored using Pearson or Spearman correlation tests.

HD-EEG data will be stored and transferred—using only patient codes specific to the study—to MINDIG via a secure platform, which includes a password-protected account. All data will remain on a secure MINDIG server throughout the period of statistical analysis. Once this analysis is complete, both raw and processed data will be securely sent to the sponsor, deleted from the MINDIG server, and archived at Orléans University Hospital on its secure server. Participants will be notified about whether their consent was specifically obtained regarding possible future reuse of their data for other research purposes, pending the necessary approvals from authorities and the participant. This information will also be documented in the study database.

HD-EEG data will be analysed primarily using MNE-Python, an open-source Python toolbox for electrophysiological data analysis, together with standard scientific Python libraries, including NumPy, SciPy, Pandas, scikit-learn, and Network X. The preprocessing pipeline will include data import, channel inspection, band-pass filtering, notch filtering, bad-channel detection and interpolation, re-referencing, segmentation of resting-state recordings, and artefact correction. Ocular, muscular, and cardiac artefacts will be identified using a combination of automated procedures, visual inspection, and independent component analysis implemented in MNE-Python. For each participant, quality-control metrics will be documented, including the number of rejected and interpolated channels, rejected epochs, and retained clean data duration.

Following preprocessing, cortical source activity will be reconstructed using MNE-Python inverse modelling tools. In the absence of individual MRI data, source reconstruction will be performed using a standard anatomical template adapted to the 256-channel HD-EEG montage. Source-level time series will be extracted from anatomically defined regions of interest, with particular focus on tinnitus-related networks, including auditory cortices, temporal and parietal regions, posterior cingulate cortex, precuneus, parahippocampal regions, and anterior cingulate cortex.

The primary HD-EEG outcome will be the change from T0 to T1 in source-level resting-state functional connectivity within tinnitus-related networks, comparing the immediate-training group with the delayed-training control group. Connectivity will be estimated in canonical frequency bands, with particular emphasis on the alpha band. Secondary exploratory EEG outcomes will include spectral power changes and dynamic microstate parameters, including microstate duration, occurrence, coverage, and transition probabilities. Associations between EEG changes and clinical improvement will be explored by correlating EEG-derived changes with changes in Tinnitus Handicap Inventory scores.

### Benefit / risk

This is a Category 2 human research study that does not involve any invasive procedures. Throughout the study, participants are not exposed to any major risks. The audiometric tests are standard ENT procedures, as are the use of the spectrogram, commonly employed in phoniatric consultations or speech therapy sessions. The audio-proprio-phonatory training represents only a minimal constraint, whether carried out in group workshops or independently.

No specific risks or physical constraints are anticipated in connection with the HD-EEG examination. This technique is non-invasive and poses no danger, apart from the possibility of mild skin irritation (as noted in the equipment manufacturer's documentation: EGI©), which may rarely occur due to electrode contact. Participants may experience fatigue during the various sessions; this will be monitored, and breaks will be offered if needed. At the end of the experiment, participants may notice some stickiness in their hair caused by the saline solution, which can easily be washed out with clean water. Electrodes may leave minor marks, which typically disappear quickly.

The direct benefit lies in the potential improvement in participants’ quality of life – not only through the effect of APPR exercises on their tinnitus perception, but also through the positive experience of being heard and understood in a supportive group setting, and through gaining a sense of autonomy as active contributors to their own health.

### Dissemination

We will ensure that the results of this trial are published in peer-reviewed publications and presented at various scientific conferences to ENT specialists, phoniatricians and speech and language therapists. Our aim is to inform and educate clinicians, thereby influencing their practice to improve patient care. Upon reasonable request, we will share and collaborate with other research teams by providing access to the study data, particularly with members of the Association Francophone des Équipes Pluridisciplinaires en Acouphénologie (AFREPA).

## Discussion

This prospective, controlled, single-centre study aimed to evaluate the neuromodulatory effects of a five-week Audio-Proprio-Phonatory Reinforcement (APPR) sound therapy training on the quality of life of patients suffering from disabling subjective tinnitus for over three months.

In addition to sound gain, hearing aids offer today various sound therapies based on the psychoacoustic characteristics of tinnitus, with heterogeneous levels of evidence in the literature. Some, such as Sequential Sound Therapy (SST), modulate masking based on intensity. Others, like Combined Tinnitus Therapy (CT) or Tinnitus Retraining Therapy (TRT), combine sound stimulation with psycho-emotional support to address both the affective and perceptual dimension of tinnitus [5,22,23]. These approaches provide a range of therapeutic options, enabling personalised care tailored to each patient’s needs.

Our protocol is based on an innovative “tinnitus” sound therapy methodology – a psycho-somato-sensory approach combining phoniatrics and music therapy. While it can be like TRT, this approach is ecological, playful and interactive. Here, the patient becomes the active generator of multisensory stimuli during autonomous individual sessions, complemented by remote group sessions. This study focuses on the impact of APPR training on tinnitus patients’ quality of life – with or without hearing aids, but explicitly excluding TRT (an exclusion criterion) – to avoid bias between therapies. Nevertheless, a future clinical trial could usefully compare APPR-based sound therapy with that embedded in hearing aids.

In the context of tinnitus, reducing discomfort involves alleviating the distress, stress, anxiety and other negative symptoms associated with its daily perception. Assessing the impact of tinnitus on patients’ lives is therefore essential for identifying the most appropriate therapeutic strategy. The Tinnitus Research Initiative (TRI) recommends evaluating tinnitus impact through specific key parameters [5]. A global understanding of tinnitus requires an approach that integrates both subjective and objective assessment tools [24]. Digital technologies now provide new means of evaluating therapeutic treatment efficacy. For instance, we will use the mobile application Siopi [25] to recruit participants and monitor their symptoms via the questionnaires described below.

In this trial, we aim to demonstrate the positive impact of APPR training on tinnitus patients’ quality of life, based on stratified randomisation into two groups (A and B – deferred) according to their scores on the self-report questionnaire (THI score, the primary subjective outcome), completed at the time of their inclusion (T0). By comparing short-term changes in THI scores between the two groups after the training period (T1), we expect to observe an improvement in the quality of life of patients in group A, who will have undergone APPR training. This improvement would be reflected in a greater reduction in THI scores compared to group B, who will not yet have received the training. The THI assessments will be complemented by VAS-A scales to provide a more detailed understanding of the training’s impact.

All self-assessments will be completed by patients using the Siopi mobile application, which will allow them to track changes in their VAS scores. We anticipate that this regular monitoring will foster greater motivation and adherence to the protocol. All data from patient self-assessments (THI and VAS-A) will be securely stored on the Siopi platform.

To support and objectify these self-reported outcomes, we will conduct exploratory HD-EEG analyses at T0 (inclusion) and T1 (after the training phase in group A). These analyses will examine the effects of APPR training on the brain activity by comparing the EEG features (spectral, connectivity, dynamic microstates...) between the two groups. The EEG results will be analysed in parallel with changes in patients’ quality of life, as assessed by THI scores over time. We will specifically investigate several brain regions of interest and compare our findings with those from recent studies [10,26]. Our primary hypothesis is that patients with chronic tinnitus will exhibit increased connectivity between the parahippocampal gyrus, the posterior cingulate cortex, and the precuneus – key nodes of the default mode network – with additional involvement of the right temporal, parietal and frontal regions.

We also aim to confirm findings from a recent study by Vanneste [12], which demonstrated that increased alpha activity in the dorsal anterior cingulate cortex is correlated with distress, via a mechanism like that observed in chronic neuropathic pain. This study suggests that neuromodulatory treatments for tinnitus may be effective by promoting alpha activity in the anterior part of the pregenual cingulate cortex and reducing it in the dorsal anterior cingulate cortex.

Through our exploratory HD-EEG analyses at T0 and T1, we hope to detect similar mechanisms in the pregenual cingulate cortex of group A patients after training. If observed in parallel with improved quality of life (as evidenced by reduced THI scores), such findings would support the hypothesis that APPR training exerts a neuromodulatory effect on tinnitus.

### Limitations

Several limitations of this study must be acknowledged. One is the absence of blinding for the principal investigator, particularly regarding the HD-EEG assessments. Secondly, the inclusion and exclusion criteria – favouring patients with tinnitus lasting more than three months and with a THI score of at least 38 – may introduce some heterogeneity in the study population. Nevertheless, several assessments will be conducted during the inclusion visit (T0), including the Insomnia Severity Index, which will allow evaluation of patients’ insomnia level over the past few months. This will complement the data collected through the sleep disturbance VAS throughout the 5-week protocol.

Other assessments, such as the GRABS-I and VHI scales, are commonly used in phoniatrics to evaluate laryngeal functions. However, in this study, we will use a specific APPR test with visual retrocontrol via spectrogram, based on exercises introduced during training. The aim of this test is to familiarise patients with this original approach and to assess, together with them, their audio-proprio-phonatory discrimination ability, the frequency of their tinnitus, and the possible presence of residual inhibition (RI) through self-perception of their vocal emissions. This could help explain the varying effects of APPR training across different individuals.

## Conclusion

This clinical study aims to evaluate the neuromodulatory effect of audio-proprio-phonatory reinforcement training as part of the therapeutic management of patients suffering from chronic, disabling tinnitus. Specifically, it assesses its impacts on improving their quality of life and fostering patient engagement in self-management. This innovative intervention enables patients to consciously act on the disturbing auditory stimulus and modulate their tinnitus perception – often intensified by stress – thereby reducing feelings of helplessness. In addition, the group-based workshop format promotes a sense of autonomy and personal responsibility for health, while offering the psychological benefits of being heard and understood within a supportive environment.

Finally, through this clinical study, we share our insights into the neuromodulatory effects of audio-proprio-phonatory reinforcement training with other healthcare professionals, to support its integration into routine clinical practice. Given its primarily audio-phonatory nature, this method is particularly suited to speech and language therapists, who are well-positioned to implement and evaluate it in clinical settings. To support this, a continuing education programme will be launched, enabling professionals (ENT specialists, general practitioners, hearing care professionals, etc.) to remotely participate in APPR workshops.

This initiative aims to foster further clinical research and help validate this new therapeutic strategy as an ecological alternative to hearing aid-based sound therapy – particularly for patients with subnormal hearing.

## Supporting information

S1 File PHONACOU revised protocol. (PDF)

S2 File SPIRIT 2025 expanded checklist. (PDF)
