# Supplementary material for: Neuromodulatory effects of audio-proprio-phonatory reinforcement training on subjective tinnitus, a HD-EEG study protocol
Source: PLoS One. 2026 Nov 6;21(11):e0357455. doi: 10.1371/journal.pone.0357455 (PMC13742906; doi:10.1371/journal.pone.0357455)
Supplement: Supplementary file 1 [file pone.0357455.s001.pdf]

Clinical study involving humans with minimal risks and constraints (category 2 of L1121-1)

PHONACOU

Neuromodulatory effects of audio-proprio-phonatory reinforcement training on subjective tinnitus, objectified by HD EEG

Version n°1.2 dated 10/07/2026

**SPONSOR**		
**Orléans University Hospital Research and Innovation Department**<br>14 avenue de l'hôpital<br>CS 86709,45067 Orléans Cedex 02		☎ 02 38 74 47 79<br>🖷 02 38 74 43 64<br>juliette.vilcot-crepy@chu-orleans.fr
**PRINCIPAL INVESTIGATOR**		
**Dr. Isabelle Marié-Bailly ENT Department**<br>14 avenue de l'hôpital<br>CS 86709,45067 Orléans Cedex 02		☎ 02 38 74 43 28<br>isabelle.marie-bailly@chu-orleans.fr
**CLINICAL TRIAL PROTOCOL**		
**Study Code**		CHUO-2024-18
**N° IDRCB**		2024-A02506-41
**N° Clinical trial**		NCT06880367
**PERMISSIONS**		
**COMMITTEE FOR THE PROTECTION OF PERSONS**		
**CPP**	**File Number:** 2024-A02506-41	**Date of the initial favourable opinion:** 21/02/2025
**COMPETENT AUTHORITY**		
**ANSM**	**Date of transmission for information:**	
**NATIONAL COMMISSION ON INFORMATION TECHNOLOGY AND FREEDOM**		
**Declaration of Conformity to the Reference Methodology (MR-001):** 10 March 2017		

This protocole is licensed under a Creative Commons Attribution 4.0 International License (CC BY 4.0). To view a copy of this licence, visit https://creativecommons.org/licenses/by/4.0/.

TABLE OF CONTENTS

Protocol Agreement....4
Tracking protocol changes....5
Summary....6
1. General information....11
1.1. Project Leaders....11
1.2. Scientific Committee....11
1.3. Oversight Committee....11
1.4. Other Committees....11
2. Background and scientific rationale....12
2.1. Description of the pathology....12
2.2. Rationale and Description of the Strategy Studied....14
2.3. Summary of results from available non-clinical trials and clinical trials....16
3. Research objectives....20
3.1. Main objective....20
3.2. Primary outcome....20
3.3. Secondary Objectives....20
3.4. Secondary outcomes....20
4. Study population....22
4.1. Population Description....22
4.2. Inclusion criteria....22
4.3. Criteria for non-inclusion....22
4.4. Recruitment procedures....23
4.5. Benefit/risk....23
5. Research Design....24
5.1. Research Methodology....24
5.2. Specific procedures for research....24
5.3. Conduct of the study....32
5.4. Duration of the study....35
5.5. Description of permanent or temporary shutdown rules....35
6. Safety assessment....36
7. Right of access to data and source document....36
7.1. Access to data....36

7.2. Documents sources 36
7.3. Data to be collected directly in the case report form and which will be considered source data 37
7.4. Case Report Book 37
7.5. Data Entry and Generation 37
7.6. Data Privacy 37
8. Statistiques...39
8.1. Population et stratégie d'analyses 39
8.2. Planned statistical methods 40
8.3. Anticipated number of individuals to be included in the research....................................43
8.4. Statistical criteria for stopping the search 43
9. Quality Control and Assurance...44
9.1. Access to data 44
10. Administrative and regulatory aspects..44
10.1. Initial notice 44
10.2. Substantial changes...44
10.3. Patient information and express consent 45
10.4. Indemnification of subjects 45
10.5. Registration in the national register of persons suitable for biomedical research.............45
10.6. CNIL 45
10.7. Archiving..45
11. Financing and insurance 46
11.1. Study Budget...46
11.2. Insurance 46
12. Feasibility of the study 47
13. Publication rules...47
14. Bibliography 48
15. List of Appendices 56

PROTOCOL AGREEMENT

SIGNATURE OF THE PRINCIPAL INVESTIGATOR

I have read all the pages of the protocol of the clinical trial of which the ORLEANS University Hospital is the sponsor. I confirm that it contains all the information necessary to conduct the test. I undertake to carry out the trial in accordance with the protocol and the terms and conditions defined therein. I undertake to carry out the test in accordance with:

- The principles of the "Declaration of Helsinki",
- International and French rules and recommendations of good clinical practice (rules of good clinical practice for biomedical research on medicinal products for human use)
- National legislation and regulations relating to clinical trials,
- Compliance with the EU Clinical Trials Directive [2001/20/EC].

I am also committed to ensuring that investigators and other qualified members of my team have access to copies of this protocol and documents related to the conduct of the trial to enable them to work in accordance with the provisions contained in these documents.

NAME: Isabelle MARIÉ-BAILLY

Signature : Date :18/02/2025

In accordance with the provisions of the law relating to information technology, files and freedoms, you have the right to access and rectify computerized data concerning you at any time (law no. 2004-801 of 6 August 2004 amending law no. 78-17 of 6 January 1978 relating to information technology, files and freedoms).

SIGNATURE OF THE SPONSOR

Sponsor :

NAME : Juliette VILCOT CREPY
Directrice de la recherche et de l'innovation
Signature :
Date : ____________________

TRACKING PROTOCOL CHANGES

List of Substantial Changes

Numéro et Objet de la MS	Récapitulatif des modifications apportées
**MS01**<br>•	•
**MS02**<br>•	•

List of Substantial Changes

Numéro et Objet de la MNS	Récapitulatif des modifications apportées
**MNS01**<br>•	•
**MNS02**<br>•	•

SUMMARY

TITLE	**Neuromodulatory effects of audio-proprio-phonatory reinforcement training on subjective tinnitus, objectified by HD EEG**
ACRONYM	**PHONACOU**
KEYWORDS	Subjective tinnitus, neuromodulation, HD EEG, audio-proprio-phonatory reinforcement
SPONSOR	**Centre Hospitalier Universitaire d'Orléans**<br>Direction de la Recherche et de l'Innovation<br>14 avenue de l'hôpital<br>45067 Orléans Cedex 02
PRINCIPAL INVESTIGATOR	**Dr Isabelle Marié-Bailly**<br>Centre Hospitalier Universitaire d'Orléans<br>Service ORL<br>14 avenue de l'hôpital<br>CS 86709,45067 Orléans Cedex 02
PROTOCOL VERSION	V1.1 du 17/02/2025
RATIONALE /BACKGROUND	Tinnitus affects 10% of people in France, 1% of whom suffer from it, and therapeutic solutions are still struggling to respond satisfactorily to all situations. Sound therapy by emitting white noise in hearing aids, in addition to hearing gain, is one of the pillars of the support for tinnitus sufferers to improve their quality of life evaluated by the THI.<br><br>Tinnitus is a unified phenomenological coherent perception, linking several separable clinical characteristics, such as its volume, laterality, type (pure tone, noise), associated distress. A theoretical pathophysiological framework capable of explaining all these aspects in a single model was proposed by De Ridder in 2015 integrating the neurophysiological models based on deafferentation and the dysfunctional noise suppression model and proposing a subnetwork of "tinnitus core".<br><br>In research in acouphenology and auditory cognitive neuroscience, tinnitus masking and residual inhibition (IR) are two psychoacoustic measures made from acoustic sequences composed of pulsed or continuous stimuli. IR is manifested by the temporary reduction or suppression of tinnitus after prolonged acoustic stimulation (Fournier et al., 2018; Hu et al., 2021) and predicts the success of masking therapy, which is more effective with noise emitted close to tinnitus frequencies (Fournier et al., 2018).<br><br>Studies in high-density electroencephalography (HD-EEG) (Lan et al., 2021) or fNIFRS (Hu et al., 2024) have demonstrated that the appearance of tinnitus results from a dynamic process resulting in aberrant local neuronal activity and abnormal connectivity in multifunctional brain networks. The HD EEG provides objective measures of the severity of symptoms and thus makes it possible to quantify the effectiveness of a treatment.<br><br>A study in sound therapy (Huang et al., 2021) demonstrated that notched sound significantly reduces the connectivity of Brodmann's area 46 (BA46) involved in the

	inhibition of negative affect and improves tinnitus by regulating the auditory and emotional cortex.<br><br>Finally, Vanneste in 2024, based on his very recent observations in EEG-HD, comparing the mechanism of tinnitus to that of chronic neuropathic pain, suggests the interest of potential tinnitus treatments by promoting alpha activity in the anterior part of the pregenual cingulate cortex and the reduction of alpha activity in the dorsal anterior cingulate cortex by pharmacological or neuromodulatory approaches.<br><br>Our ecological approach to neuromodulation in "tinnitus" offers tinnitus patients to be their own transmitter of sound stimuli by audio-proprio-phonatory reinforcement. (RAPP) Through auto-phonations, they discover the infinite possibilities of sound production, (Borel & Leybaert, 2020), which can mask their tinnitus or produce residual inhibition, without the need for external input.<br><br>Through this clinical study, we wish to demonstrate the beneficial effect of audio-proprio-phonatory reinforcement (APR) training in chronic subjective tinnitus sufferers. This, completed and objectified by EEG HD, by researching whether there is a link between the evolution of connectivity in the above-mentioned brain areas and the favourable response to the therapeutic protocol.
MAIN OBJECTIVE	To determine whether audio-proprio-phonatory reinforcement (APR) training reduces tinnitus-related disability in daily life compared to patients who have not received RAPP training.
SECONDARY OBJECTIVES	1- To determine whether RAPP exercises reduce tinnitus-related discomfort in the short term (EVA Tinnitus Discomfort Scale), compared to patients who have not undergone RAPP training<br>2- To study the stability over time of quality of life (THI score) and tinnitus-related discomfort (VAS scale) after RAPP training.<br>3- Describe the change in patients' quality of life (THI score) over time<br>4- To describe the evolution of other tinnitus collateral discomforts (noise, cervical, jaw, sleep, stress-anxiety) in patients who have undergone RAPP training, compared to patients without RAPP training.<br><br>**Exploratory analyses in HD-EEG will also be carried out to:**<br>○ To assess the effect of RAPP training on the usual connectivity and microstate parameters of HD-EEG, compared to patients who have not undergone RAPP training<br>○ To describe the evolution of patients' quality of life (THI score) before and after RAPP training and of the usual parameters of connectivity and HD EEG micro-state, over time.
PRIMARY OUTCOME	**Tinnitus Handicap Inventory (THI) score** measured at T0 (baseline) for all patients in the study and then at T1, i.e. after a 5-week RAPP training period for group A; or after a 5-week non-training period for group B.
SECONDARY OUTCOMES	**1- Visual Analog Scale** for Tinnitus (VAS-G) completed weekly (4 times a week) between T0 and T1 in **the 2 groups (with and without RAPP training).**

	**2-** **EVA** tinnitus discomfort scales completed 4 times a week between T1 and T2, and the THI score measured at T1 and T2, for **group A (without RAPP training over this period).**<br>**3-** **THI score** measured at T0, T1 and T2 (T2 being the last visit, approximately 5 weeks after T1) for **the 2 groups**.<br>**4-** Other **VAS annoyance** scales (noise, cervical, jaw, sleep, stress-anxiety), completed weekly (4 times a week) between T0 and T1, in the 2 groups.<br><br>Exploratory analyses :<br>1- Measurements of the different parameters of **the HD EEG** at T0 and T1 in **the 2 groups.**<br>2- Correlation between **THI score and EEG-HD parameters** at T0 and T1 in the 2 groups.
METHODOLOGY / STUDY DESIGN	Prospective, controlled, randomized, crossover, single-center, single-blind study (blinded evaluator in HD EEG).
CRITERES D'INCLUSION DES SUJETS	1- Person who agreed to participate in the study and gave consent<br>2- Age≥ 18 years old and < 85 years old<br>3- Suffering from subjective tinnitus for more than 3 months<br>4- Have a score on the THI questionnaire carried out at T0 greater than or equal to 38<br>5- Be equipped with a smartphone compatible with the Siopi application and a computer with an internet connection for videoconference sessions<br>6- Have answered the questionnaires on the Siopi application: THI, EVA-G and associated questions, and Insomnia Severity Index<br>7- Have committed to diligently following the study protocol, including independent training<br>8- Have committed not to take any further new tinnitus treatments for the duration of the study<br>9- Able to understand and execute evaluation instructions.
CRITERIA FOR NON-INCLUSION OF SUBJECTS	1- Patient with objective tinnitus<br>2- Current White Noise Generator Port<br>3- Patient who has consulted in phoniatrics more than 2 times or who has participated in more than 2 sessions of group workshops with sound mediation<br>4- Bilateral hearing loss > 40dB not corrected on an audiogram or having reported severe or severe hearing loss, with or without prosthetic correction, on the Siopi questionnaire<br>5- New therapy introduced less than 2 months ago<br>6- Psychiatric disorders causing auditory hallucinations<br>7- Protected person (under guardianship or curatorship)<br>8- Person under protection of justice<br>9- Persons deprived of liberty<br>10- Person not affiliated to a social security scheme<br>11- Pregnant or breastfeeding woman<br>12- Person participating in a drug study

<table>
<tr>
<td>STRATEGIES / PROCEDURES</td>
<td>

RECRUITMENT by the phoniatrist of the ENT department of the Orléans University Hospital

Participants are recruited from the patients of the tinnitus multidisciplinary team of Orleans or through the Siopi application, a mutual aid community for tinnitus patients. After presentation of the study and signing of the consent; and if patients meet the eligibility criteria, they will be randomized to the study and divided into 2 groups: group A and group B (delayed training).

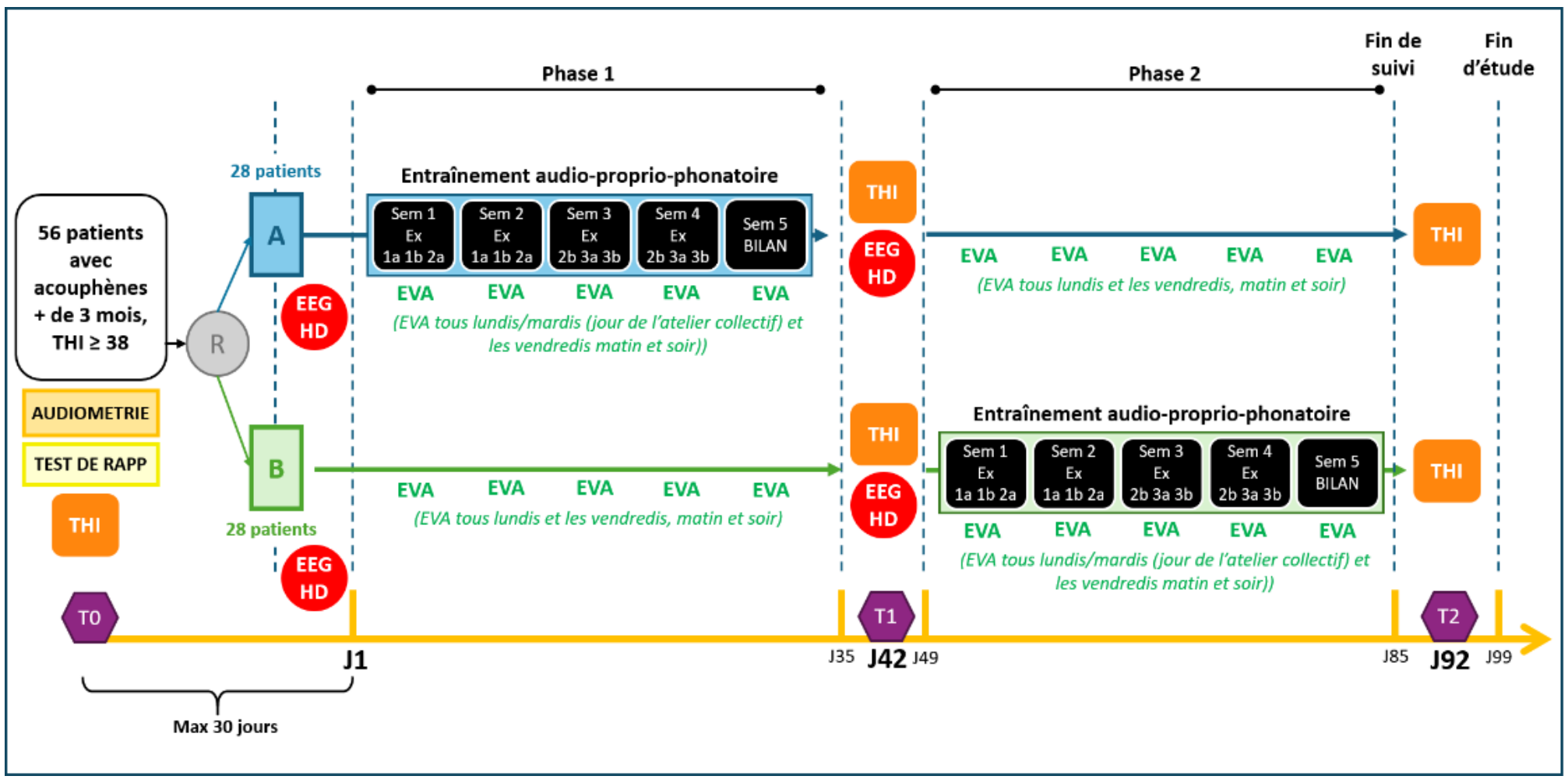

AUDIOMETRIC TESTS in the ENT department of the Orléans University Hospital

HIGH-DENSITY EEG (HD EEG) **in the neurology department of the Orléans University Hospital**

AUDIO-PROPRIO-PHONATORY REINFORCEMENT training: daily sessions by weekly group workshops by videoconference and independent training by video, for 5 consecutive weeks.

</td>
</tr>
<tr>
<td>NUMBER OF PARTICIPANTS</td>
<td>56 tinnitus patients (28 per arm)</td>
</tr>
<tr>
<td>DURATION OF THE SEARCH</td>
<td>Length of Inclusion Period: 36 months
Duration of participation for each patient: 5-6 months
Total duration of the study: 42 months</td>
</tr>
<tr>
<td>EXPECTED BENEFITS</td>
<td>Through this study, we wish to show, on the one hand, the individual benefit of training by audio-proprio-phonatory reinforcement in patients suffering from subjective tinnitus and on the other hand to provide knowledge on the specificity of connectivity by the HD EEG measurement of the different profiles of responsive patients and the involvement of brain plasticity in the favourable evolution.</td>
</tr>
</table>

LIST OF ABBREVIATIONS

ANSM	National Agency for the Safety of Medicines and Health Products
AS	Acoustic Stimulation
ASC	Chronic Subjective Tinnitus
BAPP	Audio Proprio Phonatory Loop
CHU	University Hospital Center
CPP	Committee for the Protection of Persons
CNIL	National Commission for Information Technology and Civil Liberties
CRF	Case Report Form
HD-EEG	High Density Electroencephalogram
ESIT	European School of Interdisciplinary Tinnitus research
EVA	Analogue Visual Scale
fNIRS	functional Near InfraRed Spectroscopy
IR	Residual inhibition
HD-tDCS	High-Definition transcranial Direct Current Stimulation
KHz	Kilohertz
MD	Medical Doctor
MR	Reference Methodology
MUS'E	MUSICOTHERAPIE EXPRESSIONS 45
PH	Hospital Practitioner
RAPP	Phonatory Proprietary Audio Reinforcement
ST	Subjective Tinnitus = acouphène subjectif
TCC	Cognitive Behavioral Therapy
THI	Tinnitus Handicap Inventory
TRI	Tinnitus Research Initiative
TRT	Tinnitus Retraining Therapy
VNS	Vagus Nerve Stimulation

1. GENERAL INFORMATION

1.1. PROJECT LEADERS

TITLE OF THE STUDY: **Neuromodulatory effects of audio-proprio-phonatory reinforcement training on subjective tinnitus, objectified by EEG HD (PHONACOU)**	
SPONSOR :	**Orléans University Hospital**<br>Research and Innovation Department<br>14 avenue de l'hôpital, 45067 Orléans Cedex 02, CS 86709
PRINCIPAL INVESTIGATOR:	**Dr. Isabelle MARIÉ-BAILLY, MD**<br>ENT Department, Orléans University Hospital<br>14 avenue de l'hôpital, 45067 Orléans Cedex 02, CS 86709
ASSOCIATE INVESTIGATORS	**Dr. Canan OZSANCAK, MD, PhD**<br>Department of Neurology, Orléans University Hospital<br>14 avenue de l'hôpital, 45067 Orléans Cedex 02, CS 86709<br><br>**Dr Pascal AUZOU, MD, PhD**<br>Department of Neurology, Orléans University Hospital<br>14 avenue de l'hôpital, 45067 Orléans Cedex 02, CS 86709
ASSOCIATE SCIENTISTS	**Robin GUILLARD, PhD**<br>PhD in Neuroscience, University of Grenoble-Alpes<br>Founder of Siopi, a community of mutual aid for tinnitus patients<br><br>**Mahmoud HASSAN, PhD**<br>Scientist and founder of MINDIG, Rennes, France<br>Assistant Professor, University of Reykjavik, Iceland
METHODOLOGIST/BIOSTATISTICIAN:	**Mathilde WANNEVEICH**<br>Research and Innovation Department<br>14 avenue de l'Hôpital, 45067 Orléans Cedex 02, CS 86709

1.2. SCIENTIFIC COMMITTEE

Non applicable

1.3. OVERSIGHT COMMITTEE

Non applicable

1.4. OTHER COMMITTEES

Non applicable

2. BACKGROUND AND SCIENTIFIC RATIONALE

2.1. DESCRIPTION OF THE PATHOLOGY

Tinnitus is a common symptom (10% of the French population, 1% of whom suffers from it) that is too often minimized by doctors, even by ENT specialists. Patients find themselves alone in the face of their suffering, in great medical wandering.

2.1.1. Definition and types of tinnitus

Tinnitus is defined as the subjective perception of a sound that is not induced by external stimulation. They result from a disorder of the auditory system and/or other structures related to it. Their experience can be embarrassing, thus affecting the person's quality of life (Noreña et al., 2021). Tinnitus is a unified phenomenological coherent perception, linking several separable clinical characteristics, such as its volume, laterality, type (pure tone, noise), associated distress. There are generally two types of tinnitus.

Subjective and objective tinnitus:
Objective tinnitus comes from an internal acoustic stimulation of the body which is most often due to a vascular abnormality; It is a very small minority, affecting only 5% of cases. Subjective tinnitus is only perceived by the affected person and accounts for 95% of cases (Esmaili & Renton, 2018). It can be acute and sometimes disappear or more often become chronic.

Chronic subjective tinnitus:
Chronic subjective tinnitus is when it lasts for more than 6 months, without interruption for 24 hours. This symptom is often an expression of an underlying condition; The most common case is the aftermath of a peripheral lesion, which may be caused by sound trauma that has caused the death of part of the hair cells of the cochlea.

Somatosensory tinnitus:
However, it is important to emphasize the existence of interconnections between auditory pathways and other sensory-sensory systems (Ohresser et al., 2017). This is the trigeminal pathway, innervating the face, jaw and oral structures and the C2-C3 spinal ganglia, innervating the posterior part of the head, pinnae, neck and arms (Wu et al., 2016). Thus, tension in the neck or temporomandibular joint can affect the auditory pathway and cause tinnitus.

2.1.2. Explanatory models of tinnitus

The perception of subjective tinnitus is hypothesized to be the consequence of multiple and intertwined peripheral and central hearing dysfunctions that have only been partially elucidated to date (Langguth et al 2013). Many models have been proposed. For example, tinnitus is thought to appear due to a failure of the limbic region noise suppression system on the central auditory system when the auditory pathways are damaged (Rauschecker et al 2010).

The central gain adaptation model proposed by Norena (2011) assumes that tinnitus arises from an increase in neuronal activity in the central auditory system due to a reduction in the supply from the auditory nerve; in this, it is in line with the model of thalamocortical dysrhythmia described by Llinas et al en 1999, and corroborate by De Ridder et al in 2014. The association with the close frequencies of the cortical neurons

associated with the disappeared area causes overstimulation of the injured frequency, leading to the appearance of a **phantom sound (**Noreña & Farley, 2013; Ohresser et al., 2017).

An analogy between tinnitus and refractory pain postulated as early as 1987 by Tonndorf is based on the deafferentation of nerve fibers. The unmyelinated type II fibers of the auditory nerve play a role analogous to the unmyelinated fibers associated with nociceptive entry into the somatosensory system and are thought to be involved in "auditory nociception". This is analogous to peripheral inflammation that stimulates nociceptors in the somatosensory system to generate pain perception.

A theoretical pathophysiological framework capable of explaining all these aspects was proposed by De Ridder in 2015 integrating the neurophysiological models based on deafferentation and the dysfunctional model of noise cancellation, with a subnetwork of "tinnitus nucleus". The nucleus of tinnitus can be defined as the minimal set of brain areas that must be activated jointly (= subnetwork) for tinnitus to be consciously perceived, devoid of its affective components.

2.1.3. Tools for assessing the impact of tinnitus

In the context of tinnitus, reducing its discomfort means alleviating the discomfort, stress, anxiety, or other negative symptoms associated with its daily perception. Assessing this impact on patients' lives is therefore crucial to determine the most appropriate therapeutic support. The Tinnitus Research Initiative (TRI) recommends assessing the impact of tinnitus by measuring certain parameters considered essential (Ohresser et al., 2017). It is therefore necessary to understand the different dimensions of tinnitus, using an approach combining subjective and objective tools (Nascimento et al., 2019).

2.1.3.1. Evaluations subjectives

To this end, subjective questionnaires play a central role in assessing the impact of tinnitus on patients' quality of life (Ohresser et al., 2017). They are also probing the effectiveness of tinnitus management therapies.

The Tinnitus Handicap Inventory (THI) published by Newman et al. in 1996 is the most widespread. Composed of 25 questions leading to the analysis of associated symptoms, it allows patients to quantify the emotional, functional and catastrophic implications of tinnitus in their lives. However, the results require a weighted, contextualized analysis and consideration of the interaction with the patient, due to the subjectivity of the symptom being assessed (Ohresser et al., 2017). The validated French version THIf (Ghulyan-Bedikian et al. 2010) allows a quantification of the psycho-emotional and functional components of tinnitus-related disability and serves as a tool for evaluating the effectiveness of a therapy and thus contributing to the standardization of tinnitus assessment methods on a global scale.

The Visual Analogue Scales (EVA) invented by Huskisson in 1974, are available in two versions, EVA Nuisance (EVA-G) and EVA Intensity (EVA-I). They help patients express the subjective consequences of tinnitus, rating discomfort and intensity on a scale of 0 to 10.

The standardized history questionnaire (ESIT-SQ) developed by the European School for Interdisciplinary Tinnitus Research (ESIT) and recently translated into French (Guillard et al., 2022) allows the characteristics of tinnitus to be collected in a standardized way to help with patient subtyping.

In addition, digital technologies offer new perspectives for evaluating therapeutic efficacy. This is how we will use **the Siopi mobile application** (Guillard et al., 2023) for the recruitment and monitoring of patients' symptoms through the questionnaires mentioned above.

2.1.3.2. Evaluations objectives

Acouphenometry, performed in addition to tone and vocal audiometry, measures the physical characteristics of tinnitus (frequency, intensity, maskability, residual inhibition (IR)) (Lurquin & Coulon, 2016), reassuring the patient and facilitating the understanding of his pathology, which is now authenticated. It helps to choose therapy and assess the potential masking of tinnitus with hearing aids.

The spectrogram translates the sounds picked up by a microphone into a physical image in real time and visualizes its acoustic characteristics, including its fundamental frequency and harmonic frequencies.
In vocal rehabilitation, it is very useful for reinforcing the audio-phonatory loop of patients by audio-visual feedback (or feedback) (Parmentier, Klein-Dallant, 2016). Speech therapists use Praat, Vocalab (Menin-Sicard, A. et al., 2003) or VoceVista software developed by D. Miller, which allows the user to view his spectrogram in real time (DeJonckere, 2016). In tinnitus, the spectrogram allows the investigator and the patient to facilitate, thanks to visual feedback, the work of frequency discrimination. Thus, the objectification of the tinnitus frequency by autophonation guided by the spectrographic image can be similar to acouphenometry (Marié-Bailly, 2016).

Brain imaging, in HD EEG, FMRI or fNIFRS have demonstrated that the appearance of tinnitus results from a dynamic process resulting in aberrant local neural activity and abnormal connectivity in multifunctional brain networks. We will detail them below by arguing the choice of HD EEG in our clinical study, to objectify the impact of cognitive training by RAPP.

2.2. RATIONALE AND DESCRIPTION OF THE STRATEGY STUDIED

TRT sound therapy (Tinnitus Retraining Therapy), offered by hearing care professionals, is one of **the pillars of support for people with tinnitus**. This therapy can use white noise generators alone or combine them with hearing correction.

Complementary therapeutic approaches approved by AFREPA, the French-speaking Association of Multidisciplinary Teams in Acouphenology (sophrology, hypnotherapy, psychology, cognitive behavioural therapy, osteopathy, physiotherapy) can be added and work on **psychological and somatosensory factors, which are often as important as auditory factors**.

Cognitive training by audio-proprio-phonatory reinforcement (RAPP) in "tinnitus", combining phoniatrics and music therapy, acts on these multiple factors. She offers tinnitus patients various multisensory proprioception exercises that promote relaxation in the ENT sphere and allow them to be their own transmitter of sound stimuli, by discriminating frequencies, to mask their tinnitus or produce residual inhibition, without the contribution of an external stimulus.

This clinical study will explore how training to improve coordination between the auditory, proprioceptive and phonatory sensory systems could impact patients' perception and management of tinnitus

2.2.1. The audio-phonatory loop

The audio-phonatory loop is a cognitive skill that regulates the adjustment of one's own vocal and articulatory productions through a process of **voice feedback, called auditory feedback** (Aglieri et al., 2018). The feedback that allows this voice self-regulation system can be internal (proprioceptive and bone-based hearing) but also external (airborne hearing).

The process of feedback of vocal and articulatory production uses **proprioceptive feedback**, explaining how awareness of the postures, movements and vibrations that accompany the vocal gesture is essential in the audio-phonatory loop (Borel & Leybaert, 2020). It is the positions of the tongue, lips, soft palate, vocal folds and arytenoid cartilages that allow instantaneous adjustments during phonation. In order to further emphasize the fundamental role of proprioceptive feedback in the articulation of sounds, it would therefore be appropriate to speak of an "audio-proprio-phonatory loop".

2.2.2. Audio-proprio-phonatory reinforcement – RAPP

Thus, **audio-proprio-phonatory reinforcement** refers to a process of consolidation of connections and coordination between the auditory, proprioceptive (which concerns the perception of body movements and positions), and phonatory (relating to the production of speech) systems. It is therefore a question of working on the relationship between hearing, the sensory perception of the body, and the production of speech or voiced or unvoiced sounds. However, the perception of tinnitus is likely to affect all auditory perceptions, and in particular the audio-proprio-phonatory loop (Lima et al., 2020).

Auditory discrimination training can be considered a form of **sound therapy** – through listening to Zen sounds (Jin et al, 2021) or music in the opposite phase of tinnitus sound (Ni et al, 2024) – that can decrease the perception of tinnitus, as patients become able to focus on other sound stimuli. In addition, this decrease in attention to tinnitus can also be accompanied by an improvement in the patient's quality of life (Rodríguez-León et al., 2022). Moreover, Dupont & Lejeune (2021) emphasize that this approach results in "the establishment of new perceptual habits, new perceptual contrasts between speech sounds and eliminating phonetic confusion as much as possible". In this sense, the patient learns to focus on other stimuli, including the self-perception of his voice. Auditory discrimination training thus promotes the patient's ability to analyze and modify his or her self-emitting sounds and access helpful masking.

Indeed, it appears that audio-proprio-phonatory reinforcement can play a significant role in alleviating tinnitus-related discomfort. The use of **self-vocalization** to create an individual sound bath could promote the habituation of the brain, creating an **attentional defocus** and thus **reduce the psychological impact of tinnitus**.

In addition, proprioceptive and articulatory sensations are also targeted to improve the efficiency of movements and reduce muscle tension. We also take advantage of the potential stimulation of the **position of the hands in "amplifier shells",** alternately, in each ear to **reinforce auditory feedback through the air, or through the bone, by blocking one or the other ear.**

In addition, **feedback from the audio-proprio-phonatory loop can be reinforced by the visual feedback offered by a spectrogram** to improve awareness and vocal control (Parmentier et al., Menin Sicard et al., 2021), but also to produce sound reinforcements close to the tinnitus frequency to produce residual inhibition.

Cette approche, associée à une **implication active du patien**t, offre un potentiel considérable pour améliorer la qualité de vie des personnes atteintes d'acouphènes

2.2.3. Search for Residual Inhibition

Stimulation of residual inhibition (IR) can be a key component in temporarily reducing the perception of tinnitus. Indeed, this **psychoacoustic phenomenon results in a short silence perceived just after exposure to a sound**. According to Neff et al., (2019), the achievement of this short silence seems to be potentiated by sounds located in the frequency spectrum of tinnitus: sounds modulated in intensity and frequency seem to be more effective and better tolerated than constant sounds. Acouphenometry is therefore essential to the personalization of therapeutic sound (Lurquin & Coulon, 2016).

In research in acouphenology and auditory cognitive neuroscience, tinnitus masking and residual inhibition (IR) are two psychoacoustic measures made from acoustic sequences composed of pulsed or continuous stimuli. IR predicts the success of masking therapy, which is more effective with noise emitted close to tinnitus frequencies (Fournier et al., 2018). Hence the interest of acouphenometry to visualize the loudness of tinnitus.

Although the use of the voice to produce residual inhibition has not yet been explored as such, the production of self-masking was observed during the audio-vocal therapies of Marié-Bailly and Varquet (2020), which was based on the self-emission of white noise close to the auditory disturbances of tinnitus. Indeed, the voice can be worked on in such a way as to modify its spectral characteristics. In addition, the spectrogram can provide visual feedback of the acoustic parameters of the voice (Parmentier et al., 2014) which could be useful to produce **sound reinforcements close to the tinnitus frequency** and thus for the modulation of the perception of tinnitus.

Beyond the temporary relief it provides, obtaining residual inhibition from one's own voice could provide avenues for a better understanding of the pathophysiological mechanisms of tinnitus, but also refine sound therapies, such as auditory discrimination training stimulating the audio-proprio-phonatory loop, by maximizing their inhibitory effect on tinnitus**.**

2.3. SUMMARY OF RESULTS FROM AVAILABLE NON-CLINICAL TRIALS AND CLINICAL TRIALS

Among the recent clinical studies carried out, we present those that seem to us to be the most significant for our own research.

2.3.1. 1st research: SCED study for a speech therapy thesis

To make progress on this issue, we conducted a first SCED (Single-Case Experimental Design) study in 2023-2024 with 3 patients as part of a speech therapy thesis , in order to observe the **effect of strengthening the audio-proprio-phonatory loop on the discomfort related to chronic** subjective tinnitus, while evaluating the possibility of producing residual inhibition from listening to one's own sound systems. At the time, evaluation tools were subjective, such as the THI and the VAS to measure the effects of the intervention using the SIOPI mobile application, as mobile applications provide real added value to tinnitus treatments and effectiveness evaluations. This study was presented at the conference-debate "Let's tame tinnitus in interdisciplinarity" on June 15, 2023 at the Orléans University Hospital.

As part of our clinical study, to optimize the results, we opted for:

- The increase in the statistically significant number of patients included,
- A 2-arm, randomized, stratified,
- The resumption of the same RAPP exercises by diversifying them,

- Training through group workshops promoting group dynamics, alternating with independent video sessions, conducive to the assimilation of exercises,
- The support of the Siopi platform, for information and subjective evaluation tests,
- Objectification by HD EEG, thanks to a new platform soon available in the neurology department

2.3.2. Objectification of connectivity in neuroimaging

Functional spectroscopy in the near infrared – fNIRS uses near-infrared light to measure cortical hemodynamic responses considered as an indirect marker of local neuronal activation. A study in sound therapy in tinnitus patients using fNIRS (Huang et al., 2022), demonstrated that **notched sound** - whose tinnitus frequency has been removed from the spectrum - improves tinnitus by regulating not only the auditory center through lateral inhibition, but also by reorganizing the **emotional control center.**

Functional magnetic resonance imaging (fMRI) performed in military adults with tinnitus after acoustic trauma (Job et al., 2012) suggests that tinnitus is a proprioceptive illusion caused by abnormal excitability of the muscle spindles of the middle ear, associated with **emotional and sensorimotor** disturbances; This hypothesis was corroborated in 2016 by the probable existence of a somatosensory pathway encompassing the trigeminal system. A review of fMRI studies (Jarowski et al., 2022) highlights the role of the OP3 right parietal operculum subregion in the integration of auditory and somatosensory representation of orofacial muscles in the healthy population.
These data open therapeutic perspectives using brain plasticity to modulate the annoying perception of tinnitus, through **the auditory-somatosensory approach.**

The High-Density Electroencephalogram - HD EEG can provide objective measures of symptom severity and quantify the effectiveness of a treatment. Recent studies (Lan et al, 2021) (Huang et al, 2021) show that patients with chronic tinnitus have increased connections between the parahippocampal gyrus, posterior cingulate cortex, and precuneus compared to the healthy group, which corresponds to the default mode network and that the areas are mainly concentrated in the right temporal lobe as well as in the temporal lobe, the parietal lobe and the frontal area.

In 2024, Vanneste demonstrates that increased alpha activity in the dorsal anterior cingulate cortex is correlated with distress, by a **mechanism parallel to chronic neuropathic pain and suggests neuromodulatory tinnitus treatments by promoting** alpha activity in the anterior part of the pregenual cingulate cortex and reducing it in the dorsal anterior cingulate cortex.

It is through this neuromodulation process that RAPP training could act favourably, by reducing the painful perception of phantom sounds and be offered in the simple and ecological therapeutic support of people suffering from tinnitus.

2.3.3. Neurostimulation and neurofeedback therapies

In the literature on tinnitus, researchers are increasingly advocating a distinction between the perception of tinnitus and tinnitus-related distress, suggesting the role of neuroplasticity in auditory nociception (Shore et al., 2016), hence the interest of cognitive stimulation approaches.

Vagus nerve stimulation (VNS) activates the neuromodulatory pathways of the forebrain cholinergic system and the noradrenergic system. Studies in rats (Engineer et al., 2013) suggest that sound-paired VNS may restore normal neuronal activity after noise-induced trauma.

Repeated transcranial magnetic stimulation (rTMS) is a non-invasive method of modulation of brain activity first described in 2003, whose clinical relevance remains to be demonstrated; a systematic review of studies (Landero et al, 2016) confirms a possible efficacy of rTMS with low-frequency stimulation parameters (1 Hz) targeting the temporoparietal cortexes.

Intracranial electrical stimulation, especially bimodal stimulation, appears to be a promising neuromodulation; a recent study (Martin et al, 2022) seems to help define tinnitus subtypes. Intervention protocols (Ghodratitoostani et al, 2022) provide reliable evidence to approve or not the efficacy and safety of this method.

Electrical stimulation of the cochlear nerve, via cochlear implants or extra-cochlear stimulation, has been shown to be beneficial in reducing tinnitus discomfort (Assouly et al, 2021).

Functional fMRI with neurofeedback allows the patient to learn to voluntarily control the activity of distinct brain areas. A study comparing the effectiveness of neurofeedback fMRI to CBT shows a reduction in the severity of severe chronic tinnitus, persisting at 12 months.

Electroencephalography neurofeedback - EEG-NFB is a process of learning, neuromodulation and conditioning of patients. This validated therapy for attention deficit hyperactivity disorder (Arns et al 2009) also can reduce tinnitus-related discomfort (Vanneste et al, 2018b; Güntensperger et al, 2020). A recent study (Guillard et al, 2020) tested the feasibility and effectiveness of a wearable device used by patients at the hearing care professional's office.

2.3.4. Cognitive learning by audio-proprio-phonatory feedback

RAPP training uses **a process like NFB with the advantage of being environmentally friendly** since the learning process modulating brain activity comes from the patient's feedback control of the discrimination of his or her own movements, whether it is sounded. It is also more reliable because the HD EEG measurement is very noisy.

Our operational mode is also part of an **audio-somatosensory dynamic** described in the articles above. Indeed, through its repeated and targeted auto-phonations, the tinnitus patient reinforces the process of auditory discrimination of harmonic frequencies due to the contraction of the external cilated cells that amplify the sound signal received via the tympano-ossicular chain. In addition, the proprioceptive, articulatory and/or phonatory movements of the tongue, lips and larynx contribute to the relaxation of the entire ENT sphere and the orofacial and cervico-laryngeal muscles, very often contracted due to the nociceptive feelings of tinnitus.

In addition, RAPP exercises promote the **voluntary control of the neurovegetative function of breathing**, proposed by psycho-somato-sensory approaches, such as sophrology or hypnosis, recognized by AFREPA. Through his repeated and targeted auto-phonations in their modulations, the patient is invited, between control and letting go, on the one hand to become aware of the autonomous costo-diaphragmatic inspiratory

relaxation, and on the other hand, to regulate his expiratory breath in its flow, his pressure by playing on the intensity, the pitch of the sounds as well as its duration.

Thus, in addition to its audio-somatosensory action, RAPP training, like CBT, neurofeedback therapies or sound therapies, teaches to voluntarily control the activity of distinct brain areas, to broaden the attentional field, and thus to **reduce the emotional impact of tinnitus sound stimuli.**

These various experimental research studies and clinical studies also confirm our choice to try **to objectify the effect of cognitive training by studying brain connectivity, based on HD EEG technology, which is** non-invasive, effective, easy to use and recently available on the neuroimaging platform of the neurology department of the ORLÉANS University Hospital.

Through this clinical study, we therefore wish to demonstrate the beneficial effect of cognitive training promoting neuroplasticity by audio-proprio-phonatory reinforcement in patients suffering from subjective tinnitus.

This, completed and objectified by EEG HD, by researching whether there is a link between the evolution of connectivity and the favourable response to the therapeutic protocol.

3. RESEARCH OBJECTIVES

3.1. MAIN OBJECTIVE

To determine whether audio-proprio-phonatory reinforcement (APR) training reduces tinnitus-related disability in daily life compared to patients who have not undergone RAPP training.

3.2. PRIMARY OUTCOME

We will use the **international Tinnitus Handicap Inventory (THI) questionnaire** to assess the effect of training on the impact of tinnitus on patients' quality of life (Appendix 3).
The difference in the THI score between T1 and T0 obtained in the group that underwent RAPP training compared to the difference in the THI score between T1 and T0 obtained in the group without RAPP training will be compared.

3.3. SECONDARY OBJECTIVES

1- To determine whether RAPP exercises reduce tinnitus-related discomfort in the short term (EVA Tinnitus Discomfort Scale), compared to patients who have not undergone RAPP training.
2- To study the stability over time of quality of life (THI score) and tinnitus-related discomfort (VAS scale) after RAPP training.
3- Describe the change in patients' quality of life (THI score) over time.
4- To describe the evolution of other tinnitus collateral discomforts (noise, cervical, jaw, sleep, stress-anxiety) in patients who have undergone RAPP training, compared to patients without RAPP training.

Exploratory analyses in HD-EEG will also be carried out to:

1- To assess the effect of RAPP training on the usual connectivity and microstate parameters of HD-EEG, compared to patients who have not undergone RAPP training.
2- To describe the evolution of the quality of life (THI score) of patients before and after RAAP training and the usual parameters of connectivity and HD EEG micro-state, over time.

3.4. SECONDARY OUTCOMES

1- Tinnitus VAS-G (VAS) visual analogue scales, completed by the patient (on the Siopi application), weekly between T0 and T1, at the rate of 4 times a week:

- For the group with RAPP training between T0 and T1, they will be completed: morning and evening, on the day of the group workshops (Monday or Tuesday) and on Friday
- For the group without RAPP training between T0 and T1, they will be completed: on waking up and in the evening, every Monday and Friday of the week.

First, it will be a question of comparing the average of the daily differences (VAS-G tinnitus evening – VAS-G tinnitus morning) obtained over 5 weeks (with RAPP training for group A *vs* without RAPP training for group B).
Then we will compare the evolution (taking the daily average of the tinnitus-VAS-G) over the 5 weeks between T0 and T1 for the 2 groups (i.e. 10 measurements per group).

NB: These results can be confirmed by analyzing the tinnitus-VAS-G over the 5 weeks between T1 and T2 for group B (having RAPP training over this period).

2- To study stability over time, only data from patients **in group A,** without RAPP training **between T1 and T2** will be used:

- Tinnitus EVA G scales, completed by the patient (on the Siopi application), weekly between T1 and T2, at a rate of 4 times a week.
- The THI score measured at T1 and T2

It will be a question of comparing the results at T2 compared to T1 (for the VAS we will take the average on the 1st day and on the last day of the 5 weeks between T1 and T2).

3- Evolution over time of the THI score measured at T0, T1 and T2 for the 2 groups.

4- Analogue visual scales EVA-G associated questions (noise, cervical, jaw, sleep, stress-anxiety) completed by the patient (on the Siopi application), weekly **between T0 and T1**, at the rate of 4 times a week, for the **2 groups**.

We will compare the evolution of each VAS-G scale taken independently, taking the daily average of the VAS-G concerned, over the 5 weeks between T0 and T1 for the 2 groups.

5- Exploratory analyses of the different parameters of **the HD EEG** measured at T0 and T1 in **the 2 groups:**

- The evolution of the connectivity and microstate parameters of the HD-EEG (measured at T0 and T1) such as: network efficiency, shortest network, duration of states, distribution of states, etc.; will be described in the group with RAPP training and compared to the evolution of these same parameters in the group without RAPP training between T0 and T1.
- The search for correlation between the evolution of connectivity parameters with the evolution of quality of life (THI score) will be explored (between T0 and T1).

4. Study population

4.1. Population Description

The subjects included in this study will be people with unilateral or bilateral tinnitus, of indifferent sex, meeting the following criteria.

4.2. Inclusion criteria

1- Person who agreed to participate in the study and gave consent
2- Age≥ 18 years old and < 85 years old
3- Suffering from subjective tinnitus for more than 3 months
4- Have a score on the THI questionnaire carried out at T0 greater than or equal to 38
5- Be equipped with a smartphone compatible with the Siopi application and a computer with an internet connection for videoconference sessions
6- Have answered the questionnaires on the Siopi application: THI, EVA-G and associated questions and Insomnia Severity Index
7- Have committed to diligently following the study protocol, including independent training
8- Have committed not to take any further new tinnitus treatments for the duration of the study
9- Able to understand and execute evaluation instructions.

4.3. Criteria for non-inclusion

1- Patient with objective tinnitus
2- Current White Noise Generator Port
3- Patient who has consulted in phoniatrics more than 2 times or who has participated in more than 2 sessions of a group workshop with sound mediation
4- Bilateral hearing loss > 40dB not corrected on an audiogram or having reported severe or severe hearing loss, with or without prosthetic correction, on the Siopi questionnaire
5- New therapy introduced less than 2 months ago
6- Psychiatric disorders causing auditory hallucinations
7- Protected person (under guardianship or curatorship)
8- Person under protection of justice
9- Persons deprived of liberty
10- Person not affiliated to a social security scheme
11- Pregnant or breastfeeding woman
12- Person participating in a drug study

Our clinical research investigates how individual and group RAPP training influences psychological, physiological, and neuromodulatory outcomes. Therefore, we do not exclude patients with organic, metabolic, or psychological disorders, except for those experiencing auditory hallucinations, as these may impact EEG-HD data.

During medical recruitment consultation, audiometry with acouphenometry assesses the side, intensity, and frequency of tinnitus, residual inhibition, and somatosensory factors like SADAM, SROS, bruxism, and cervical disorders. The EVA discomfort test tracks changes in tinnitus-related discomfort and five other criteria

(hyperacusis, jaw issues, cervical problems, sleep, anxiety) throughout the protocol, highlighting symptoms that may influence RAPP training outcomes.

4.4. RECRUITMENT PROCEDURES

Patient recruitment will be carried out in the ENT department of the ORLÉANS University Hospital, through posters (appendix 1), supplemented by information on the Siopi platform, on the website of the Orléans Tinnitus Multidisciplinary Team, on the MUS'E association website and on social networks (appendix 1).
Patients will be informed of the study and its objectives by oral explanations (investigator) and an information letter will be given to them. They will be able to ask any questions they may have to the study investigator. If they agree to participate, they will sign the consent form.

4.5. BENEFIT/RISK

This study is a category 2 human research project.

Throughout the study, no major risks were incurred by the subjects. Audiometric tests are routine ENT examinations as well as the use of the spectrogram in phoniatric consultations or speech therapy rehabilitation. The audio-proprio-phonatory training procedure is a minimal constraint, whether in a group workshop or independently.

No specific physical risks or constraints related to the EEG-HD examination are foreseen for the participants. This technique is non-invasive and does not present any danger, except for possible skin irritation (mentioned in the equipment manufacturer's data sheet: EGI©) occurring exceptionally when in contact with the electrodes. Possible fatigability during the different sessions of the experiment could occur and will be monitored. A break will be offered if necessary. At the end of this experiment, the participant's hair may be a little sticky due to the saline solution. Nevertheless, this can be easily removed with pure water. In addition, the electrodes are likely to leave small marks (due to the solution) but these will disappear quickly.
The direct benefit is the possible improvement in their quality of life linked to the effect of RAPP exercises on the perception of their tinnitus, but also to the gratifying feeling of being listened to and understood within a caring group and of being autonomous, an actor in their health.

5. Research Design - Methods

5.1. Research Methodology

Prospective, controlled, block-randomized, two-sequence, two period crossover, single-center study. Participants will be randomly assigned (1:1) to one of two sequences.
Group A: participants will first undergo a 5-week RAPP training period, followed by a 5 week no-training period
Group B: participants will first undergo a 5-week no-training period, followed by a 5-week RAPP training period
Randomization will be stratified on the THI score (grade 3 *versus* grades 4 and 5).
HD EEGs will be performed by staff from the neurology department of the Orléans University Hospital, without knowledge of the patients' treatment arm (blinded).

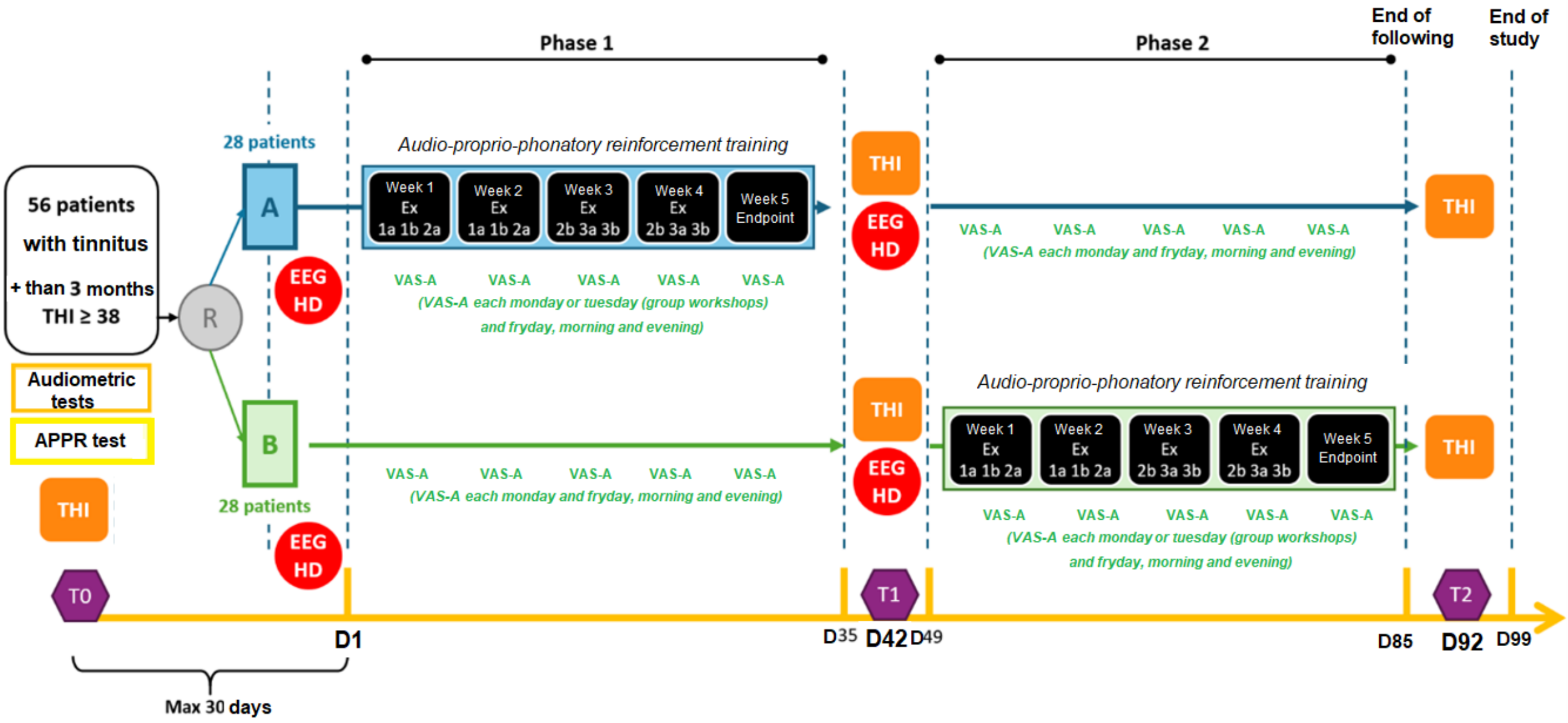

<u>Figure 1 : Study design</u>

This crossover design was chosen for ethical reasons to ensure that all participants have access to the RAPP training. However, the duration and persistence of the intervention effects are currently unknown, and a carry-over effect between periods cannot be excluded. Therefore, all outcomes comparing APPR training versus no-training conditions will be based on the first study period only (T0 to T1) between the two group.
The full crossover structure will be used to explore the temporal dynamics and persistence of the intervention effects.

5.2. Specific Procedures for Research

This study will be offered to tinnitus patients followed for this pathology. The proposed reviews in this study will be research-specific and will include:

- Self-report questionnaires (at visits T0, T1 and T2)
- Medical visits (at T0, T1 and T2 visits)
- RAPP tests with spectrogram feedback (at the T0 visit)
- Acouphenometry with search for residual inhibition (at visit T0)
- Audio-Proprio-Phonatory Reinforcement (APR) training for 5 weeks, through group videoconference workshops and independent sessions (phase 1 or phase 2 depending on the randomization arm)
- High-density electroencephalograms (at visits T0 and T1)

Nevertheless, it includes procedures identical to those of the usual management of tinnitus, such as self-report questionnaires and rating scales (at baseline) and audiometric tests.

5.2.1. Self-report questionnaires and rating scales

5.2.1.1. Application Siopi

Some questionnaires/scales will have to be filled in directly by patients in the Siopi application (see next section). This application is a sharing community on tinnitus, created with the support of AFREPA by a group of French researchers, where patients can share their opinion on different therapies, answer questionnaires, to advance European research, particularly in the understanding of the different types of tinnitus (Appendix 2).

It is therefore an essential criterion for the inclusion of any volunteer subject in our clinical study who will have to download the Siopi application free of charge and then answer the questionnaires/scales.

The Siopi application will also facilitate the dissemination and recruitment of tinnitus patients.

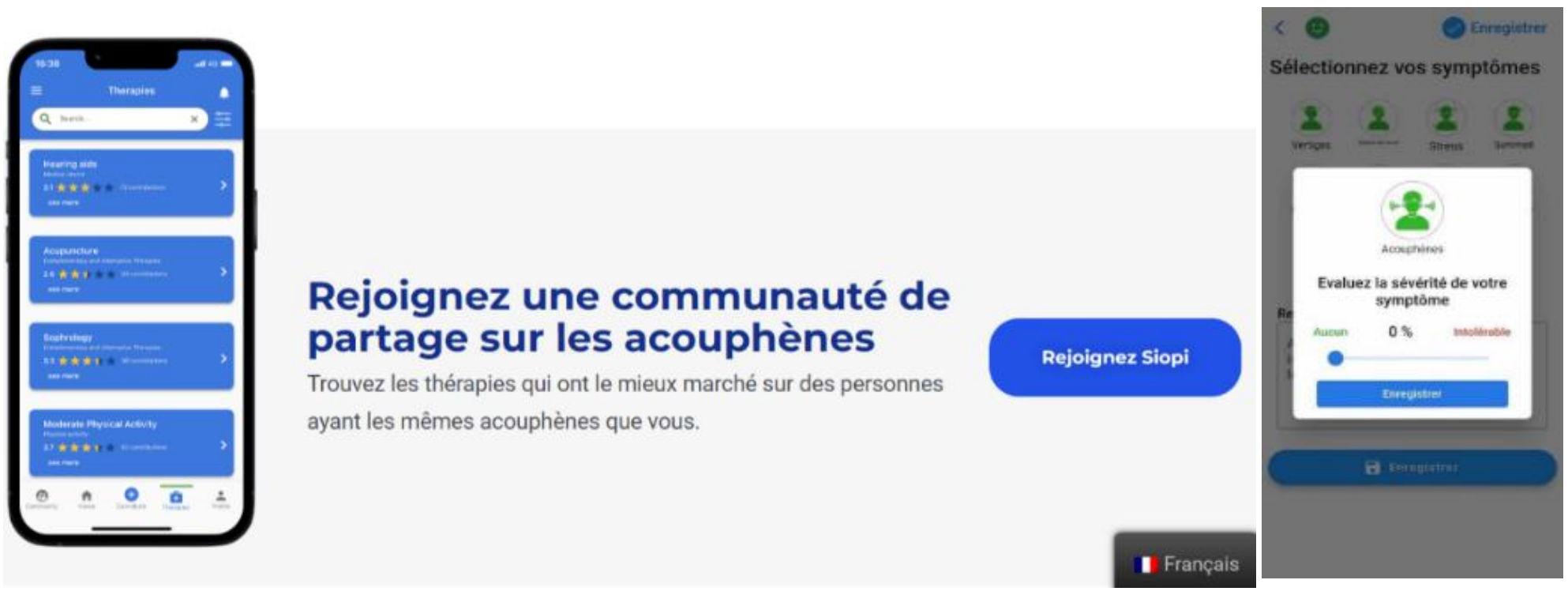

Figure 2: Siopi application (and appendix 2)

5.2.1.2. Assessment of the pathology and associated questionnaires

Of the 9 questionnaires/scales of the Siopi application to which patients will be able to answer freely**, only 3 will have to be completed before inclusion; As a** guarantee of their motivation, they will provide useful information to the investigator such as the perceived severity of the tinnitus-related disability (inclusion criteria):

- ***THI "Tinnitus Handicap Inventory" questionnaire*** (Appendix 3): This international standardized questionnaire is composed of 25 items (rated from 0 to 4) divided into 3 subscales (functional, emotional, catastrophic), (Newman et al. 1996). It determines the perceived severity of the tinnitus-related disability, based on 5 grades from 0 to 100:
    - **Grade 1 - 0 to 16, Lightweight:** only heard in quiet environments; very easily masked. No impact on sleep and daily activities.
    - **Grade 2 - from 18 to 36, Low**: easily masked by the sound environment and easily forgotten during daily activities. Can sometimes disturb sleep but not activities.
    - **Grade 3 - from 38 to 56, Medium:** can be perceived even in the presence of a moderately noisy sound environment. Daily activities can still be performed.
    - **Grade 4 - from 58 to 76, Severe**: almost always heard and rarely masked. Causes disturbances in sleep and can decrease the ability to perform daily activities. Harmful consequences on quiet activities.
    - **Grade 5 - from 78 to 100, Catastrophic**: still heard, disturbed sleep, difficulty with any activity.

The THI questionnaire will be completed at each medical visit at T0, T1 and T2, by the patient on the Siopi application in the presence of the investigator who will keep track of it in the medical file.

At T0, the THI value (reference value) will determine the patient's possibility of inclusion, if equal to or greater than 38 (grades 3, 4 or 5); grades 1 and 2 will be excluded from the protocol to obtain more significant gains. The values recorded at T1 and T2 will allow the longitudinal monitoring of the evolution of their quality of life throughout the study.

- ***Visual Analogue Embarrassment Scale (VAS-G) and associated questions***, to assess the level of discomfort felt by the patient. It is an analogical scale from 0 (absence of discomfort) to 10 (maximum imaginable discomfort) (Appendix 4).
    - During visits at T0, T1 and T2, the patient will complete the values of the VAS-G tinnitus scale and the associated scales on the Siopi application (**only** 5 questions n°2, n°3, n°5, n°7 and n°11, see appendix 4)
    - During phase 1 and phase 2, the VAS-G and the 5 associated questions will also be completed by the patient, 4 times a week, still on the Siopi application, to allow longitudinal monitoring of the evolution of the different symptoms:
        - During RAPP training (phase 1 for arm A and phase 2 for arm B):
            - The day of the group workshop (Monday or Tuesday) when you wake up and after the session (in the evening)
            - Friday when you wake up and after the session (in the evening)
        - During non-training times (phase 2 for arm A, phase 1 for arm B):
            - Mondays, when you wake up and, in the evening,
            - Fridays, when you wake up and, in the evening,

- ***Insomnia Severity Index*** (Appendix 5) allows us to evaluate the level of insomnia of patients over the last few months at T0. It supplements the EVA sleep discomfort data and consists of 7 items.

5.2.1.3. Phoniatric clinical examination in tinnitus

Other questionnaires, scales or tests will be carried out by the investigator at the T0 inclusion visit (standard practice) and will assess the hearing discrimination of the patients as well as their laryngeal functions:

1- Evaluation of vocal timbre via the GRABS-I scale. This scale is composed of 6 items rated from 0 to 3: 0 (normal), 1 (mild), 2 (medium), 3 (severe) (Appendix 6).
2- Evaluation of voice-related disability via the VHI-10 questionnaire (Voice Handicap Index 10). This questionnaire is composed of 10 items rated from 0 to 4 (Appendix 7).

The RAPP test with feedback control by spectrogram (see section 5.2.3) will also be performed at visit T0 (practice added).

5.2.2. Audiometric Testing

These tests will be carried out at the ENT department of the ORLÉANS University Hospital only at the inclusion visit (T0). As part of the study, 3 tests will be performed: a tonal audiogram, a vocal audiogram and an acouphenometry with search for residual inhibition. The equipment used in the service will be of the AC40 audiometer type (Appendix 8).

Tone audiometry is a routine examination that allows you to find out at what minimum intensity a sound is perceived at various frequencies (pitch). It takes place in a soundproof cabin. The person wears headphones where sounds of varying intensity and frequency are broadcast in one ear and then in the other. The person

gives a signal (he raises his hand for example) as soon as he perceives a sound. The tonal audiogram will be measured in decibels. The values analyzed will be on the 5 frequencies: 500, 1000, 2000, 4000 and 8000 Hz. At the inclusion visit at T0, audiometry gives an exclusion criterion if the hearing loss is bilateral greater than 40 dB and not fitted. It is essential to explain to the patient the appearance of tinnitus on the frequency where there is hearing loss.

Speech audiometry tests the level of comprehension and articulation of words using headphones or a speaker. This is done by studying the person's ability to understand speech based on its intensity. Speech audiometry is measured in decibels and produces an intelligibility curve showing, for each sound intensity, the percentage of phonemes, words, or sentences recognized—a key factor in assessing auditory discrimination.

Acouphenometry consists of characterizing tinnitus; it should preferably be performed in high definition, to the nearest Hertz and to the nearest decibel, to detect micro-hearing loss and correlate it with the frequency of tinnitus. The main objective of acouphenometry is to determine a sound that is closest in sound sensation to the most disturbing tinnitus. Specifically, the search for residual inhibition (IR) will be added, which makes it possible to observe the behaviour of the tinnitus after sound stimulation identical to the audiometric spectrum of the tinnitus. Indeed, the perception of tinnitus can decrease or even disappear for a few seconds after auditory stimulation.
The analysis of the data will be based on whether an IR exists, and if so, its duration in seconds or minutes.

5.2.3. RAPP Tests with Spectrogram Feedback

The objective of this test is to evaluate, with the patient, his ability to discriminate audio-proprio-phonatory, the frequency of his tinnitus, as well as the existence of residual inhibition. They correspond to exercises proposed during RAPP training, which allows the patient to understand the objectives and means.

- The first part of the test consists of the taking of 4 voice examples (already pre-recorded), which the patient must reproduce as faithfully as possible:
  - Only after listening (2 times)
  - Then helped by the visualization (2 times) of the frequencies on the spectrogram (appendices 9 and 10).

The examples to be reproduced are sonorizations on unvoiced sibilant constrictive consonants, on high frequencies, oscillating between 3000 and 8000 Hz (identical to those of most tinnitus).
For each test, the investigator will assign, depending on the quality of the vocal example, a score from 0 to 5, which will give after the 4 tests a score out of 20, objectifying the patient's ability to imitate sound by audio-proprio-phonatory discrimination.

- In the second part, the patient makes his own auto-phonations with feedback from the spectrogram:
  - First by trying to discriminate the frequency of his tinnitus
  - Then by sounding for 1 minute on the frequency of his tinnitus, in search of a residual inhibition, i.e. the disappearance of his tinnitus.

The investigator will note the existence or absence of residual inhibition and its possible duration, a criterion for predicting a good response to RAPP training; This examination will be repeated in acouphenometry.

5.2.4. RAPP Training

To follow the Audio-Proprio-Phonatory Reinforcement (RAPP) training, it is essential that the patient has a laptop with an internet connection because the workshops of this training are done by videoconference and video.

It is a cognitive training that promotes neuroplasticity through a process of consolidation and coordination of connectivity between the auditory, proprioceptive, somatosensory (which concerns the perception of movements and positions of the body), phonatory and articulatory systems (relating to speech production).

The principal investigator has been experimenting with this type of RAPP training for more than 20 years in phoniatric consultations for the rehabilitation of communication disorders but also during musicophonology sessions within the framework of his association MUS'E (https://muse45.org/), created in 1991 and approved since 1995 by the Ministry of Education, Youth and Sports and referenced as a Continuing Education Organization.
RAPP training was the subject of a SCED-type clinical study for a speech therapy thesis. https://www.equipeacouphenes-orleans.com/_files/ugd/7635ae_49971a1dbcd44467a1ce609ff5308764.pdf

For the clinical study, the 5-week RAPP training includes sessions to be performed every day of the week from Monday to Sunday (7 sessions per week):

- 1 session in a group workshop by videoconference, at the beginning of the week (Monday or Tuesday)
- 6 sessions in autonomy with the help of videos using the exercises worked on in the workshop, the other days of the week.

5.2.4.1. Types of RAPP Training Exercises

Acouphenometry consists of characterizing tinnitus; it must be carried out in high definition, at Hertz
RAPP training is structured around 3 types of RAPP exercises, where the patient is the "actor" and his or her own transmitter of multisensory stimuli:

- ✓ *Type 1 exercises (small and slow non-sound movements of the tongue and lips, strengthening proprioception):*
  1a: multi-directional scanning promotes relaxation of the oro-maxillofacial sphere
  1b: on the articulatory positions of the different phonemes, consonants and vowels

- ✓ *Type 2 exercises (auto-phonations promoting the defocusing of attention and reinforcing auditory and somatosensory discrimination, on the vowel triangle, appendix 9):*
  2a: by unvoiced phonemes, i.e. without the vocal cords
  2b: with the vocal cords attached, with or without nasalization

- ✓ *Type 3 exercises (looking for residual inhibition from auto-phonations targeted at the frequency of tinnitus, (exercise identical to the RAPP test)):*
  3a: on continuous phonemes
  3b: on discontinuous phonemes

For exercises 2 and 3, the feedback or auditory feedback of the auto-phonations will be increased:

- Either by the position of the hands in an amplifier shell around one ear, alternately to the right and left to amplify the return by air,

- Either by plugging one or the other ear with the hand to increase the auditory feedback through the bone of nasal vowels or consonants.
- By visual feedback on the spectrogram (Voce Vista software, appendices 10 and 11)

Here is the detailed schedule of the weeks for the RAPP training

Five week Audio-Proprio-Phonatory Reinforcement (RAPP) training				
WEEK 1	WEEK 2	WEEK 3	WEEK 4	WEEK 5
Ex 1a 1b 2a	Ex 1a 1b 2a	Ex 2b 3a 3b	Ex 2b 3a 3b	BILAN
MONDAY: Remote group WORKSHOP by videoconference	TUESDAY : Remote group WORKSHOP by videoconference	MONDAY: Remote group WORKSHOP by videoconference	TUESDAY : Remote group WORKSHOP by videoconference	MONDAY: Remote group WORKSHOP by videoconference
INDIVIDUAL DAILY TRAINING with videos	INDIVIDUAL DAILY TRAINING with videos	INDIVIDUAL DAILY TRAINING with videos	INDIVIDUAL DAILY TRAINING with videos	INDIVIDUAL DAILY TRAINING with videos

INCLUSION	WEEK 1	WEEK 2	WEEK 3	WEEK 4	WEEK 5
Daily collective WORKSHOPS	MONDAY	TUESDAY	MONDAY	TUESDAY	MONDAY
	Orthophonie 1	Harmonie 1	Orthophonie 2	Harmonie 2	Orthophonie 3
	Ex 1a 1b 2a	Ex 1a 1b 2a	Ex 2b 3a 3b	Ex 2b 3a 3b	BILAN
Daily individual EXERCICES	Tue. V1a	Mon. V ortho1	Tue. V2b	Mon. V ortho 2	Tue. V ortho 1
	Wed. V1b	Wed. V 1a	Wed. V3a	Wed. V2b	Wed. V harm 1
	Thu. V2a	Thu. V 1b	Thu. V3b	Thu. V3a	Thu. V ortho 2
exercices and/or workshops VIDEOS	Wen. V1a	Wen. V 2a	Wen. V2b	Wen. V3b	Wen. V harm 2
	Sat. V1b	Sat. V ortho1	Sat. V3a	Sat. V ortho 2	Sat. V Bilan
	Sun. V2a	Sun. V harm1	Sun. V3b	Sun. V harm 2	Sun. V Bilan

Throughout the protocol, patients will be asked to complete the Tinnitus-VAS G and associated questionnaires as outlined in Section 5.2.1.2 (at visits T0, T1, and T2).

5.2.4.2. RAPP Group Workshops

These collective workshops to support hearing and communication disorders, set up remotely by the MUS'E association during the March 2020 lockdown, have been integrated into the care pathway offered by the Orléans Tinnitus Multidisciplinary Team since September 2021.

They offer exercises developed by the principal investigator in musicophonology and tinnitus promoting somatosensory listening, vocal expressiveness and the sharing of each participant's bodily and feelings.

The regular workshop lasts from 1h30 to 2h (from 8pm) and is carried out in small groups of 4 to 12 participants maximum. This group workshop is open to all people who need to be listened to, more particularly patients in need of care for:

- Disabling tinnitus, hyperacusis, misophonia, deafness, cochlear implants,
- Speech disorders, aphasia, dysarthria, dysphonia...

Its general objectives are to promote the opening of emotional and bodily listening, to tame tinnitus and adjust the relationship to one's environment.

During the protocol, this workshop will be composed of 6 to 10 tinnitus patients from the study and MUS'E resource patients or professionals (e.g. speech therapists). Patients will participate in 5 group workshops by

videoconference via a Jitsi link, secured "MUS'E A DISTANCE" (appendix 11). They will be recorded with the agreement of all participants (protocol patients, professionals and patient resources) who will decide to leave their camera and microphone open or closed to maintain their anonymity. These videos will then be sent to the patient each weekend, to allow him or her to benefit again from the group dynamic, experienced at the time of the group session.

5.2.4.3. Independent RAPP sessions

The principal investigator pre-recorded 6 videos of 30 minutes each, describing in detail each of the 6 RAPP exercises, thus allowing the patient to practice between 2 group workshops.

The patient will view, every evening around 8 p.m., a different video for which he will receive the internet link to download, every day of the week except the day of the collective workshop. During the 30 minutes of the video, he will perform the proposed exercises that he will do a second time independently for about 30 minutes, i.e. a training session of about an hour every evening of the week, outside of group sessions.

He will note on the Siopi application, 10 minutes after training, the tinnitus VAS-G and the 5 associated questions.
During the next group workshop, the investigator will check the quality of each patient's investment, by questioning them and observing the evolution of their ability to understand and execute the exercises.

5.2.4.4. Standardisation

After the clinical study, our six daily training videos will be open source to standardise RAPP exercises in clear progressive steps:

- Proprioception exercises 1a and 1b,
- Sound exercises 2a and 2b,
- Residual inhibition exercises 3a and 3b.

Despite careful planning, standardising five consecutive weekly group sound mediation workshops is challenging. Variations in group dynamics, stemming from the therapists' and patients' personalities, attitudes, and engagement, significantly affect each session, regardless of hearing impairment.

5.2.4.5. Attendance

Fidelity to individual and group training is tracked after completion through two methods: patients submit a follow-up sheet during their consultation, and all protocol participants complete an online questionnaire detailing the number of views, exercises practiced from six training videos, experiences during five group sessions, and key takeaways from each session video.

Regarding the strategy for handling missing data, participants will receive verbal reminders during study visits and interactions only. More intensive reminders were deliberately avoided, as repeatedly prompting participants to assess their tinnitus may increase their focus on it and interfere with the habituation process (which is a key objective of the intervention).

5.2.5. EEG HD

The EEG-HD sessions are identical for all patients in the study. The actual experimental protocol takes place in a room dedicated to the HD EEG (EGI system, 256 channels). The examination will be carried out in the neurology department of the Orléans University Hospital at T0 and T1 under the responsibility of the 2 associated investigating neurologists, by a technician who will install an HD EEG headset on the patient's head.

The measurement is carried out in a seated position, 256 electrodes distributed over the entire cranial cavity that record the brain connectivity at rest (total of 10 min):

- With your eyes closed for 5 minutes, with the instruction to "think of nothing" before the computerized craving induction task,
- Eyes closed, 5 minutes, with the instruction to "think of nothing" after the computerized craving induction task.

For the patient, it will take 15 minutes considering the time it takes to put on and take off the helmet.

5.2.5.1. Description of the strategy/procedure for acquiring the HD EEG

The electrical activity of the brain will be collected by the 256-electrode HD EEG-system (EGI Electrical Geodesics Inc. System, Figure 3) placed according to the 10-20 and 10-5 systems with an acquisition frequency of 1000 Hz. The International System 10-20 recommends a standardized arrangement of frequently used electrodes.

Each electrode is identified by a letter encoding its position in relation to the major brain regions and a number that defines the hemisphere (e.g. F1 for left frontal region). System 10-20 only takes into account a small number of electrodes. The position of additional electrodes (such as EEG-HD) is usually defined in relation to the positions of the electrodes belonging to this system (so-called system 10-5).

The 256 electrodes are already placed on a cap, the placement of the electrodes will take only 5 minutes. Before this placement, the participant's head circumference will have been measured to choose the most suitable cap, three cup sizes being available (S, M, L).

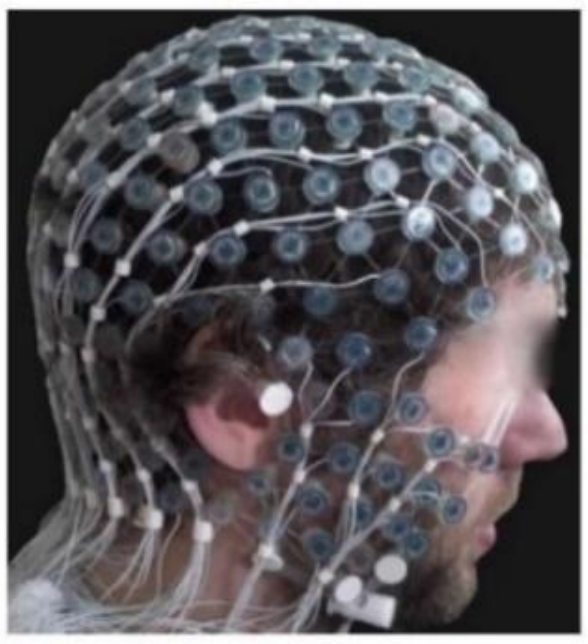

Figure 3 : HD EEG Headset

The data collected will be recorded live on the EEG station (Mac, NetStation software - EGI) in the same file (the management of this data is described in section 7).

The various brain regions of interest will be studied, in order of priority:

- L'anterior cingulate cortex (pregenual et dorsal)
- The auditory cortices
- The precuneus
- The parahippocampe
- The tonsils and insula, potentially the nucleus acumbens

5.3. CONDUCT OF THE STUDY

5.3.1. Inclusion procedures

➢ **Pre-selection visit**

Patients will be informed about the study via the different modalities presented in section 4.4. A pre-selection visit may be carried out at the Orléans University Hospital or by teleconsultation with an investigator as the various applications are collected through:

- Phoniatric consultations or the Tinnitus Multidisciplinary Team
- From the Siopi application or other associative networks (MUS'E, France Acouphènes)

This pre-selection visit will make it possible to:

- Review the eligibility criteria in part via the questionnaires on Siopi
- Explain the protocol to the patient and deliver the newsletter in person or by email
- Make an appointment for the inclusion visit at the Orléans University Hospital if the patient wishes to participate in the study.

➢ **Inclusion visits (= T0)**

The inclusion visit will be carried out at the ORLEANS University Hospital with a study investigator. The study will be presented to the patient and if the patient agrees to participate, the informed consent form will be signed (2 original copies) and the visit will include:

In the ENT department:

1- **Medical examination in acouphenology (60 mins):**
    - Compendium of inclusion and non-inclusion criteria (sections 4.2 and 4.3)
    - Completion of self-assessment questionnaires with the investigator:
        - THI paper questionnaire (Annex 3)
        - Tinnitus VAS-G and the 5 associated scales (2-noise, 3-cervical, 5-jaw, 7-sleep, 11-stress (Appendix 4)
        - Degree of insomnia (Appendix 5)
    - Laryngeal function assessment with the GRBAS-I scale and the VHI-10 questionnaire (section 5.2.1.3; appendices 6 and 7)
    - RAPP test with spectrogram feedback (section 5.2.3)
    - List of therapies that have been implemented on a regular basis for more than 2 months

2- **Audiometric tests (15 mins) (section 5.2.2)**

Following the performance of these various examinations, if the patient meets the eligibility criteria, he or she will be randomized to the study in either Arm A or Arm B.

In the neurology department:

1- **EEG HD (15 mn) (section 5.2.5)**

It is not mandatory to perform all these examinations on the same day (ENT and neurological examinations). Inclusion visit reviews must be performed within 30 days prior to D1 of the study and after signing consent (study-specific reviews). HD EEG can be performed after the patient has been randomized to the study (no impact on eligibility criteria).

5.3.2. Patient follow-up

As part of this study, 2 visits will be carried out at T1 and T2 at the ORLEANS University Hospital.

- **T1 visit**

The T1 visit should be carried out within 15 days of the end of RAPP training for arm A, on day 42 (+/- 7 days) for patients in both arms.

For this visit, the following examinations will be carried out:

- Medical visit (20 mins), completion of self-assessment questionnaires with the investigator:
  - Completion of the THI questionnaire
  - VAS-G tinnitus and the 5 associated scales
- HD EEG in neurology (15 mins): 5 mins setup + 10 mins recording

- **T2 visit**

The T2 visit should be carried out within 15 days of the end of RAPP training for arm B, on day 92 (+/- 7 days) for patients in both arms.

For this visit, the following examinations will be carried out:

- Medical visit (20 mins), completion of self-assessment questionnaires with the investigator:
  - Realization of the paper THI questionnaire
  - VAS-G tinnitus and the 5 associated scales

- **RAPP Training Protocol (Phase 1 Arm A, Phase 2 Arm B):**

After randomization in the study, patients will follow the following training protocol:

Phase 1 of the study schedule

The start of the experiment will begin after randomization when the number of patients is sufficient to constitute groups of 6 to 8 patients in the study (see maximum time in section 5.3.1). For patients in Arm A, the training lasts 5 weeks and will be performed according to the information provided in Section 5.2.4 (RAPP training) and Section 5.2.1.2 (VAS-G tinnitus and associated Siopi application questions). During this phase 1, patients in Arm B do not do any RAPP training, only EVA-G and associated questions on the Siopi application (see section 5.2.1.2).

Phase 2 of the study schedule

After the 1st assessment (T1), patients in Arm B will train for 5 weeks between T1 and T2, according to the information provided in Section 5.2.4 (RAPP training) and Section 5.2.1.1 (VAS-G and associated Siopi application questions). During this phase 2, patients in Arm A do not do any RAPP training, only EVA-G and associated questions on the Siopi application (see section 5.2.1.2).

Regardless of the period, with or without RAPP training, patients in both groups will be able to benefit from the current therapies they have already been engaged in for more than 2 months. During medical visits at T0, T1 and T2, the investigator will note the presence or not.

The reviews to be conducted are summarized in the study schedule in the next section.

5.3.3. Study timeline

Actions	Pre-selection visit	T0 (Inclusion visit)	Phase 1	T1 J42 (+/- 7 days)	Phase 2	T2 J92 (+/- 7 days)	Commentaires
Information patient et pré-screening	X						Teleconsultation or ENT service
Informed consent		X					
Medical examination with clinical examination		X		X		X	
Demographics		X					
Recording on the Siopi app		X					Refer to section 5.2.1.1
Vocal assessment (GRBAS-I and VHI10)		X					Refer to section 5.2.1.3
Insomnia Severity Index Questionnaire		X					Refer to section 5.2.1.2 (sur l'application Siopi)
Audiometric test (tonal and vocal audiogram and acouphenometry)		X					Refer to section 5.2.2
RAPP Testing		X					Refer to section 5.2.3
Verification of eligibility criteria		X					If a patient does not meet the criteria, they will not be randomized to the study
Randomization		X					
Tinnitus Handicap Index		X*		X*		X*	Refer to section 5.2.1.2<br>* On the Siopi application in the presence of the investigator
Tinnitus EVA-G Scale and 5 Related Questions		X*	X**	X*	X**	X*	* On the Siopi application in the presence of the investigator<br>** On the Siopi application independently, 4 times a week<br>Refer to section 5.2.1.2
HD- EEG		X		X			Refer to section 5.2.5.
RAPP training			Group A		Group B		Training over 5 consecutive weeks<br>Refer to section 5.2.4.

5.4. DURATION OF THE STUDY

5.4.1. Start of the study

The start of the study will be defined as the date of signing the consent form of the first patient.

5.4.2. Study schedule

- The duration of the inclusion period is 36 months.
- The follow-up period for each subject is 5 to 6 months.
- The total duration of the study is estimated at 42 months.

The 56 patients to be included in the study will be divided into different training sessions (each session has 6 to 10 patients from the study), scheduled as patients are recruited and according to their randomization group.

5.4.3. End of Study

The end of the study is defined as the date of the last patient's last visit.

5.5. DESCRIPTION OF PERMANENT OR TEMPORARY SHUTDOWN RULES

5.5.1. Termination of a person's participation in research

Patients may be discontinued from the study at any time for the following reasons:

- Withdrawal of consent: Patients will be able to withdraw their consent and request to leave the study at any time and for any reason. In the event of premature discharge, the investigator should document the reasons as fully as possible.
- Serious Adverse Event / Investigator's Decision: The investigator may temporarily or permanently discontinue a subject's participation in the study for any reason that would be in the best interests of the patient, particularly in the case of serious adverse events. In the event of an adverse event, serious or not, precise follow-up may be considered depending on the severity of the adverse event and the severity.
- Death
- Lost to follow-up: In the event of a subject who has been lost to follow-up, the investigator will make every effort to re-establish contact with the person.
- A patient's discharge from study will not change his or her usual management in relation to his or her symptoms; however, he will not have the specific examinations of the study (THI and EVA-G with the investigator, EEG HD).

In the event of discontinuation of the study-specific procedures (e.g. the patient's refusal to do the HD EEGs, etc.), the patient will continue to participate in the study unless the patient refuses (withdrawal of consent). Data will continue to be collected until the end of the study.

5.5.2. Stopping part or all the research

The study may be terminated prematurely if unexpected, serious adverse events occur requiring a review of the strategy profile. Similarly, unforeseen events or new product information, which are unlikely to achieve the objectives of the study or clinical programme, may cause the sponsor to terminate the study prematurely. The ORLEANS University Hospital reserves the right to interrupt the study at any time if it is found that the inclusion objectives are not achieved.

6. SAFETY ASSESSMENT

As this is a category 2 study, no vigilance related to the research protocol will be put in place.
However, the monitoring and reporting of incidents and risks of incidents resulting from participation in the study will be reported via the usual vigilance declaration circuit of the ORLEANS University Hospital.

7. RIGHT OF ACCESS TO DATA AND SOURCE DOCUMENT

7.1. ACCESS TO DATA

In accordance with GCP (Good Clinical Practice)
- the sponsor is responsible for obtaining the agreement of all parties involved in the research to ensure direct access to all research locations, source data, source documents and reports for the purpose of quality control and audit by the sponsor,
- the investigators will make available to the persons responsible for monitoring, quality control or auditing biomedical research, the documents and individual data strictly necessary for this control, in accordance with the legislative and regulatory provisions in force (Articles L.1121-3 and R.5121-13 of the Public Health Code).

7.2. SOURCES DOCUMENTS

Source documents are defined as any original document or object that proves the existence or accuracy of a data or fact, recorded during the clinical study and will be kept in accordance with the regulations in force by the investigator or by the hospital in the case of a hospital medical record. All data related to the study will be collected in the patient's medical record and on the Siopi app, including data:

- Medical file of the ENT department:
  - Audiometric tests at T0
  - The THI and VAS-G questionnaire completed with the investigator (T0, T1 and T2)
  - Data from the various tests (VHI-10, GRBAS-I, etc.) performed with the investigator as well as clinical, demographic and epidemiological data (T0)
  - The type of tinnitus and the date of onset
  - Wearing a hearing aid with sound therapy
  - Therapies that have been in place for at least 2 months and that they will be able to continue in the same way throughout the protocol, during and outside of RAPP training

This data may have already been noted by the patient on the other questionnaires in the Siopi application.

- Medical file of the neurology department:

    - The tracings of the HD EEG examinations at T0 and T1
    - The analysis reports of the HD EEG examinations at T0 and T1
- Longitudinal tracking log on the Siopi app:
    - All data from the THI and VAS-G questionnaires performed (T0, phase 1, T1, phase 2, T2)

  Insomnia Index

7.3. DATA TO BE COLLECTED DIRECTLY IN THE CASE REPORT FORM AND WHICH WILL BE CONSIDERED SOURCE DATA

All data will be available in the patient's source file or in the Siopi app.

7.4. CASE REPORT FORM

All information required by the protocol should be recorded in the case report form (CRF). Data should be collected as they are obtained and recorded in this CRF explicitly. Each missing data will have to be coded.
This electronic observation notebook will be set up thanks to an Internet data collection medium. A guidance document for the use of this tool will be provided to investigators.
Filling in the case report form via the internet by the competent people (investigators, clinical research staff, etc.) allows the Clinical Research Associate (CRA) to quickly and remotely view the data. The investigator is responsible for the accuracy, quality, and relevance of all data entered. In addition, when they are entered, this data is immediately checked for consistency. As such, it must validate any change in value in the CRF. These changes are subject to an audit trail. A justification may be included in the commentary. A paper printout may be requested at the end of the study, authenticated (dated and signed) by the investigator. A copy of this authenticated document for the sponsor must be archived by the investigator.

7.5. DATA ENTRY AND GENERATION

The data will be entered into a secure database developed by the ORLEANS University Hospital using Ennov Clinical® software. The data will be protected by a password and processed in accordance with the MR001 of the CNIL. Backups of this data will be made every day. The data will be entered by the competent people in each center (investigators, clinical research staff, etc.) who will have a personal and secure password to access the database.
The ARC in charge of the study at the sponsor's level will be trained in the use of the eCRF, then will oversee training the people of the research team who will carry out the entry. Partial exports of the database will be carried out regularly to ensure the follow-up of the study (monitoring).

7.6. DATA PRIVACY

In accordance with the provisions concerning the confidentiality of data to which persons responsible for quality control of biomedical research have access (Article L.1121-3 of the Public Health Code), in accordance with the provisions relating to the confidentiality of information concerning, in particular, the nature of the products, the tests, the persons who lend themselves to them and the results obtained (Article R. 5121-13 of the Public Health Code), Persons with direct access shall take all necessary precautions to ensure the

confidentiality of information relating to products, tests, persons taking part in them, and in particular as regards their identity and the results obtained.
These persons, in the same way as the investigators themselves, are subject to professional secrecy (under the conditions defined by Articles 226-13 and 226-14 of the Criminal Code).
During the research or at the end of the research, the data collected on the people who take part in it and transmitted to the sponsor by the investigators (or any other specialized stakeholders) will be pseudonymized.
They must not under any circumstances display the names of the persons concerned or their addresses in plain text.
For the registration of participants in the study, they will be identified via a coded number specific to the study (format: XX-XX, the first 2 digits correspond to the center and the next 2 correspond to the order of inclusion). The first letter of the participant's last name and the first letter of his or her first name will be recorded in the CRF for traceability during monitoring.
The sponsor will ensure that each person who participates in the research has given his or her consent for access to the individual data concerning him or her that is strictly necessary for the quality control of the research.

7.6.1. HD EEG Data Management

The data generated during the EEG-HD recordings in electronic format will be directly pseudonymized by the recording system, stored securely at the Orléans University Hospital and accessible only to persons authorized to use them (principal investigator of the center and any person delegated by the principal investigator).
EEG-HD data will be stored and transferred—using only patient codes specific to the study—to MINDIG via a secure platform, which includes a password-protected account. All data will remain on a secure MINDIG server throughout the period of statistical analysis. Once this analysis is complete, both raw and processed data will be securely sent to the sponsor, deleted from the MINDIG server, and archived at Orléans University Hospital on its secure server. Participants will be notified about whether their consent was specifically obtained regarding possible future reuse of their data for other research purposes, pending the necessary approvals from authorities and the participant.

HD-EEG data will be analysed primarily using MNE-Python, an open-source Python toolbox for electrophysiological data analysis, together with standard scientific Python libraries, including NumPy, SciPy, Pandas, scikit-learn, and Network X. The preprocessing pipeline will include data import, channel inspection, band-pass filtering, notch filtering, bad-channel detection and interpolation, re-referencing, segmentation of resting-state recordings, and artefact correction. Ocular, muscular, and cardiac artefacts will be identified using a combination of automated procedures, visual inspection, and independent component analysis implemented in MNE-Python. For each participant, quality-control metrics will be documented, including the number of rejected and interpolated channels, rejected epochs, and retained clean data duration.

Following preprocessing, cortical source activity will be reconstructed using MNE-Python inverse modelling tools. In the absence of individual MRI data, source reconstruction will be performed using a standard anatomical template adapted to the 256-channel HD-EEG montage. Source-level time series will be extracted from anatomically defined regions of interest, with particular focus on tinnitus-related networks, including auditory cortices, temporal and parietal regions, posterior cingulate cortex, precuneus, parahippocampal regions, and anterior cingulate cortex.

The primary HD-EEG outcome will be the change from T0 to T1 in source-level resting-state functional connectivity within tinnitus-related networks, comparing the immediate-training group with the delayed-training control group. Connectivity will be estimated in canonical frequency bands, with particular emphasis on the alpha band. Secondary exploratory EEG outcomes will include spectral power changes and dynamic microstate parameters, including microstate duration, occurrence, coverage, and transition probabilities. Associations between EEG changes and clinical improvement will be explored by correlating EEG-derived changes with changes in Tinnitus Handicap Inventory scores.

7.6.2. SIOPI application data management

Data on the SIOPI platform is managed and stored by the system of record. Patient information will be pseudonymized using a study-specific page, where each participant enters their patient number in PHONACOU. Study data is stored on SIOPI, and at the study's conclusion, only coded data will be delivered in person to the biostatistician at Orléans University Hospital.

7.6.3 Data Management and Quality Control

Prospective data will be gathered through an electronic data entry system (eCRF, Siopi). To make sure the information is consistent and reliable, a quality control plan has been established. This includes automatic validation checks when entering data (such as acceptable value ranges, required fields, and duplicate detection), independent review by a designated data manager, as well as regular monitoring during recruitment. Any errors or missing data will be examined, documented, and tracked. The information will be kept on a secure server that meets current regulations (like GDPR) and only authorized staff will have access. A comprehensive Data Management Plan (DMP) can be provided if needed.

8. STATISTIQUES

Les analyses statistiques seront réalisées par la biostatisticienne du CHU d'Orléans en charge de l'étude.

8.1. POPULATION ET STRATEGIE D'ANALYSES

8.1.1. Analysis population

The analysis will be performed in modified intent-to-treat, all subjects included in the study and who have completed the THI score at T0 and T1 will be considered in the primary outcome analysis (whether they have followed the study protocol correctly).
An intra-protocol analysis will also be conducted for the primary outcome, especially if we observe that a significant number of patients did not follow the study procedure correctly.
This will target patients with the THI score completed at T0 and T1 and who have correctly followed the protocol between T0 and T1 (i.e. who have done their 5 weeks of RAPP training for group A; and without RAPP training for group B).

8.1.2. Analytics strategy

8.1.2.1. Missing data management

It is assumed that the missing data will be randomly distributed (Missing At Random) and the analysis will be based on available data.
However, if the rate of missing data exceeds 10% (for the primary endpoint and the VAS-G tinnitus scales between T0 and T1), analyses without and with imputation of data extrapolated from the distribution will be performed.

8.1.2.2. Risk of the first kind

The risk of the first species used for comparisons is 5% (bilateral).

8.1.2.3. Moment of analysis

The analysis of all criteria will be carried out at the end of the study.

8.2. PLANNED STATISTICAL METHODS

8.2.1. Descriptive methods

In general, quantitative variables will be described by their mean, standard deviation, median and interquartiles; Qualitative variables will be described in terms of numbers and percentages.

All patients included in the study will be described according to their characteristics (demographic, clinical, etc.) at baseline, overall and according to the randomization group.

As much as possible, we will try to associate a graphical representation with the analyses.

8.2.2. Comparative methods

Quantitative variables will be compared between groups (A and B), by the student's test if the test validity conditions are met, or by a student's test for unequal variances, or by a nonparametric Wilcoxon/MannWithney test. A linear regression model will be used adjust for potential confounding variables (the model assumptions will be verified).

Longitudinal data will be analysed using mixed-effects model including fixed effects and a random intercept for participants.

Within-group comparisons will be performed using paired Student's t-test (or signed Wilcoxon test if the conditions of the student test are not true). Correlations will be assessed using Pearson's correlation coefficient.

The primary outcome is the only confirmatory hypothesis test. Secondary outcome analyses are considered exploratory. When multiple comparisons are performed within the same family of secondary outcomes (e.g. VAS scales), p-values will be adjusted using the Holm-Bonferroni method. For mixed-effects models, post hoc

pairwise comparisons, when applicable, will be adjusted for multiple testing using the procedure implemented in the statistical software.

8.2.3. Calculation agreement (if applicable)

The EVA scales are completed 4 times a week:

- During RAPP training: morning and evening, on the day of group workshops (Monday or Tuesday) and on Friday.
- During the weeks of control (without RAPP training) morning and evening, every Monday and Friday of the week.

Two calculation conventions will be used to meet the 1st secondary outcome:

- 5-week average of the daily differences in each VAS-G scale, for each group: Sum [(VAS-G measured in the morning –VAS-G measured in the evening) over 5 weeks] / 5*2 (the number of measurements).
- Daily average of each VAS, for each group: (morning VAS-G + evening VAS-G) /2.

8.2.4. Primary Purpose Analysis

The THI constitutes the primary outcome.
To compare the change in THI score at T1 between the 2 groups, the appropriate test described in section 8.2.2 will be used. A regression model may subsequently be used, to adjust for confounding variables such as the patients' "background treatment", the RAAP test score, ...
A graphical representation of THI scores for both groups will be provided using box plots.

8.2.5. Analysis of secondary objectives

Unless otherwise specified, all statistical analyses described below will be performed using the methods detailed in Section 8.2.2. When applicable, the calculation conventions described in Section 8.2.3 will be used.

1. The EVA G tinnitus scales will be compared between the 2 groups (between T0 and T1) using the calculation conventions described in Section 8.2.3.

2. The EVA G tinnitus scales of group B will also be described between T1 and T2 (during the RAPP training period) to assess whether these findings support those observed in group A between T0 and T1.

3. The stability over time of the EVA G tinnitus scales and the THI score will be studied only in group A between T1 and T2 (post-training period). We will therefore compare the THI score at T2 to the THI score at T1. Similarly, for the EVA G tinnitus scale, the estimated average on the last day will be compared with that on the first day of this period.

4. The evolution of the THI score over time (T0, T1 and T2) will be described via a mixed model for each of the groups. An interaction between time and group will also be investigated. Adjustment variables may be included in the model if necessary.

5. The VAS G scales for 5 associated questions (noise, cervical, jaw, sleep, stress-anxiety) of the 2 groups, will be analysed between T0 and T1, using calculation convention (b) specified in Section 8.2.3.

8.2.6. Exploratory analyses in HD-EEG

- **EEG-HD experimental data :**

These data will be analyzed using classical (spectral analysis) and advanced (static and dynamic functional connectivity) signal processing methods, according to the following five steps: data preprocessing, cortical activity reconstruction, descriptor extraction, classification and outcome evaluation.
After cleaning the data (elimination of overly noisy segments and interpolation of the "bad" electrodes), using an automatic preprocessing algorithm called automagic (Pedroni et al, 2019), the networks will be represented by means of graphs, composed of nodes (representing brain regions), and links between nodes denoting functional connectivity between regions (Hassan & Wendling, 2018). The strength of the connection between the nodes will be defined by the strength of the statistical coupling, determined by the calculation of the phase synchronization (PLV and wPLI). To compare the two groups, two main analyses will be carried out on the resulting networks. The first analysis, called static, consists of comparing the global networks between the two groups. This is an essential step before addressing the differences in network dynamics. The second family of analyses, called dynamics, aims to explore the differences between groups in terms of the spatiotemporal dynamics of brain networks (or microstates). The two approaches are detailed below:

- **Statics (connectivity parameters):** Here, we will focus on the global and nodal characteristics of the network (segregation, integration and hubness; see Hassan et al, 2017) and at the level of connections (connectivity values between different regions), depending on the frequency band. The goal is to detect nodes and connections that are significantly more important than what might be observed by chance. All these statistics can then be compared between groups. Corrections for multiple comparisons will be made by the Bonferroni method. Finally, analyses estimating the links between electrophysiological variables and THI will be performed. The main endpoint will be the difference in terms of graphic characteristics between the groups. The quantification of the networks will be analysed in the light of the tinnitus scores used in a standard way.

- **Dynamics (microstate):** The objective here is to compare the dynamics of brain networks between different groups and conditions. We will use methods that can identify "functional connectivity states" (the few functional networks that summarize brain functions during a given task or rest). The dynamic reconfiguration of brain networks will be quantified using mainly methods based on source separation, k-means (so-called microstates) and modularity-based analysis. Measures such as fractional occupancy and double time (quantifying the percentage of brain networks present during the task) will be used to statistically compare groups (Tabbal 2020, Kabbara 2019).

We will make sure beforehand that the different criteria are homogeneous between the groups at T1.

8.2.7. Software used for analysis

Statistics (other than EEG) will be performed with SAS EG 8.3 or RStudio software.

8.3. ANTICIPATED NUMBER OF INDIVIDUALS TO BE INCLUDED IN THE RESEARCH

The study by Smeele et al.,2023, randomized, double-blind, on 20 patients sought to evaluate the impact of "high definition infralent pink noise" stimulation on the quality of life of patients with chronic tinnitus using scores such as the TFI (Tinnitus Functional Index), the mTQ (mini-Tinnitus questionnaire), and TCS (Tinnitus Catastrophozing Scale).
A second study conducted by Yadollahpour, A.et al., 2024, randomized, double-blind on 48 patients with refractory chronic tinnitus looked at the impact of transcranial stimulation (on the THI score. It demonstrated a significant difference of 20 points (standard deviation of 15) after the intervention between the group that received the stimulations and the placebo group.

According to Zeeman et al. (2011), the minimum clinically important difference (MCID) for the Tinnitus Handicap Inventory (THI) is 7, with a standard deviation of approximately 14. As no prior research has evaluated the impact of RAPP training on the THI, we hypothesize that participants who complete the 5-week RAPP training will exhibit a reduction in THI scores between T0 and T1 that is 12 points greater than that observed in the control group. This anticipated between-group difference surpasses the established MCID and represents a clinically meaningful yet conservative treatment effect relative to other non-invasive interventions. Assuming a standard deviation of 14, a two-sided alpha of 5%, and a statistical power of 80%, we have determined that 22 patients per group are required. To account for an estimated 20% of "non-evaluable" subjects—those lacking THI scores at T0 and T1—a total sample size of 56 patients will be included in the study.

8.4. STATISTICAL CRITERIA FOR STOPPING THE SEARCH

NA

9. Quality Control and Assurance

9.1. Access to data

A Clinical Research Associate (CRA) mandated by the sponsor will ensure the proper performance of the study, the collection of the data generated, their documentation, recording and reporting, in accordance with the Standard Operating Procedures implemented within the ORLEANS University Hospital and in accordance with Good Clinical Practices as well as the legislative and regulatory provisions in force.
The investigator and the members of his team agree to make themselves available during the Quality Control visits carried out at regular intervals by the Clinical Research Associate. During these visits, the following elements will be reviewed:

- Express consent and traceability of the consent in the medical file
- Compliance with the study protocol and the procedures defined therein
- Quality of the data collected in the case report form: accuracy, missing data, consistency of the data with the "source" documents (medical records, appointment books, originals of laboratory results, etc.)

A monitoring grade is developed prior to the implementation of the protocol, in order to define the quality control procedures and the frequency of these controls.
After each monitoring visit, the ARC will write a monitoring report, validated by the sponsor, and followed by a post-visit email sent to the centre (listing the details of its activity, the deviations noted and the corrective actions to be implemented).

On the other hand, the investigators undertake to accept the quality assurance audits carried out by the sponsor as well as the inspections carried out by the Competent Authorities. All data, documents and reports can be subject to regulatory audits and inspections without medical confidentiality being invoked.

10. Administrative and regulatory aspects

10.1. Initial notice

The protocol, the study information form and all the documents intended for patients will be submitted for opinion to a Committee for the Protection of Persons appointed by lot.
The notification of the CPP's favourable opinion will be forwarded to the study sponsor and the Competent Authority.

10.2. Substantial changes

In the event of a substantial change to the protocol by the investigator, it will be approved by the sponsor. The latter will have to obtain a favourable opinion from the CPP prior to its implementation. New consent from the people participating in the research will be collected if necessary.

10.3. Patient information and express consent

Patients will be fully and informed, in understandable terms, of the objectives and constraints of the study, of any risks involved, of the necessary monitoring and safety measures, of their rights to refuse to participate in the study or of the possibility of withdrawing at any time.
All this information is included in an information form given to the patient. The patient's written consent will be obtained by the investigator, or a physician representing the investigator prior to any study-specific procedures.

10.4. Indemnification of subjects

Patients will return to the hospital for HD EEG (if not on the same day as the ENT visits) and at the T1 and T2 visits, transport costs will be reimbursed.

10.5. Registration in the national register of persons suitable for biomedical research

Not applicable.

10.6. CNIL

This study falls within the framework of the "Reference Methodology" (MR-001) pursuant to deliberation no. 2018-153 of 3 May 2018 approving a reference methodology relating to the processing of personal data implemented in the context of research in the field of health with the consent of the data subject and repealing deliberation no. 2016-262 of 21 July 2016.
The ORLEANS University Hospital, sponsor of the study, signed a commitment to comply with this "Reference Methodology" on March 10, 2017.
It is also part of the General Data Protection Regulation (GDPR), which came into force on 25 May 2018.
Patients are informed via the newsletter of the identity of the person responsible for data processing, the data protection officer of the University Hospital, their right to lodge a complaint with the CNIL, a right of access and rectification; a right to portability, a right to erasure and a right to limit the processing of their personal data.

10.7. Archiving

The study documents must be kept in an active database (intermediate archiving) until 2 years after the last publication of the research results or, in the event of no publication, until the signature of the final report (in accordance with the MR-001 reference methodology).
They will then be archived in accordance with the regulations in force in the dedicated premises of each investigator center and in those of the research department of the ORLEANS University Hospital.
No displacement or destruction may be carried out without the agreement of the sponsor. After the regulatory archiving period, the sponsor will be consulted for destruction. All data, documents and reports may be subject to audit or inspection.

11. FINANCING AND INSURANCE

11.1. STUDY BUDGET

The costs related to this research will be covered by the ORLEANS University Hospital:

- Specific insurance
- Data management: research design, statistical analysis, data management, CRF
- Study management: project management, coordination and monitoring
- Quality control by a CRA mandated by the developer
- Management of training sessions by RAPP
- Training preparation by RAPP
- Consultation of T0, T1 and T2 visits (ENT department)
- Performing HD EEGs at visits T0 and T1 (neurology department)
- Siopi application (development of a specific page for the study)
- Patient travel expenses
- Presentation of results: publications, congresses
- Miscellaneous expenses: stationery, prepaid envelopes

11.2. INSURANCE

The ORLEANS University Hospital has taken out insurance with RELYENS MUTUAL INSURANCE for the entire duration of the study, guaranteeing its own civil liability as well as that of any party involved in the conduct of the study.

It will also ensure full compensation for the harmful consequences of the research for the person who participates in the research and his or her beneficiaries, unless he or she can prove that the damage is not attributable to his or her fault or that of any person involved, without it being possible to oppose the act of a third party or the voluntary withdrawal of the person who had initially consented to the research.

12. FEASIBILITY OF THE STUDY

The recruitment of the 56 tinnitus patients seems quite realistic in view of the large patient base followed by the 9 members of the Orléans Tinnitus Multidisciplinary Team (238 tinnitus patients listed on the secure Sphere Mediateam platform and more than a dozen new patients per month encountered in phoniatric consultations at the ENT department of the ORLEANS University Hospital).

Finally, the clinical study team will be reinforced by associate investigators, two neurologists from the ORLEANS University Hospital with extensive experience in clinical studies that make the newly acquired HD EEG platform available, as well as by two associate neuroscientists, the first creator of the MINDIG company bringing expertise in the analysis of connectivity in HD EEG, the second is a tinnitus specialist, creator of the Siopi application.

13. PUBLICATION RULES

The results of the study may be communicated via congresses or publications.

The first author will be the principal investigator of the study. The last author will be an investigator from the neurology department of the Orléans University Hospital.

The other positions will be assigned to each associate investigator according to the importance of their involvement with the patients included in the study and their expertise in “acouphonologie” and HD EEG.
The scientific communications and reports corresponding to this study will be carried out under the responsibility of the coordinating investigator of the study with the agreement of the investigators in charge. The co-authors of the report and publications will be the investigators and clinicians involved, in proportion to their contribution to the study, as well as the biostatistician and associate researchers.

The rules of publication will follow international recommendations (N Engl J Med, 1997; 336:309-315).

The study will be registered on an open-access website (Clinical trials) before the inclusion of the 1st patient in this study.

14. BIBLIOGRAPHY

- Aazh, H., Baguley, D. M., & Moore, B. C. J. (2019). Factors Related to Insomnia in Adult Patients with Tinnitus and/or Hyperacusis: An Exploratory Analysis. Journal of the American Academy of Audiology, *30*(09), 802809. https://doi.org/10.3766/jaaa.18020
- Aglieri, V., Chaminade, T., Takerkart, S., & Belin, P. (2018). Functional connectivity within the voice perception network and its behavioural relevance. NeuroImage, 183, 356365. https://doi.org/10.1016/j.neuroimage.2018.08.011
- Arns, M., De Ridder, S., Strehl, U., Breteler, M. and Coenen, A., 2009. Efficacy of neurofeedback treatment in ADHD: the effects on inattention, impulsivity and hyperactivity: a meta-analysis. Clinical EEG and neuroscience, 40(3), pp.180-189.
- Assouly, K. K. S., Dullaart, M. J., Stokroos, R. J., van Dijk, B., Stegeman, I., & Smit, A. L. (2021). Systematic Review on Intra- and Extracochlear Electrical Stimulation for Tinnitus. Brain sciences, 11(11), 1394. https://doi.org/10.3390/brainsci11111394
- Bankstahl, U., & Görtelmeyer, R. (2013). Measuring subjective complaints of attention and performance failures—Development and psychometric validation in tinnitus of the self-assessment scale APSA. Health and Quality of Life Outcomes, 11(1), 86. https://doi.org/10.1186/1477-7525-11-86
- Barack, D. L., & Platt, M. L. (2021). Neuronal Activity in the Posterior Cingulate Cortex Signals Environmental Information and Predicts Behavioral Variability during Trapline Foraging. The Journal of neuroscience : the official journal of the Society for Neuroscience, 41(12), 2703–2712. https://doi.org/10.1523/JNEUROSCI.0305-20.2020
- Borel, S., Gatignol, P., Gros, A., & Tran, T. M. (2022). Manuel de recherche en orthophonie. De Boeck Supérieur.
- Borel, S., & Leybaert, J. (2020). Surdités de l'enfant et de l'adulte : Bilans et interventions orthophoniques. De Boeck supérieur.
- Bowl, M. R., & Dawson, S. J. (2019). Age-Related Hearing Loss. Cold Spring Harbor Perspectives in Medicine, 9(8), a033217. https://doi.org/10.1101/cshperspect.a033217
- Brüggemann, P., Szczepek, A. J., Rose, M., McKenna, L., Olze, H., & Mazurek, B. (2016). Impact of Multiple Factors on the Degree of Tinnitus Distress. Frontiers in Human Neuroscience, 10. https://doi.org/10.3389/fnhum.2016.00341
- Cederroth, C. R., Gallus, S., Hall, D. A., Kleinjung, T., Langguth, B., Maruotti, A., Meyer, M., Norena, A., Probst, T., Pryss, R., Searchfield, G., Shekhawat, G., Spiliopoulou, M., Vanneste, S., & Schlee, W. (2019). Editorial: Towards an Understanding of Tinnitus Heterogeneity. Frontiers in Aging Neuroscience, 11, 53. https://doi.org/10.3389/fnagi.2019.00053
- Cheung, S., Henderson-Sabes, J., Mastick, J., Abrams, G., Snowberg, K., Alfaro, E., Quinn, M., Paul, S., Cooper, B., Wallhagen, M., Conley, Y., Levine, J., & Miaskowski, C. (2023). Cancer survivors and neurotoxic chemotherapy : Hearing loss and tinnitus. BMJ Supportive & Palliative Care, 13(3), 345353. https://doi.org/10.1136/spcare-2022-003684
- Courtis, S. (2017). Le sens vibratoire et les acouphènes, musique et hypnose. Revue française de musicothérapie. 36 (2).
- Dauman, N., & Keller, P.-H. (2010). Épistémologie de l'acouphène : Histoire d'une controverse scientifique : Cliniques méditerranéennes, n° 82(2), 317330. https://doi.org/10.3917/cm.082.0317
- DeJonckere, P. (2016). Le feedback instrumental en pédagogie du chant. In De la voix parlée au chant (Klein-Dallant).

- De Ridder, D., Vanneste, S., Weisz, N., Londero, A., Schlee, W., Elgoyhen, A. B., & Langguth, B. (2014). An integrative model of auditory phantom perception: tinnitus as a unified percept of interacting separable subnetworks. *Neuroscience and biobehavioral reviews*, *44*, 16–32. https://doi.org/10.1016/j.neubiorev.2013.03.021
- D. P., S., T., L., B., T., M. A., P., K., K., C. M., S., & G. D., S. (2015). Multisensory attention training for treatment of tinnitus. Scientific Reports, 5(1), 10802. https://doi.org/10.1038/srep10802
- Dode, A., Mehdi, M., Pryss, R., Schlee, W., Probst, T., Reichert, M., Hauck, F., & Winter, M. (2021). Using a visual analog scale (VAS) to measure tinnitus-related distress and loudness: Investigating correlations using the Mini-TQ results of participants from the TrackYourTinnitus platform. In Progress in Brain Research (Vol. 263, p. 171190). Elsevier. https://doi.org/10.1016/bs.pbr.2020.08.008
- Dupont, M., & Lejeune, B. (2021). Surdité et implant cochléaire : 625 exercices d'entraînement auditif (3e éd). Elsevier Masson.
- Engineer, N. D., Møller, A. R., & Kilgard, M. P. (2013). Directing neural plasticity to understand and treat tinnitus. Hearing Research, 295, 5866. https://doi.org/10.1016/j.heares.2012.10.001
- Ernst, E. (2016). La conservation de la voix et de la parole chez l'adulte devenu sourd ou malentendant. In De la voix parlée au chant. p 151 (Klein-Dallant).
- Esmaili, A. A., & Renton, J. (2018). A review of tinnitus. Australian Journal of General Practice, 47(4), 205208. https://doi.org/10.31128/AJGP-12-17-4420
- Figueiredo, R. R., De Azevedo, A. A., & De Mello Oliveira, P. (2009). Correlation analysis of the visual-analogue scale and the Tinnitus Handicap Inventory in tinnitus patients. Brazilian Journal of Otorhinolaryngology, 75(1), 7679. https://doi.org/10.1016/S1808-8694(15)30835-1
- Fournier, P., Cuvillier, A.-F., Gallego, S., Paolino, F., Paolino, M., Quemar, A., Londero, A., Norena, A. (2018). Une nouvelle méthode de masquage et d'inhibition résiduelle des acouphènes. Tendances de l'audition, 22, 233121651876999. https://doi:10.1177/2331216518769996
- Fuller, T., Cima, R., Langguth, B., Mazurek, B., Vlaeyen, J. W., & Hoare, D. J. (2020). Cognitive behavioural therapy for tinnitus. Cochrane Database of Systematic Reviews, 2020(1). https://doi.org/10.1002/14651858.CD012614.pub2
- Galazyuk, A. V., Voytenko, S. V. et Longenecker, R. J. (2016). Suppression avancée durable de la décharge spontanée dans les neurones auditifs : implication dans l'inhibition résiduelle des acouphènes. Journal of the Association for Research in Otolaryngology: JARO, 18(2), 343-353. https://doi:10.1007/ s10162-016-0601-9
- Ghulyan-Bédikian, V., Paolino, M., Giorgetti-D'Esclercs, F., & Paolino, F. (2010). Propriétés psychométriques d'une version française du Tinnitus Handicap Inventory. L'Encéphale, 36(5), 390396. https://doi.org/10.1016/j.encep.2009.12.007
- Guillard, R., Decobecq, F., Fraysse, M.-J., Favre, A., Congedo, M., Loche, V., Boyer, M., & Londero, A. (2023). Traduction française validée du questionnaire standardisé d'anamnèse d'acouphène ESIT-SQ. Annales françaises d'Oto-rhino-laryngologie et de Pathologie Cervico-faciale, 140(4), 155160. https://doi.org/10.1016/j.aforl.2022.09.015
- Guillard, R., Decobecq, F., Fraysse, M.-J., Favre, A., Congedo, M., Loche, V., Boyer, M., Londero, A., Traduction française validée du questionnaire standardisé d'anamnèse d'acouphène ESIT-SQ, Annales françaises d'Oto-rhino-laryngologie et de Pathologie Cervico-faciale, Volume 140, Issue 4, 2023, Pages 155-160, ISSN 1879-7261, https://doi.org/10.1016/j.aforl.2022.09.015
- Güntensperger, D., Kleinjung, T., Neff, P., Thüring, C., & Meyer, M. (2020). Combining neurofeedback with source estimation: Evaluation of an sLORETA neurofeedback protocol for chronic tinnitus treatment. Restorative neurology and neuroscience, 38(4), 283–299. https://doi.org/10.3233/RNN-200992
- Ghodratitoostani, I., Gonzatto, O. A., Jr, Vaziri, Z., Delbem, A. C. B., Makkiabadi, B., Datta, A., Thomas, C., Hyppolito, M. A., Santos, A. C. D., Louzada, F., & Leite, J. P. (2022). Dose-Response Transcranial Electrical

Stimulation Study Design: A Well-Controlled Adaptive Seamless Bayesian Method to Illuminate Negative Valence Role in Tinnitus Perception. Frontiers in human neuroscience, 16, 811550. https://doi.org/10.3389/fnhum.2022.811550

- Hassan, M., Wendling, F., 2018. Electroencephalography Source Connectivity: Aiming for High Resolution of Brain Networks in Time and Space. IEEE Signal Process. Mag. 35, 81–96. https://doi.org/10.1109/MSP.2017.2777518
- Henry, J. A., Reavis, K. M., Griest, S. E., Thielman, E. J., Theodoroff, S. M., Grush, L. D., & Carlson, K. F. (2020). Tinnitus : An epidemiologic perspective. Otolaryngologic Clinics of North America, 53(4), 481499. https://doi.org/10.1016/j.otc.2020.03.002
- Hu, H., Lin, X., Ye, Z., Fang, L., Gao, H., & Zhang, Q. (2024). Application of functional near-infrared spectroscopy (fNIRS) in tinnitus research: contemporary insights and perspectives. Frontiers in psychology, 15, 1334660. https://doi.org/10.3389/fpsyg.2024.1334660
- Huang, B., Wang, X., Wei, F., Sun, Q., Sun, J., Liang, Y., Chen, H., Zhuang, H., & Xiong, G. (2022). Notched Sound Alleviates Tinnitus by Reorganization Emotional Center. Frontiers in human neuroscience, 15, 762492. https://doi.org/10.3389/fnhum.2021.762492 Ioannidis, J. P. A. (2018). The Proposal to Lower P Value Thresholds to .005. JAMA, 319(14), 1429. https://doi.org/10.1001/jama.2018.1536
- Huang, H., Cai, Y., Feng, X., & Li, Y. (2021). An electroencephalogram-based study of resting-state spectrogram and attention in tinnitus patients] = Journal of biomedical engineering, 38(3), 492–497. https://doi.org/10.7507/1001-5515.202012015
- Jarach, C. M., Lugo, A., Scala, M., Van Den Brandt, P. A., Cederroth, C. R., Odone, A., Garavello, W., Schlee, W., Langguth, B., & Gallus, S. (2022). Global Prevalence and Incidence of Tinnitus: A Systematic Review and Meta-analysis. JAMA Neurology, 79(9), 888. https://doi.org/10.1001/jamaneurol.2022.2189
- Jaroszynski, C., Job, A., Jedynak, M., David, O., & Delon-Martin, C. (2022). Tinnitus Perception in Light of a Parietal Operculo-Insular Involvement: A Review. Brain sciences, 12(3), 334. https://doi.org/10.3390/brainsci12030334
- Jensen, M., Alanis, J. C. G., Hüttenrauch, E., Winther-Jensen, M., Chavanon, M. L., Andersson, G., & Weise, C. (2023). Does it matter what is trained? A randomized controlled trial evaluating the specificity of alpha/delta ratio neurofeedback in reducing tinnitus symptoms. Brain communications, 5(4), fcad185. https://doi.org/10.1093/braincomms/fcad185
- Jensen, M., Hüttenrauch, E., Schmidt, J., Andersson, G., Chavanon, M. L., & Weise, C. (2020). Neurofeedback for tinnitus: study protocol for a randomised controlled trial assessing the specificity of an alpha/delta neurofeedback training protocol in alleviating both sound perception and psychological distress in a cohort of chronic tinnitus sufferers. Trials, 21(1), 382. https://doi.org/10.1186/s13063-020-04309-y
- Jin, I.-K., Choi, S.-J., Yoo, J., Jeong, S., Heo, S., & Oh, H. (2021). Effects of Tinnitus Sound Therapy Determined Using Subjective Measurements. Journal of the American Academy of Audiology, 32(04), 212218. https://doi.org/10.1055/s-0041-1722984
- Job, A., Pons, Y., Lamalle, L., Jaillard, A., Buck, K., Segebarth, C., & Delon-Martin, C. (2012). Abnormal cortical sensorimotor activity during "Target" sound detection in subjects with acute acoustic trauma sequelae: an fMRI study. Brain and behavior, 2(2), 187–199. https://doi.org/10.1002/brb3.21
- Job, A., Jacob, R., Pons, Y., Raynal, M., Kossowski, M., Gauthier, J., Lombard, B., & Delon-Martin, C. (2016). Specific activation of operculum 3 (OP3) brain region during provoked tinnitus-related phantom auditory perceptions in humans. Brain structure & function, 221(2), 913–922. https://doi.org/10.1007/s00429-014-0944-0
- Kabbara, A., Khalil, M., O'Neill, G., Dujardin, K., El Traboulsi, Y., Wendling, F., Hassan, M., 2019. Detecting modular brain states in rest and task. Netw Neurosci. 3(3): 878–901.

- Krasny-Pacini, A., & Evans, J. (2018). Single-case experimental designs to assess intervention effectiveness in rehabilitation: A practical guide. Annals of Physical and Rehabilitation Medicine, 61(3), 164179. https://doi.org/10.1016/j.rehab.2017.12.002
- Kratochwill, T. R., Hitchcock, J., Horner, R. H., Levin, J. R., Odom, S. L., Rindskopf, D. M., & Shadish, W. R. (2010). Single case designs technical documentation. What Works Clearinghouse. http://ies.ed.gov/ncee/wwc/pdf/wwc_scd.pdf
- Lan, L., Li, J., Chen, Y., Chen, W., Li, W., Zhao, F., Chen, G., Liu, J., Chen, Y., Li, Y., Wang, C. D., Zheng, Y., & Cai, Y. (2021). Alterations of brain activity and functional connectivity in transition from acute to chronic tinnitus. Human brain mapping, 42(2), 485–494. https://doi.org/10.1002/hbm.25238
- Langguth, B., Kreuzer, P. M., Kleinjung, T., & De Ridder, D. (2013). Tinnitus: Causes and clinical management. The Lancet Neurology, 12(9), 920930. https://doi.org/10.1016/S1474-4422(13)70160-1
- Lee, J. B., & Cherney, L. R. (2018). Tau-U : A Quantitative Approach for Analysis of Single-Case Experimental Data in Aphasia. American Journal of Speech-Language Pathology, 27(1S), 495503. https://doi.org/10.1044/2017_AJSLP-16-0197
- Li, Y. S., Nassar, M. R., Kable, J. W., & Gold, J. I. (2019). Individual Neurons in the Cingulate Cortex Encode Action Monitoring, Not Selection, during Adaptive Decision-Making. *The Journal of neuroscience: the official journal of the Society for Neuroscience*, *39*(34), 6668–6683. https://doi.org/10.1523/JNEUROSCI.0159-19.2019
- Lima, D. O., Araújo, A. M. G. D. D., Branco-Barreiro, F. C. A., Carneiro, C. D. S., Almeida, L. N. A., & Rosa, M. R. D. D. (2020). Auditory attention in individuals with tinnitus. Brazilian Journal of Otorhinolaryngology, 86(4), 461467. https://doi.org/10.1016/j.bjorl.2019.01.011
- Liu, C., Glowatzki, E., & Fuchs, P. A. (2015). Unmyelinated type II afferent neurons report cochlear damage. Proceedings of the National Academy of Sciences of the United States of America, 112(47), 14723–14727. https://doi.org/10.1073/pnas.1515228112
- Llinás, R.R., Ribary, U., Jeanmonod, D., Kronberg, E. and Mitra, P.P., 1999. Thalamocortical dysrhythmia: a neurological and neuropsychiatric syndrome characterized by magnetoencephalography. Proceedings of the National Academy of Sciences, 96(26), pp.15222-15227.
- Londero, A., Bonfils, P., & Lefaucheur, J. P. (2018). Transcranial magnetic stimulation and subjective tinnitus. A review of the literature, 2014-2016. European annals of otorhinolaryngology, head and neck diseases, 135(1), 51–58. https://doi.org/10.1016/j.anorl.2017.12.001
- Lurquin, Ph., et Coulon, E. (2016). Du bon usage de l'acouphénométrie. Cahiers de l'Audition, 3,28-31.
- Manolov, R., Moeyaert, M., & Evans, J. (2016). Single-case data analysis: Software resources for applied researchers. https://doi.org/10.13140/RG.2.1.3981.7766
- Manolov, R., & Solanas, A. (2018). Analytical Options for Single-Case Experimental Designs: Review and Application to Brain Impairment. Brain Impairment, 19(1), 1832. https://doi.org/10.1017/BrImp.2017.17
- Marié-Bailly, I. (2016). L'art de l'accordage relationnel : apport du spectrogramme dans la relation "accompagné-accompagnant", De la voix parlée au chant p 577 (Klein-Dallant).
- Marié-Bailly, I., & Varquet, H. (2020). De la musicothérapie à la musico-phonologie à distance. Revue française de musicothérapie, 39(1). https://hal.science/hal-03432603
- Martins, M. L., Kleinjung, T., Meyer, M., Raveenthiran, V., Wellauer, Z., Peter, N., & Neff, P. (2022). Transcranial electric and acoustic stimulation for tinnitus: study protocol for a randomized double-blind controlled trial assessing the influence of combined transcranial random noise and acoustic stimulation on tinnitus loudness and distress. Trials, 23(1), 418. https://doi.org/10.1186/s13063-022-06253-5
- Mavrogeni, P., Maihoub, S., Tamás, L., & Molnár, A. (2022). Tinnitus characteristics and associated variables on Tinnitus Handicap Inventory among a Hungarian population. Journal of Otology, 17(3), 136139. https://doi.org/10.1016/j.joto.2022.04.003

- Menin-Sicard, A., & Sicard, E. (2003). VOCALAB, logiciel d'aide à l'évaluation et à la rééducation de la voix. Revue de laryngologie - otologie - rhinologie, 124(5), 311–314. https://pubmed.ncbi.nlm.nih.gov/15144027/
- Menin-Sicard, A., Sicard, E., & Bézard, M. (2016). Intérêt de la visualisation de la position et du mouvement des articulateurs pour améliorer l'intelligibilité : Plate-forme Diadolab. XVIe Rencontres Internationales d'Orthophonie - ORTHOPHONIE ET TECHNOLOGIES INNOVANTES, UNADREO. 261-290.
- Menin-Sicard, A. (2021). Fiche Thérapeutique n°16—La Rééducation vocale en téléconsultation. https://sante.gerip.com
- Michelet, T., Bioulac, B., Guehl, D., Escola, L., & Burbaud, P. (2007). Impact of commitment on performance evaluation in the rostral cingulate motor area. The Journal of neuroscience: the official journal of the Society for Neuroscience, 27(28), 7482–7489. https://doi.org/10.1523/JNEUROSCI.4718-06.2007
- Milner, R., Lewandowska, M., Ganc, M., Nikadon, J., Niedziałek, I., Jędrzejczak, W. W., & Skarżyński, H. (2020). Electrophysiological correlates of focused attention on low- and high-distressed tinnitus. PloS one, 15(8), e0236521. https://doi.org/10.1371/journal.pone.0236521
- Nair, G. (1999). Voice Tradition and Technology: A State-of-the-art Studio. Singular Publishing Group.
- Nascimento, I. D. P., Almeida, A. A., Diniz, J., Martins, M. L., Freitas, T. M. M. W. C. D., & Rosa, M. R. D. D. (2019). Tinnitus evaluation : Relationship between pitch matching and loudness, visual analog scale and tinnitus handicap inventory. Brazilian Journal of Otorhinolaryngology, 85(5), 611616. https://doi.org/10.1016/j.bjorl.2018.05.006
- Neff, P., Zielonka, L., Meyer, M., Langguth, B., Schecklmann, M., & Schlee, W. (2019). Comparison of Amplitude Modulated Sounds and Pure Tones at the Tinnitus Frequency: Residual Tinnitus Suppression and Stimulus Evaluation. Trends in Hearing, 23, 233121651983384. https://doi.org/10.1177/2331216519833841
- Newman, C. W., Jacobson, G. P., & Spitzer, J. B. (1996). Development of the Tinnitus Handicap Inventory. Archives of otolaryngology--head & neck surgery, 122(2), 143–148. https://doi.org/10.1001/archotol.1996.01890140029007
- Noreña, A. J., & Farley, B. J. (2013). Tinnitus-related neural activity : Theories of generation, propagation, and centralization. Hearing Research, 295, 161171. https://doi.org/10.1016/j.heares.2012.09.010
- Ni, T., Liu, Y., Xie, H., Yang, S., Wang, Y., Jiang, Y., & Han, Z. (2024). Customized Music Therapy Combined with the Counseling and Follow-Up System: Stratified Results for Tinnitus Efficacy. OTO open, 8(3), e173. https://doi.org/10.1002/oto2.173
- Noreña, A.J., 2011. An integrative model of tinnitus based on a central gain controlling neural sensitivity. Neuroscience & Biobehavioral Reviews, 35(5), pp.1089-1109.
- Noreña, A. J., Lacher-Fougère, S., Fraysse, M.-J., Bizaguet, E., Grevin, P., Thai-Van, H., Moati, L., Le Pajolec, C., Fournier, P., & Ohresser, M. (2021). A contribution to the debate on tinnitus definition. In Progress in Brain Research (Vol. 262, p. 469485). Elsevier. https://doi.org/10.1016/bs.pbr.2021.01.029
- Ohresser, M. (2017). Les acouphènes : Diagnostic, prise en charge et thérapeutique. Elsevier Masson.
- Parker, R. I., Vannest, K. J., Davis, J. L., & Sauber, S. B. (2011). Combining Nonoverlap and Trend for Single-Case Research: Tau-U. Behavior Therapy, 42(2), 284299. https://doi.org/10.1016/j.beth.2010.08.006
- Parmentier, Fl., Marié-Bailly, I., & Pillot-Loiseau, C. (2014). Voir sa voix : comment le spectrogramme facilite la rééducation vocale. Les Entretiens de Bichat. https://hal.science/hal-01099451/file/dfcd0488b2d240f01e6e6f29954e6cce.pdf
- Parmentier, Fl., (2016). Travail vocal et spectrogramme : pourquoi, comment ? De la voix parlée au chant. p p 565 (Klein-Dallant).
- Pedroni A, Bahreini A, Langer N. Automagic: Standardized preprocessing of big EEG data. Neuroimage. 2019 15 ;200 :460–73.

- Perez-Carpena, P., Bibas, A., Lopez-Escamez, J. A., Vardonikolaki, K., & Kikidis, D. (2021). Systematic review of sound stimulation to elicit tinnitus residual inhibition. In Progress in Brain Research (Vol. 262, p. 121). Elsevier. https://doi.org/10.1016/bs.pbr.2021.01.020
- Pienkowski, M. (2021). Loud Music and Leisure Noise Is a Common Cause of Chronic Hearing Loss, Tinnitus and Hyperacusis. International Journal of Environmental Research and Public Health, 18(8), 4236. https://doi.org/10.3390/ijerph18084236
- Prabhu, P., & Nagaraj, M. K. (2020). Development and validation of Hyperacusis Handicap Questionnaire in individuals with tinnitus associated with hyperacusis. Journal of Otology, 15(4), 124128. https://doi.org/10.1016/j.joto.2019.12.004
- Rauschecker, J.P., Leaver, A.M. and Mühlau, M., 2010. Tuning out the noise: limbic-auditory interactions in tinnitus. Neuron, 66(6), pp.819-826.
- Raichle M. E. (2015). The brain's default mode network. *Annual review of neuroscience*, *38*, 433–447. https://doi.org/10.1146/annurev-neuro-071013-014030
- Riha, C., Güntensperger, D., Kleinjung, T., & Meyer, M. (2022). Recovering Hidden Responder Groups in Individuals Receiving Neurofeedback for Tinnitus. Frontiers in neuroscience, 16, 867704. https://doi.org/10.3389/fnins.2022.867704
- Rodríguez-León, I. G., Alonso-Valerdi, L. M., Salido-Ruiz, R. A., Román-Godínez, I., Ibarra-Zarate, D. I., & Torres-Ramos, S. (2022). Effect of Auditory Discrimination Therapy on Attentional Processes of Tinnitus Patients. Sensors, 22(3), 937. https://doi.org/10.3390/s22030937
- Schneider, D. M., & Mooney, R. (2018). How Movement Modulates Hearing. Annual Review of Neuroscience, 41(1), 553572. https://doi.org/10.1146/annurev-neuro-072116-031215
- Searchfield, G. D., Spiegel, D. P., Poppe, T. N. E. R., Durai, M., Jensen, M., Kobayashi, K., Park, J., Russell, B. R., Shekhawat, G. S., Sundram, F., Thompson, B. B., & Wise, K. J. (2021). A proof-of-concept study comparing tinnitus and neural connectivity changes following multisensory perceptual training with and without a low-dose of fluoxetine. International Journal of Neuroscience, 131(5), 433444. https://doi.org/10.1080/00207454.2020.1746310
- Shore, S. E., Roberts, L. E., & Langguth, B. (2016). Maladaptive plasticity in tinnitus--triggers, mechanisms and treatment. Nature reviews. Neurology, 12(3), 150–160. https://doi.org/10.1038/nrneurol.2016.12
- Smeele, S. J., Adhia, D. B., & De Ridder, D. (2023). Feasibility and Safety of High-Definition Infraslow Pink Noise Stimulation for Treating Chronic Tinnitus-A Randomized Placebo-Controlled Trial. Neuromodulation: journal of the International Neuromodulation Society, 26(4), 801–816. https://doi.org/10.1016/j.neurom.2022.10.049
- Straka, G. (1965). Album phonétique. Presses de l'Université Laval.
- Tabbal, J., Kabbara, A., Khalil, M., Benquet, P., & Hassan, M. (2021). Dynamics of task-related electrophysiological networks: a benchmarking study. *NeuroImage*, *231*, 117829.
- Tarlow, K. R. (2016). Baseline corrected Tau calculator. http://www.ktarlow.com/stats/tau
- Tate, R. L., & Perdices, M. (2019). Single-Case Experimental Designs for Clinical Research and Neurorehabilitation Settings: Planning, Conduct, Analysis and Reporting (1re éd.). Routledge. https://doi.org/10.4324/9780429488184
- Tate, R. L., Perdices, M., Rosenkoetter, U., McDonald, S., Togher, L., Shadish, W., Horner, R., Kratochwill, T., Barlow, D. H., Kazdin, A., Sampson, M., Shamseer, L., & Vohra, S. (2016). The Single-Case Reporting Guideline In BEhavioural Interventions (SCRIBE) 2016 : Explanation and elaboration. Archives of Scientific Psychology, 4(1), 1031. https://doi.org/10.1037/arc0000027
- Tonndorf J. (1987). The analogy between tinnitus and pain: a suggestion for a physiological basis of chronic tinnitus. Hearing research, 28(2-3), 271–275. https://doi.org/10.1016/0378-5955(87)90054-2

- Tsai, M.-H. (2020). Noise Induced Hearing Loss and Tinnitus—New Research Developments and Remaining Gaps in Disease Assessment, Treatment, and Prevention. Brain Sciences, 10(10), 732. https://doi.org/10.3390/brainsci10100732
- Vanneste, S., Song, J.J. and De Ridder, D., (2018a). Thalamocortical dysrhythmia detected by machine learning. Nature communications, 9(1), pp.1-13.
- Vanneste, S., Joos, K., Ost, J., De Ridder, D., (2018b). Influencing connectivity and cross-frequency coupling by real-time source localized neurofeedback of the posterior cingulate cortex reduces tinnitus related distress. Neurobiology of stress, 8, pp.211-224.
- Vanneste, S., Byczynski G., Verplancke T., Ost J., Song J.J., De Ridder D., Switching tinnitus on or off: An initial investigation into the role of the pregenual and rostral to dorsal anterior cingulate cortices., NeuroImage (2024), https://doi.org/10.1016/j.neuroimage.2024.120713
- Wallace, M. N., & Palmer, A. R. (2023). Neural Plasticity in Tinnitus Mechanisms. Brain sciences, 13(12), 1615. https://doi.org/10.3390/brainsci13121615
- Wang, T.-C., Chang, T.-Y., Tyler, R., Lin, Y.-J., Liang, W.-M., Shau, Y.-W., Lin, W.-Y., Chen, Y.-W., Lin, C.-D., & Tsai, M.-H. (2020). Noise Induced Hearing Loss and Tinnitus—New Research Developments and Remaining Gaps in Disease Assessment, Treatment, and Prevention. Brain Sciences, 10(10), 732. https://doi.org/10.3390/brainsci10100732
- Wang, C. D., Zhu, X. R., Zhou, X., Li, J., Lan, L., Huang, D., Zheng, Y., & Cai, Y. (2023). Cross-Subject Tinnitus Diagnosis Based on Multi-Band EEG Contrastive Representation Learning. IEEE journal of biomedical and health informatics, 27(7), 3187–3197. https://doi.org/10.1109/JBHI.2023.3264521
- Won, J. Y., Yoo, S., Lee, S. K., Choi, H. K., Yakunina, N., Le, Q., et Nam, E.-C. (2013). Prevalence and factors associated with tinnitus neck and jaw muscle modulation. Audiology and Neurotology, 18(4), 261273. https://doi.org/10.1159/000351685
- Wu, C., Stefanescu, R. A., Martel, D. T., & Shore, S. E. (2016). Tinnitus : Maladaptive auditory–somatosensory plasticity. Hearing Research, 334, 2029. https://doi.org/10.1016/j.heares.2015.06.005
- Yadollahpour, A., Rashidi, S., Saki, N., Kunwar, P. S., & Mayo-Yáñez, M. (2024). Repeated Bilateral Transcranial Direct Current Stimulation over Auditory Cortex for Tinnitus Treatment: A Double-Blinded Randomized Controlled Clinical Trial. Brain sciences, 14(4), 373. https://doi.org/10.3390/brainsci14040373
- Yasoda-Mohan, A., Faubert, J., Ost, J., Kropotov, J. D., & Vanneste, S. (2024). Investigating sensitivity to multi-domain prediction errors in chronic auditory phantom perception. Scientific reports, 14(1), 11036. https://doi.org/10.1038/s41598-024-61045-y
- Yasoda-Mohan, A., Chen, F., Ó Sé, C., Allard, R., Ost, J., & Vanneste, S. (2024). Phantom perception as a Bayesian inference problem: a pilot study. Journal of neurophysiology, 131(6), 1311–1327. https://doi.org/10.1152/jn.00349.2023
- Yasoda-Mohan, A., & Vanneste, S. (2024). Development, Insults and Predisposing Factors of the Brain's Predictive Coding System to Chronic Perceptual Disorders-A Life-Course Examination. Brain sciences, 14(1), 86. https://doi.org/10.3390/brainsci14010086
- Yuan, Y., Cai, Y., Wu, J., Li, J., Huang, X., Chen, G., Lan, L., Huang, H., Dong, H., Zheng, Y., & Yue, Z. (2022). Tinnitus Affects Endogenous But Not Exogenous Auditory Attention Orienting. American journal of audiology, 31(3), 737–745. https://doi.org/10.1044/2022_AJA-21-00128
- Zhang, J., Huang, S., Nan, W., Zhou, H., Wang, J., Wang, H., Salvi, R., & Yin, S. (2021). Switching Tinnitus-On: Maps and source localization of spontaneous EEG. Clinical neurophysiology: official journal of the International Federation of Clinical Neurophysiology, 132(2), 345–357. https://doi.org/10.1016/j.clinph.2020.10.023

- Zeman, F.; Koller, M.; Figueiredo, R.; Aazevedo, A.; Rates, M.; Coelho, C.; Kleinjung, T.; de Ridder, D.; Langguth, B.; Landgrebe, M. Tinnitus Handicap Inventory for Evaluating Treatment Effects. Otolaryngol. Head. Neck Surg. **2011**, 145, 282–287. [Google Scholar] [CrossRef] [PubMed]
- Zhu, X., Wang, F., Hu, H., Sun, X., Kilgard, M. P., Merzenich, M. M., & Zhou, X. (2014). Environmental Acoustic Enrichment Promotes Recovery from Developmentally Degraded Auditory Cortical Processing. The Journal of Neuroscience, 34(16), 54065415. https://doi.org/10.1523/JNEUROSCI.5310-13.2014

15. List of Appendices

APPENDIX 1: Recruitment poster, social media announcement

APPENDIX 2: Siopi application to download to fill in the questionnaire

ANNEX 3: Tinnitus Handicap Index, Paper Version

TINNITUS HANDICAP INVENTORY (THI)
Version française validée par Ghulyan-Bédikian et coll.(2010)*

	I T E M	REPONSE		
		4	2	0
1F	A cause de votre acouphène, vous est-il difficile de vous concentrer ?	OUI	PARFOIS	NON
2F.	A cause de l'intensité de votre acouphène, vous est-il difficile d'entendre les personnes qui vous entourent ?	OUI	PARFOIS	NON
3E.	Votre acouphène vous rend-il coléreux ?	OUI	PARFOIS	NON
4F.	Vous sentez-vous l'esprit confus(e) à cause de votre acouphène ?	OUI	PARFOIS	NON
5C.	A cause de votre acouphène vous sentez-vous désespéré(e) ?	OUI	PARFOIS	NON
6E.	Vous plaignez-vous beaucoup de votre acouphène ?	OUI	PARFOIS	NON
7F.	A cause de votre acouphène, avez-vous du mal à trouver le sommeil la nuit ?	OUI	PARFOIS	NON
8C.	Avez-vous le sentiment de ne pas pouvoir vous libérer de votre acouphène ?	OUI	PARFOIS	NON
9F.	Votre acouphène interfère-t-il dans votre plaisir à pratiquer des activités sociales (aller au restaurant, au cinéma…) ?	OUI	PARFOIS	NON
10E.	A cause de votre acouphène, vous sentez-vous frustré(e) ?	OUI	PARFOIS	NON
11C.	A cause de votre acouphène, avez-vous le sentiment d'être atteint(e) d'une maladie grave ?	OUI	PARFOIS	NON
12F.	A cause de votre acouphène vous est-il difficile de profiter pleinement de la vie ?	OUI	PARFOIS	NON
13F.	Votre acouphène, interfère-t-il dans vos responsabilités au travail ou à la maison ?	OUI	PARFOIS	NON
14E.	Trouvez-vous que vous êtes souvent irritable à cause de votre acouphène ?	OUI	PARFOIS	NON
15F	A cause de votre acouphène, vous-est-il difficile de lire ?	OUI	PARFOIS	NON
16E	Etes-vous contrarié(e) ou bouleversé(e) par votre acouphène ?	OUI	PARFOIS	NON
17E.	Pensez-vous que votre problème d'acouphène a installé un stress dans vos relations avec les membres de votre famille et vos amis ?	OUI	PARFOIS	NON
18F.	Vous-est-il difficile de vous concentrer sur autre chose que votre acouphène ?	OUI	PARFOIS	NON
19C.	Pensez-vous ne pas avoir de contrôle sur votre acouphène ?	OUI	PARFOIS	NON
20F.	A cause de votre acouphène, vous sentez-vous souvent fatigué(e) ?	OUI	PARFOIS	NON
21E.	A cause de votre acouphène, vous sentez-vous déprimé(e) ?	OUI	PARFOIS	NON
22E.	Votre acouphène vous rend-il anxieux(se) ?	OUI	PARFOIS	NON
23C.	Pensez-vous ne plus pouvoir faire face à votre acouphène ?	OUI	PARFOIS	NON
24F.	Votre acouphène s'aggrave-t-il quand vous êtes stressé(e) ?	OUI	PARFOIS	NON
25E.	Votre acouphène vous donne-t-il un sentiment d'incertitude ?	OUI	PARFOIS	NON
	**TOTAL**			

SCORE TOTAL (faites le total du nombre de points et comparez le résultat par rapport aux niveaux ci-dessous)

**De 0 à 16**	Handicap léger (uniquement perceptible dans un environnement calme	**NIVEAU 1**
**De 18 à 36**	Handicap modéré (facilement couvert par des sons provenant de l'environnement et facilement oublié lors d'activités)	**NIVEAU 2**
**De 38 à 56**	Handicap moyen (perçu en présence d'un bruit de fond : les activités quotidiennes peuvent malgré tout être effectuées)	**NIVEAU 3**
**De 58 À 76**	Handicap lourd (presque toujours perceptible, il conduit au dérèglement des structures du sommeil et est susceptible de porter atteinte aux activités quotidiennes)	**NIVEAU 4**
**De 76 à 100**	Handicap invalidant (constamment perceptible, structures du sommeil déréglées, difficultés rencontrées pour toute activité)	**NIVEAU 5**

F = sous-échelle fonctionnelle – E = sous-échelle émotionnelle – C = sous-échelle catastrophique :
score total : .... F .... E C

* Propriétés psychométriques d'une version française du Tinnitus Handicap Inventory. Vghulyan-Bedikian, M. Paolino, F. Giorgetti-D'Escleres, F.Paolino. L'Encéphale (2010 36,390-396)

Newman CW, Jacobson GP, Spitzer JB. Development of the Tinnitus Handicap Inventory. Arch Otolaryngol Head Neck Surg. 1996 Feb ;122(2):143–8.
Ghulyan-Bédikian V, Paolino M, Giorgetti-D'Esclercs F, Paolino F. [Psychometric properties of a French adaptation of the Tinnitus Handicap Inventory]. Encephale. 2010 Oct ;36(5):390–6

APPENDIX 4: Analog Visual Embarrassment Scales (EVA-G) on the Siopi application

(n°1-tinnitus; n°2-noises; n°3-cervical; n°5-jaw; n°7 sleep; n°11-stress anxiety)

1. On a scale of 0 to 10, is your tinnitus bothering you ?

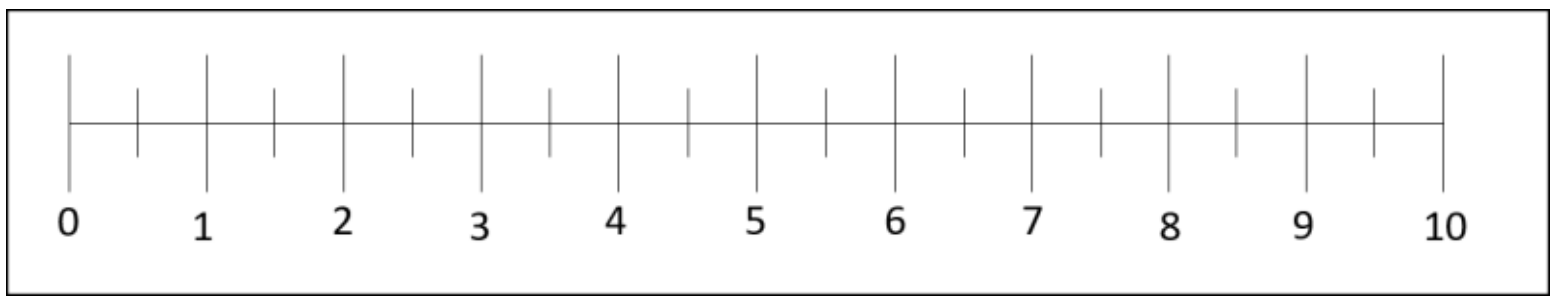

2. On a scale of 0 to 10, do outside noises bother you? ?

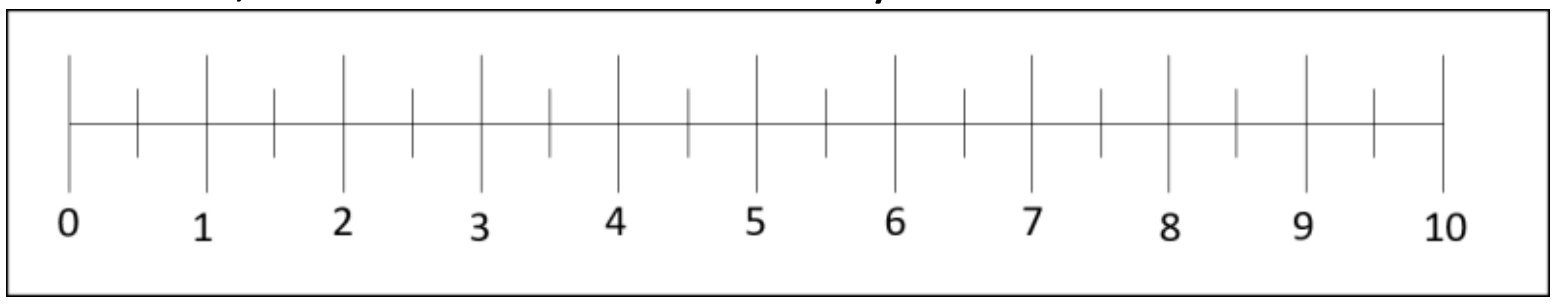

3. On a scale of 0 to 10, do you have neck and/or neck problems ?

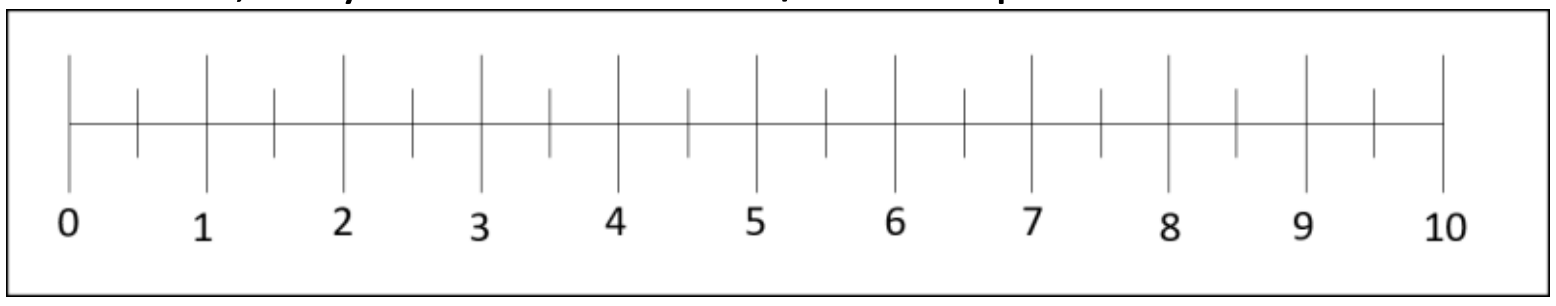

5. On a scale of 0 to 10, do you have jaw problems ?

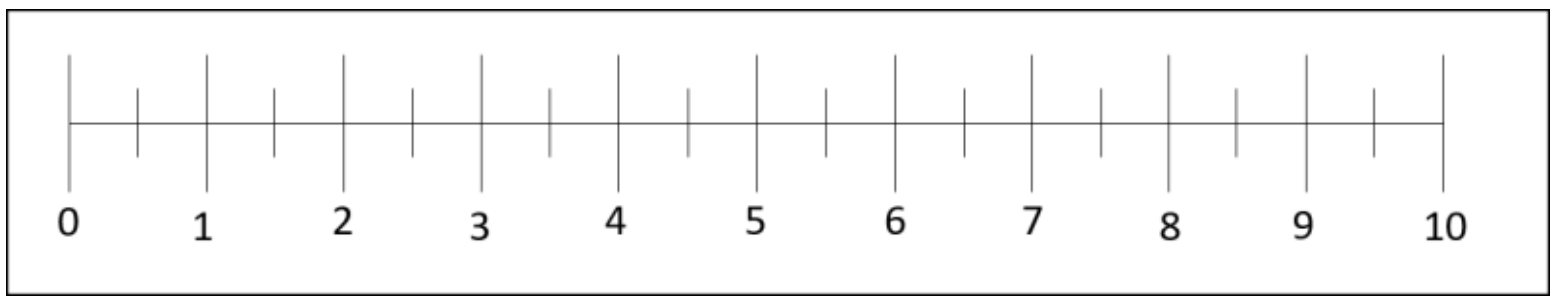

7. On a scale of 0 to 10, do you have sleep problems ?

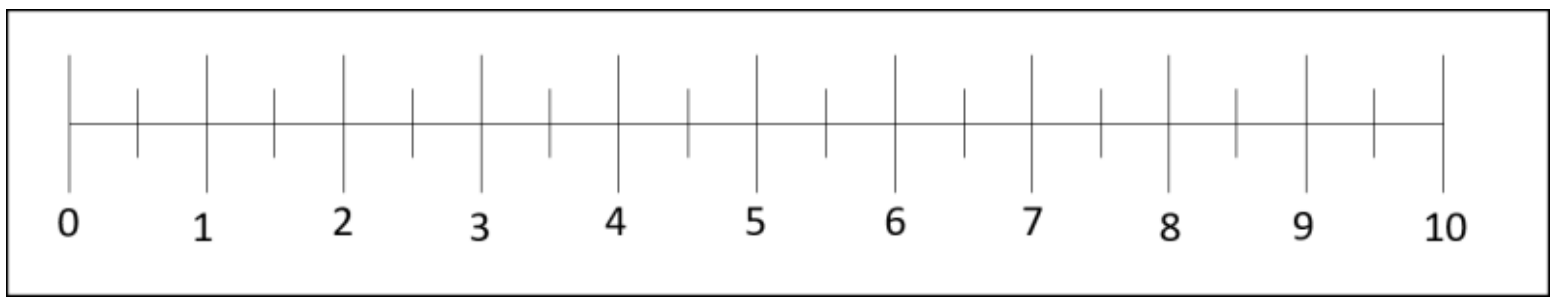

11. On a scale of 0 to 10, are you stressed or anxious ?

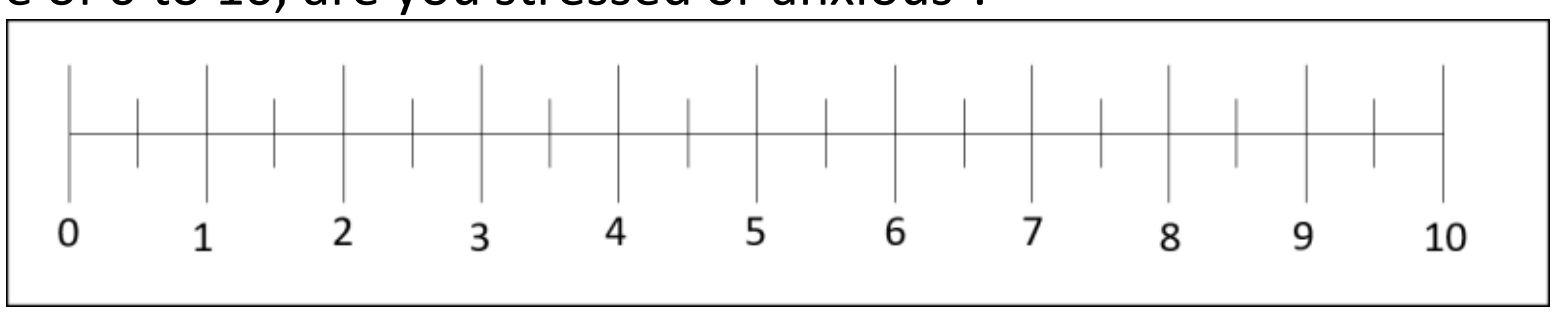

Guillard R.,Hessas A.,Korczowski L., Londero A., Congedo M., Vincent Loche V. (2023). Comparing Clustering Methods Applied to Tinnitus within a Bootstrapped and Diagnostic-Driven Semi-Supervised Framework. Brain Sci. 2023, 13(4), 572; https://doi.org/10.3390/brainsci13040572

APPENDIX 5: Insomnia Severity Index

A = None; L = Light; M = Average; T = Very; E = Extremely; TS = Very satisfied; S = Satisfied; PN = rather neutral; I = Dissatisfied; IT = Very Dissatisfied

	A	L	M	T	E
Please estimate your difficulty falling asleep					
Please estimate your difficulty staying asleep					
Please estimate the severity of your problem of waking up too early in the morning					
To what extent do you consider your sleep difficulties to DISRUPT your daily functioning (e.g., fatigue, concentration, memory, mood)?					
How APPARENT do you consider your sleep difficulties to be to others in terms of deteriorating quality of life?					
How WORRIED are you about your sleep difficulties?					
	**TS**	**S**	**PN**	**I**	**TI**
How SATISFIED/DISSATISFIED ARE YOU with your current sleep?					

APPENDIX 6: GRABS-I scale evaluating the timbre of the voice

	Echelle	Description
**G**	GRADE	The overall level of the voice
**R**	ROUGH	The hoarseness of the voice
**B**	BREATHY	If the voice is blown
**A**	ASTHENIC	Fatigue, low intensity, lack of harmonics
**S**	STRAINED	Tightening and laryngeal forcing
**I**	INSTABILITY	Voice quality variation

Hirano, M. (1981). Psycho-acoustic evaluation of voice: GRABS scale for evaluating the hoarse voice. Clinical Evaluation of Voice, Springer Verlag, Wien.

Dejonckere, P-H., Remacle M., Fresnel-Elbaz E., Woisard V., Crevier-Buchman L. & Millet B. (1996). Differentiated perceptual evaluation of pathological voice quality: reliability and correlations with acoustic measurements. Rev Laryngol Otol Rhinol, 117, 219-24.

APPENDIX 7: VHI Vocal Questionnaire Assessing Vocal Disability

Questionnaire Voice Handicap Index 10 (VHI-10)

Vous devez répondre à chacune des propositions en ne cochant qu'une case pour chaque question.

J = Jamais ; PJ = Presque jamais ; P = Parfois ; PT = presque toujours ; T = Toujours

		0<br>**J**	1<br>**PJ**	2<br>**P**	3<br>**PT**	4<br>**T**
F1	On m'entend difficilement à cause de ma voix					
F3	On me comprend difficilement dans un milieu bruyant					
P10	On me demande : « Qu'est-ce qui ne va pas avec ta voix ? »					
P14	J'ai l'impression que je dois forcer pour produire la voix					
F16	Mes difficultés de voix limitent ma vie personnelle et sociale					
P17	La clarté est imprévisible					
F19	Je me sens écarté(e) des conversations à cause de ma voix					
F22	Mes problèmes de voix entraînent des pertes de revenus					
E23	Mes problèmes de voix me contrarient					
E25	Je me sens handicapé(e) à cause de ma voix					
**SCORE TOTAL** |___|___|						

I M Verdonck-de Leeuw 1, D J Kuik, M De Bodt, I Guimaraes, E B Holmberg, T Nawka, C A Rosen, A Schindler, R Whurr, V Woisard (2008). Validation of the voice handicap index by assessing equivalence of European translations. Folia Phoniatr Logop. 2008;60(4):173-8. doi: 10.1159/000127836.

V Woisard 1, S Bodin, M Puech (2004). [The Voice Handicap Index: impact of the translation in French on the validation]. Rev Laryngol Otol Rhinol (Bord). 2004 ;125(5) :307-12.

APPENDIX 8: AC40 Audiometer – Tone and Speech Audiometry

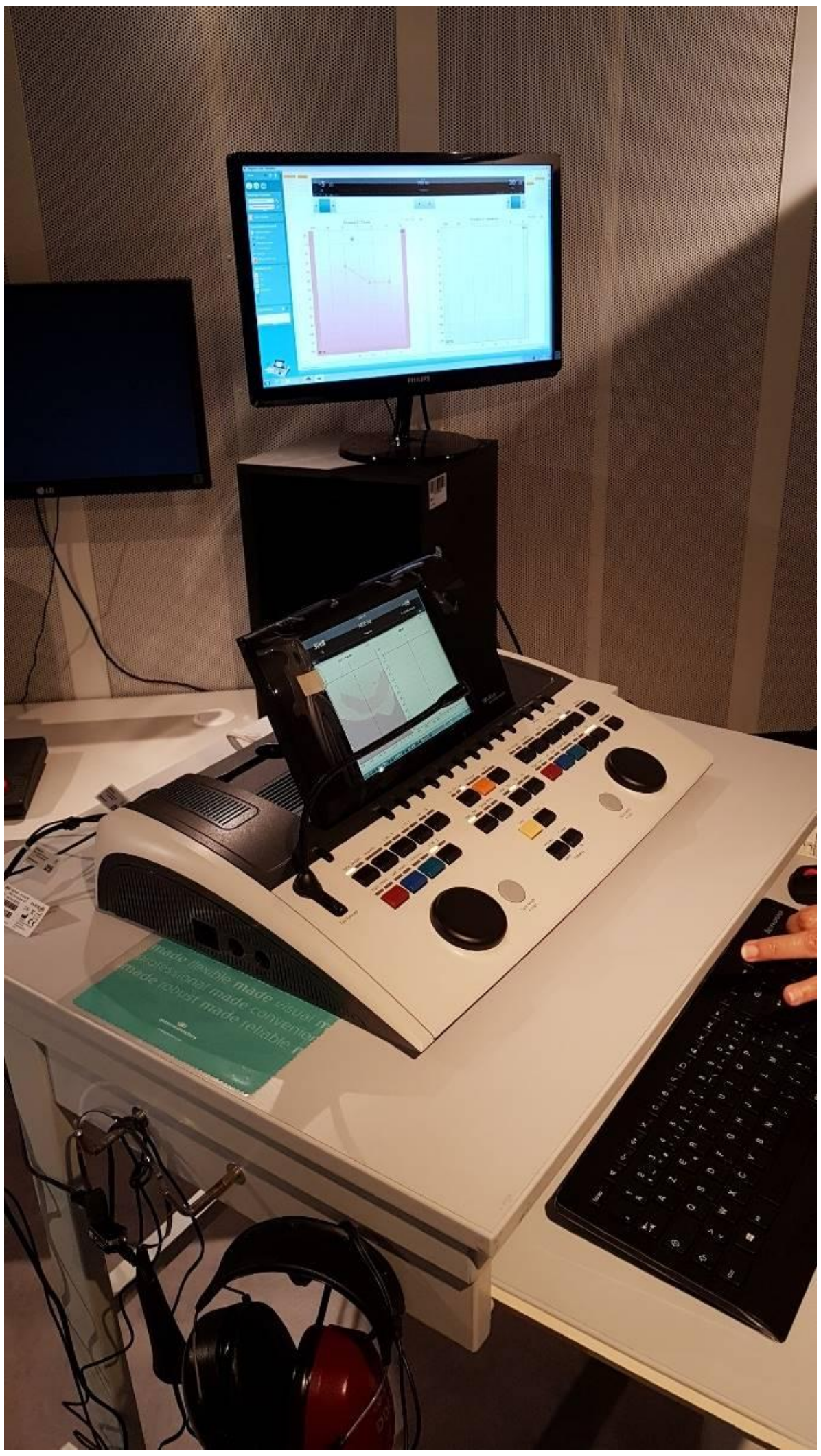

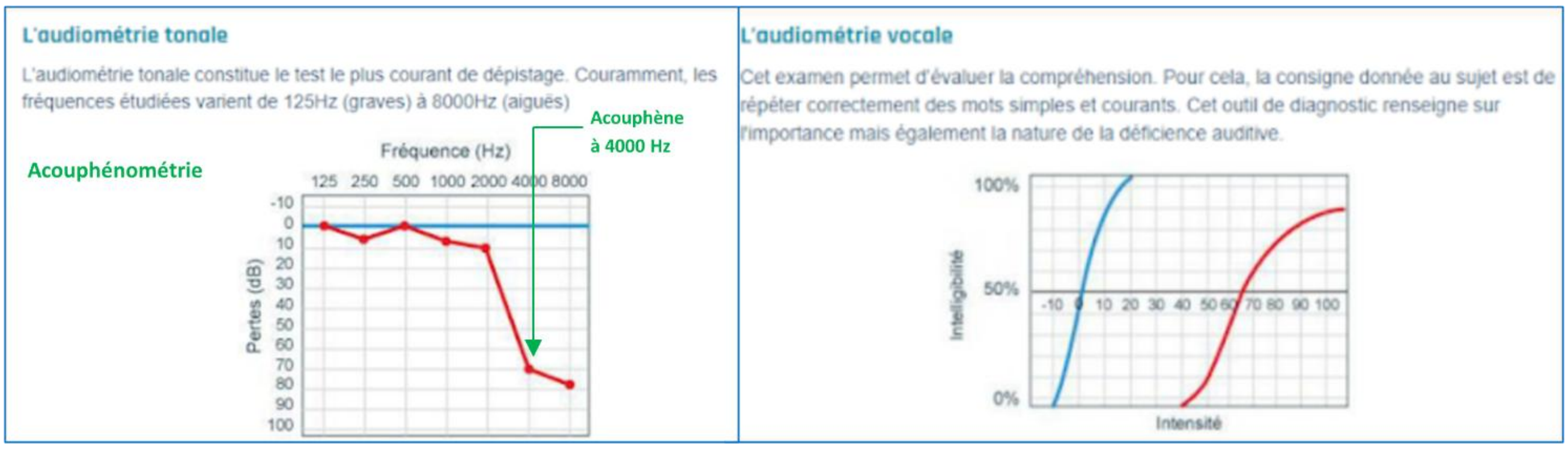

APPENDIX 9 : RAPP TRAINING - VOWEL TRIANGLE - SPECTROGRAM

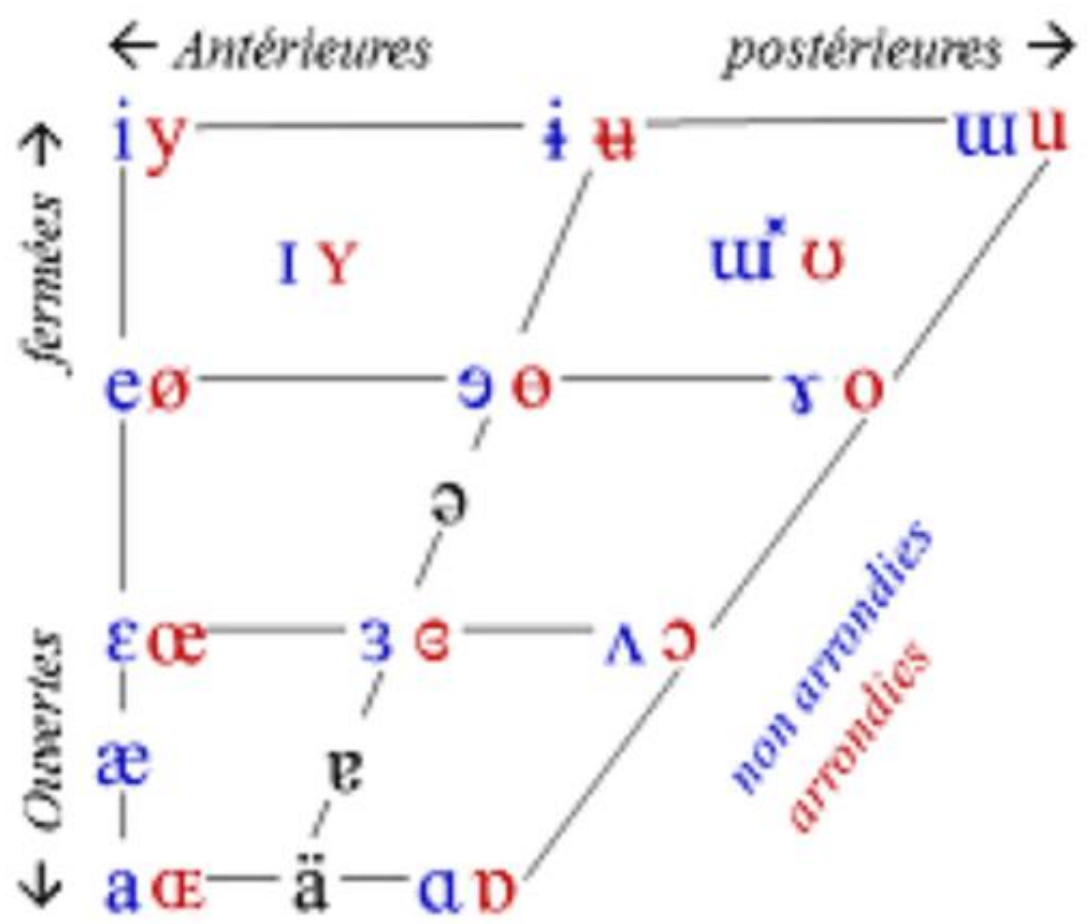

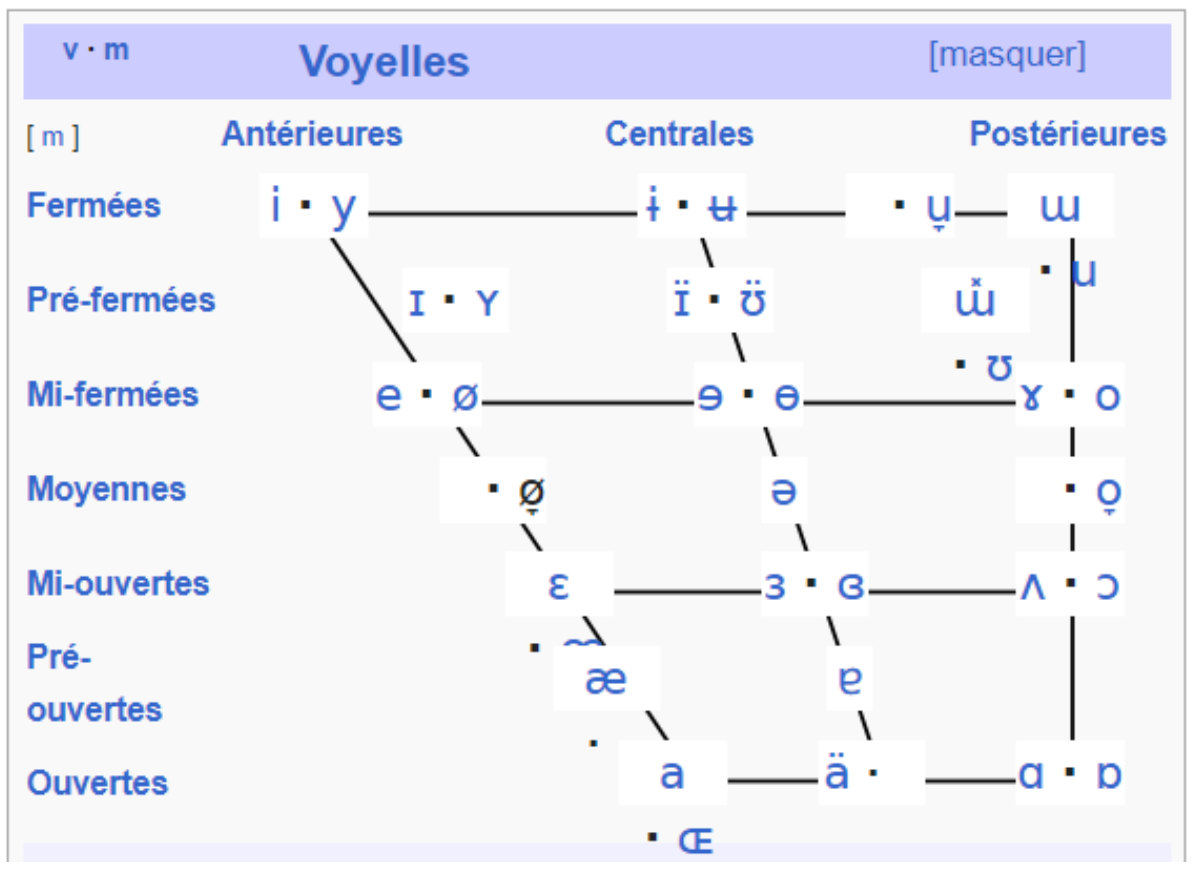

Vowel spectogram image

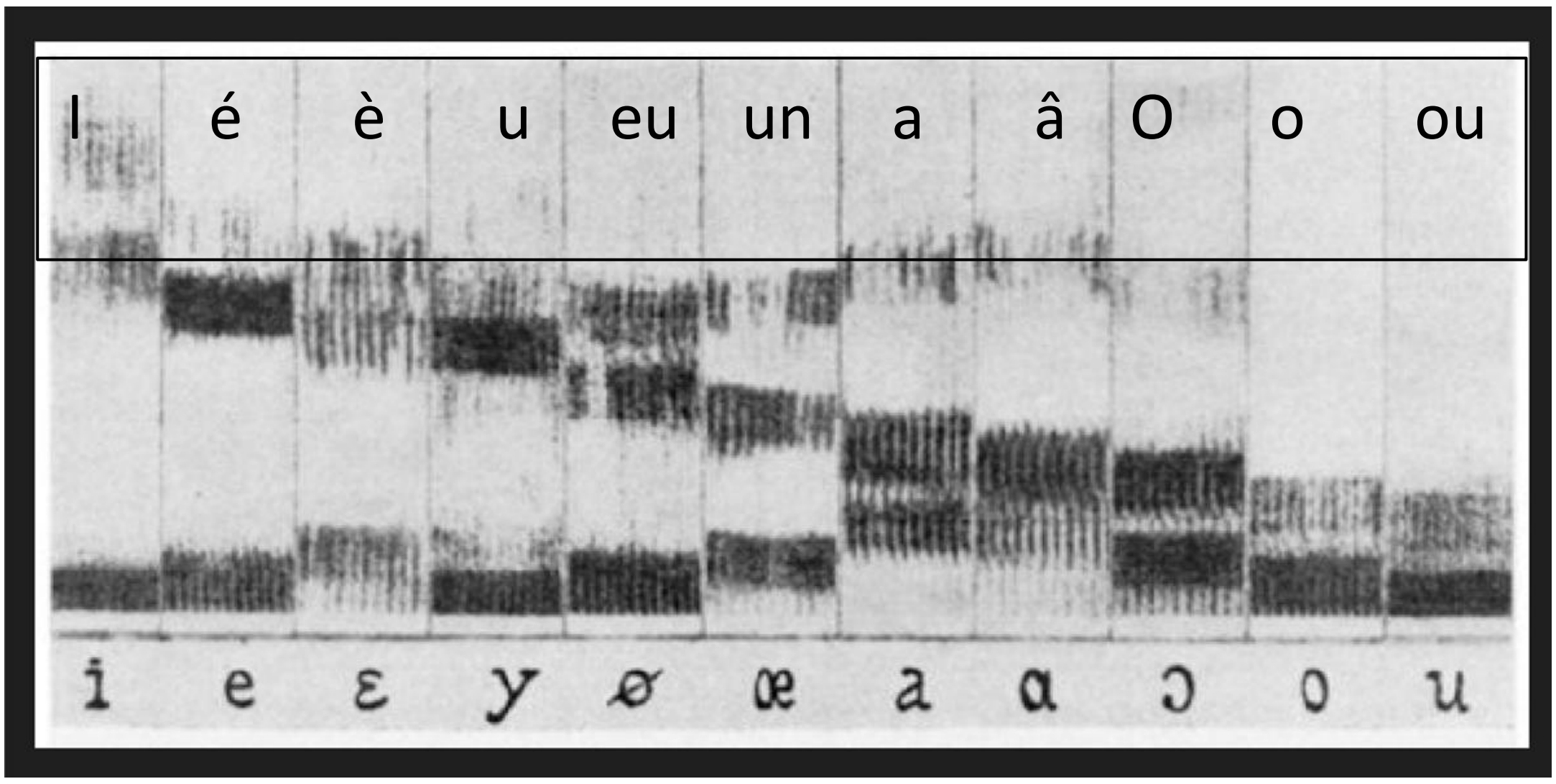

Voce Vista spectrogram used in RAPP training

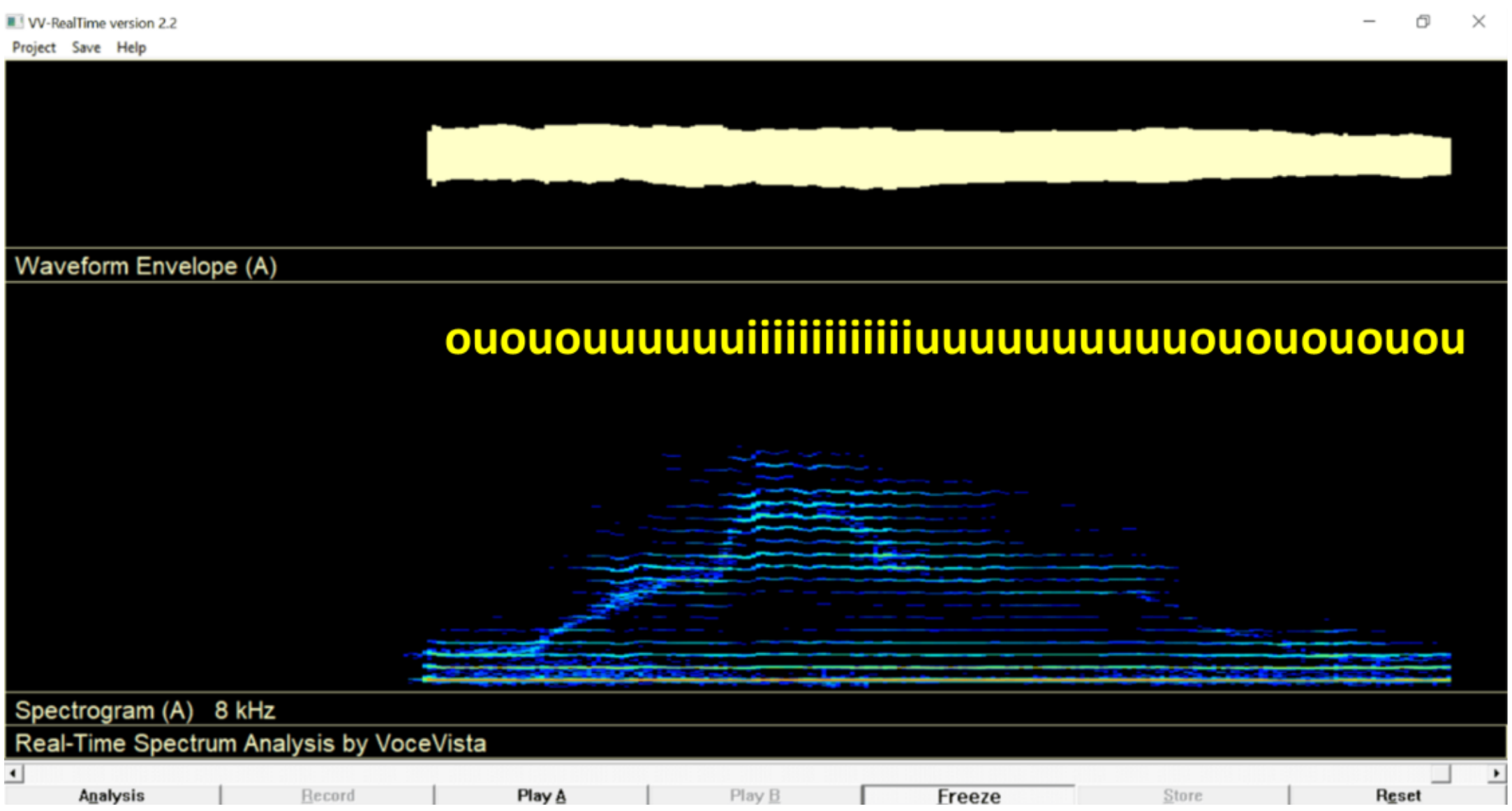

APPENDIX 10: Whistling and Whistling Spectrogram - Residual Inhibition (IR) Testing

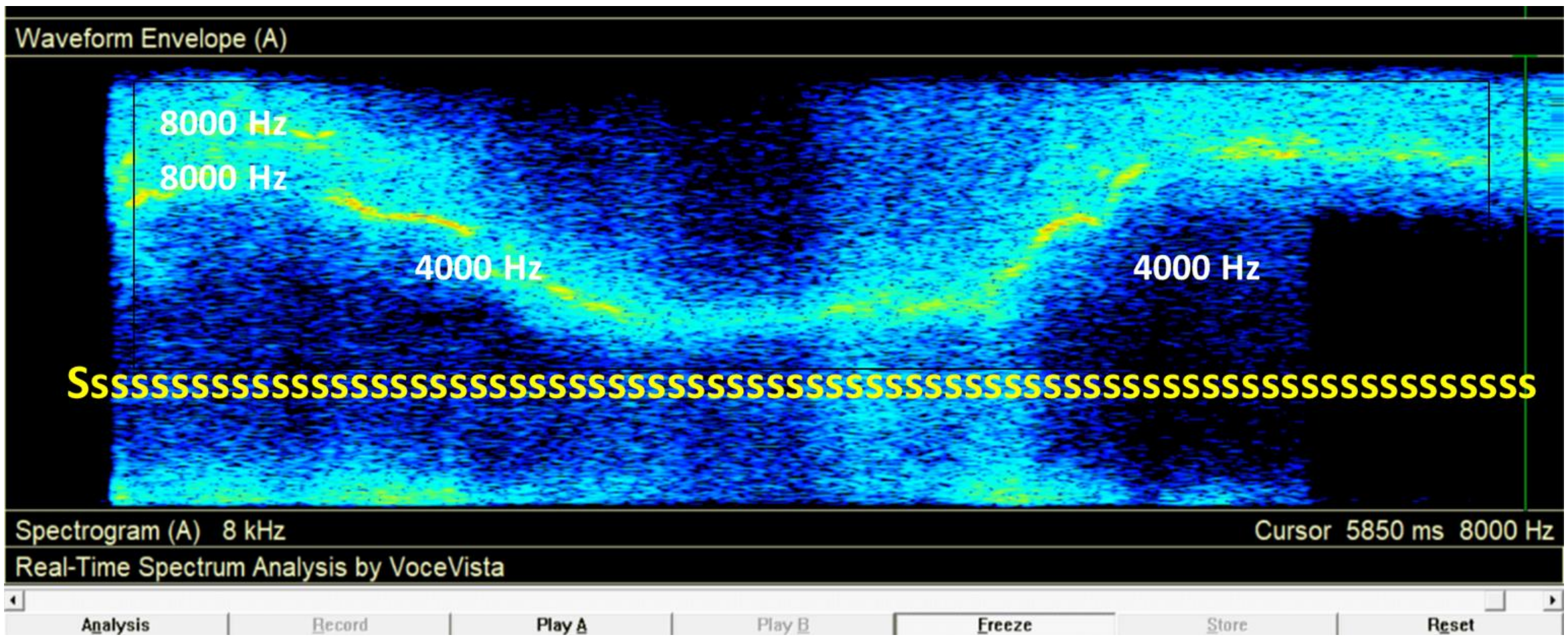

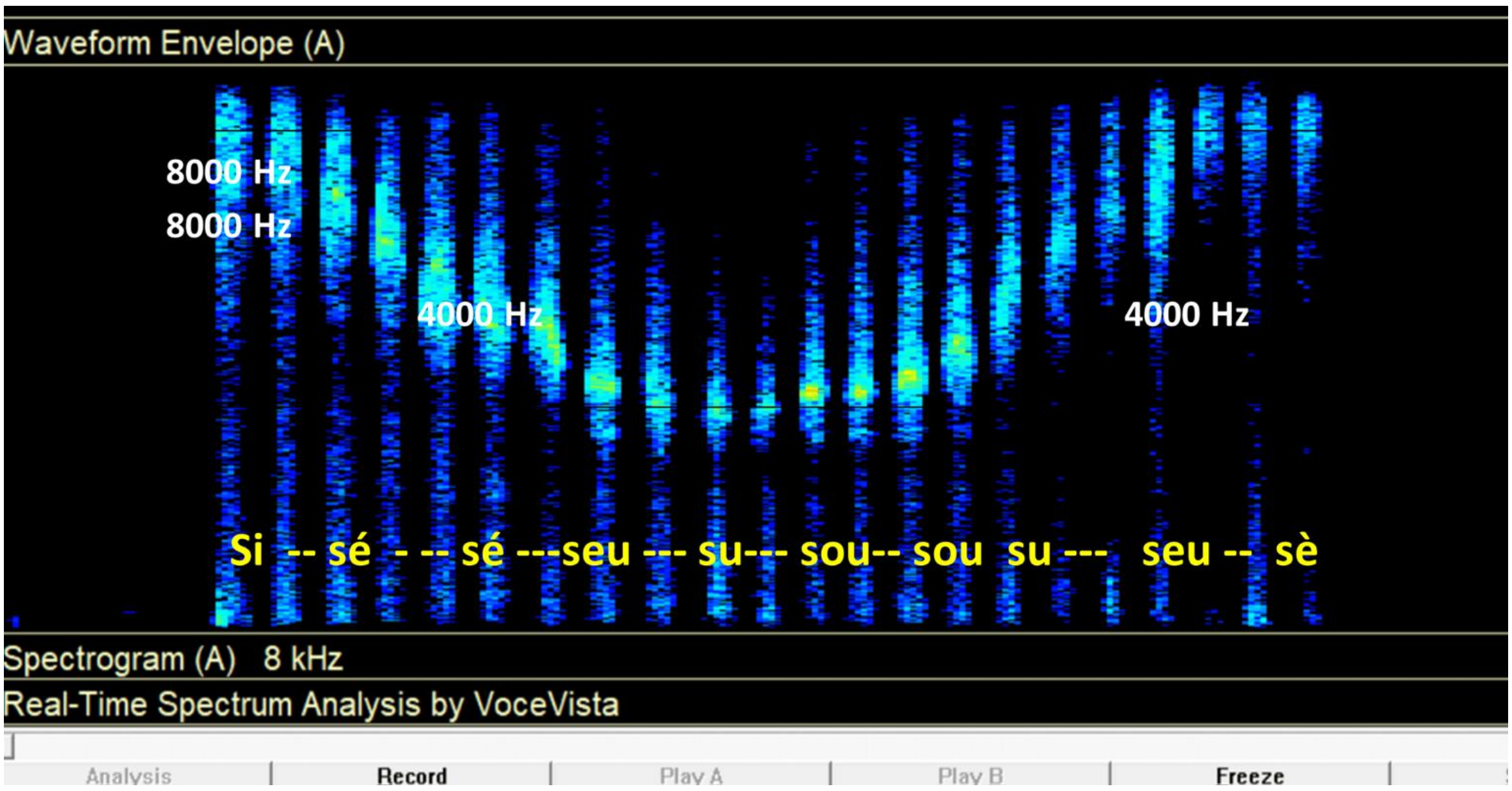

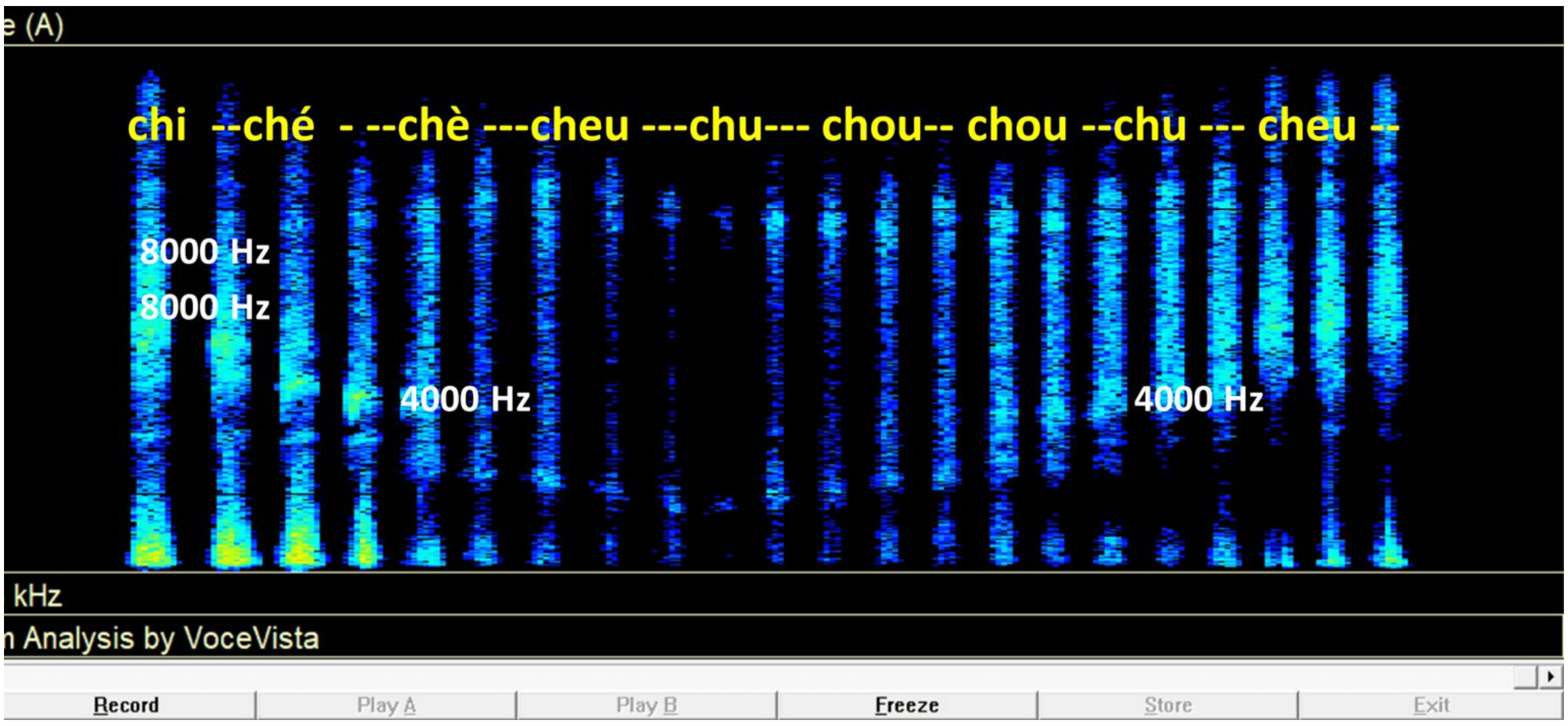

APPENDIX 11: SECURE AND PRIVATE DOCORGA JITSI SOFTWARE, used for videoconferencing

The link: https://meet.jit.si/docorga_64a7f4648f35dcccce516a82_65c5344e43b63cd1cf06cd3a?lang=fr is sent on the first day of training; it is permanent and valid for the 5 videoconferences.

5 minutes avant la séance, à 19h55, les participants (patients, professionnels ou personnes ressources) devront se connecter et attendront d'être invités à rejoindre la réunion en visioconférence qui débutera à 20h.
L'enregistrement de la séance sera réalisé avec l'accord de tous les participants qui peuvent choisir de ne pas ouvrir leur caméra et leur micro, pour garder l'anonymat. Les vidéos des séances seront adressées aux participants présents, de manière à pouvoir les visionner pour s'exercer en autonomie.

Five minutes before the session, at 7:55 p.m., participants (patients, professionals or resource persons) will have to connect and wait to be invited to join the videoconference meeting which will start at 8 p.m.
The recording of the session will be made with the agreement of all participants who may choose not to open their camera and microphone, to remain anonymous. The videos of the sessions will be sent to the participants present, so that they can be viewed to practice independently.
