# Supplementary material for: Neuromodulatory effects of audio-proprio-phonatory reinforcement training on subjective tinnitus, a HD-EEG study protocol
Source: PLoS One. 2026 Nov 6;21(11):e0357455. doi: 10.1371/journal.pone.0357455 (PMC13742906; doi:10.1371/journal.pone.0357455)
Supplement: Supplementary file 3 [file pone.0357455.s004.pdf]

Reviewer's Responses to Questions

Comments to the Author

1. Does the manuscript provide a valid rationale for the proposed study, with clearly identified and justified research questions?

The research question outlined is expected to address a valid academic problem or topic and contribute to the base of knowledge in the field.
Reviewer #1: Yes
Reviewer #2: Yes
Reviewer #3: Yes

---

2. Is the protocol technically sound and planned in a manner that will lead to a meaningful outcome and allow testing the stated hypotheses?

The manuscript should describe the methods in sufficient detail to prevent undisclosed flexibility in the experimental procedure or analysis pipeline, including sufficient outcome-neutral conditions (e.g. necessary controls, absence of floor or ceiling effects) to test the proposed hypotheses and a statistical power analysis where applicable. As there may be aspects of the methodology and analysis which can only be refined once the work is undertaken, authors should outline potential assumptions and explicitly describe what aspects of the proposed analyses, if any, are exploratory.
Reviewer #1: Partly
Reviewer #2: Partly
Reviewer #3: Yes

---

3. Is the methodology feasible and described in sufficient detail to allow the work to be replicable?

Descriptions of methods and materials in the protocol should be reported in sufficient detail for another researcher to reproduce all experiments and analyses. The protocol should describe the appropriate controls, sample size calculations, and replication needed to ensure that the data are robust and reproducible.
Reviewer #1: No
Reviewer #2: Yes
Reviewer #3: Yes

---

4. Have the authors described where all data underlying the findings will be made available when the study is complete?

The PLOS Data policy requires authors to make all data underlying the findings described in their manuscript fully available without restriction, with rare exception, at the time of publication. The data should be provided as part of the manuscript or its supporting information, or deposited to a public repository. For example, in addition to summary statistics, the data points behind means, medians and variance measures should be available. If there are restrictions on publicly sharing data—e.g. participant privacy or use of data from a third party—those must be specified.
Reviewer #1: Yes
Reviewer #2: Yes
Reviewer #3: Yes

---

5. Is the manuscript presented in an intelligible fashion and written in standard English?

PLOS ONE does not copyedit accepted manuscripts, so the language in submitted articles must be clear, correct, and unambiguous. Any typographical or grammatical errors should be corrected at revision, so please note any specific errors here.
Reviewer #1: Yes
Reviewer #2: Yes
Reviewer #3: Yes

---

6. Review Comments to the Author

Reviewer #1: The protocol elements have been thought out rather well. However, from the statistical perspective, there are clarifications and additions needed. Specifically:

1. The authors state that this is a prospective, controlled, block-randomized trial involving two parallel groups (1:1 ratio) of patients. According to Figure 1 it looks like a crossover design. Presumably the intent is the same. Please clarify.

Thank you for this comment. The study is indeed a randomized two-sequence crossover trial. The Figure 1 is correct. The method section (5.1) has been revised accordingly to specify that participants are randomised (1:1) to one of two treatment sequences.

Using a parallel-group design would have meant that one group was excluded from receiving the experimental intervention, which raised ethical concerns. By implementing a crossover design, every participant was given the opportunity to access the intervention, and researchers could still compare outcomes between the RAPP training period and the period without training.

This has been clarified in the Methods section (5.1) of the revised protocol.

2. The recruitment procedure looks fine. What about a retention strategy to minimize missing data.

Thank you for this comment. Regarding the strategy for handling missing data, participants will receive verbal reminders during study visits and interactions only. More intensive reminders were deliberately avoided, as repeatedly prompting participants to assess their tinnitus may increase their focus on it and interfere with the habituation process (which is a key objective of the intervention).

3. The sample size calculation looks reasonable. However, the investigators note that they will compare the change in THI score from T0 to T1 in group A, which receives APPR training, with the change in THI score from T0 to T1 in group B, which does not receive training. According to Figure 1 and Table 1 Group B receives training later. Again, is this merely a crossover design?

Thank you for your comment. You are right; this study uses a two-sequence crossover design. We selected the crossover approach mainly for ethical reasons, so that every participant would have the opportunity to receive the experimental intervention.

We do not currently have a clear hypothesis regarding the duration or persistence of the intervention effects over time, and this aspect is itself one of the exploratory objectives of the study. Therefore, a residual or carry-over effect between periods cannot be excluded.

For this reason, the comparison of THI score after APPR training versus no training is intentionally restricted to the first study period only (T0 to T1).

This has been clarified in the Methods section (5.1) of the revised protocol.

4. The Data management section needs a quality control component to assure consistency of data collection and recording.

We thank the reviewer for this pertinent observation. We have completed the "Data Management" section by including a section dedicated to quality control. This specifies the procedures put in place to ensure consistency in data collection and recording, including systematic verification of data at input, the use of standardized formats and predefined value ranges, as well as regular audits of the data collected to detect any anomalies or inconsistencies. These measures are in line with the best practices in force in the field and aim to guarantee the reproducibility and reliability of the analyses.

We hope that these clarifications meet the reviewer's request.

5. The Data analysis section looks reasonable with the univariate comparisons for change at T1. However, there are multiple tests being done. They are:
A. VAS-A tinnitus scales will be compared between the two groups (from T0 to T1) using the same statistical tests (Student's t-test, or Student's test, or Wilcoxon-Mann-Whitney test, as needed.
B. The VAS scales related to the five associated questions (noise, neck, jaw, sleep, stress-anxiety) will be compared between the two groups (from T0 to T1)
C. The stability over time of the VAS-A tinnitus scales and the THI score will be examined in group A, between T1 and T2 (post-training period).

One has to assure a proper post-hoc test for multiple comparisons adjustment for each A, B, C section testing. The mixed model procedure should accommodate the post hoc adjustment for part C. A Statistician can help with this. For parts A and B, a multiple testing caution should be noted since analysis within each of the multiple scales represents a need for p-value adjustment.

We thank the reviewer for this helpful comment.

We acknowledge the need to clarify our approach to multiple comparisons. The Statistical Analysis section now specifies that the primary THI analysis is designated as the sole confirmatory hypothesis test, while secondary outcome analyses are categorized as exploratory. Furthermore, we have indicated that appropriate adjustments for multiple testing, such as the Holm–Bonferroni correction, will be applied whenever multiple pairwise comparisons occur within the same family of secondary outcomes (e.g., VAS scales). Similarly, post hoc comparisons following mixed-effects models will be adjusted using the multiple-comparison procedures provided by the statistical software.

Furthermore, in addressing feedback from an additional reviewer, we restructured the description of the statistical methods to more accurately represent the decision hierarchy and clarified our strategy for managing multiple comparisons. These modifications enhance the clarity of the statistical analysis plan while maintaining the integrity of the underlying methodology.

Reviewer #2: Christopher de Souza - The authors need to include exclusion criteria ear disease, a history of ear surgery. This would explain the cause of the tinnitus. The authors also need to determine if it is single sided tinnitus or bilateral tinnitus. Other criteria such as conditions like hypertension, diabetes etc need to be clearly excluded as a possible cause of tinnitus. They also need to specify if the patients were also offered medications. Subjective Tinnitus is notoriously difficult to evaluate. While their approach / concept is novel I recommend that they revise their manuscript to attend to the deficiencies that are present. Otological examination and evaluation is an absolute must. Once these are attended to I suggest the authors resubmit their very interesting idea.

We thank the reviewer for this helpful comment.

Although tinnitus is thought to result from abnormal neuroplastic changes in the central nervous system, its precise cause remains unclear (Zhang et al., 2020). Identifying neural correlates is challenging due to variability among tinnitus patients and controls (Elgoyhen et al., 2015). Differences such as hearing loss, hyperacusis, hypertension, or depression complicate efforts to pinpoint tinnitus-specific effects. Uncontrolled variables may explain inconsistent results in imaging studies linking tinnitus to neural abnormalities (Vanneste & De Ridder, 2015 ; Vanneste et

al., 2010; Vanneste et al., 2011). Using within-subject designs where tinnitus can be toggled helps control variables and identify neural sources of tinnitus.

According to a recent clinical study (Chang-Dong Wang, 2023), patients with tinnitus associated with Meniere's disease, pulsatile tinnitus, central nervous system disorders, otosclerosis, or a history of prior middle ear surgery were excluded from participation.

Our clinical research investigates how individual and group RAPP training influences psychological, physiological, and neuromodulatory outcomes. Therefore, we do not exclude patients with organic, metabolic, or psychological disorders, except for those experiencing auditory hallucinations, as these may impact EEG-HD data.

Patients are excluded from participation if they have uncorrected bilateral hearing loss greater than 40dB, possess a Current White Noise Generator Port, have received prescriptions for surgery, medications, or mind-body treatments within two months prior to enrolment, have previously participated in sound mediation group sessions, have consulted with a phoniatric specialist more than twice, or have attended more than two group workshops involving sound mediation.

All types of patients with subjective tinnitus lasting at least 3 months and THI above 38 at T0 are included, except for pulsatile tinnitus.

During medical recruitment consultation, audiometry with acouphenometry assesses the side, intensity, and frequency of tinnitus, residual inhibition, and somatosensory factors like SADAM, SROS, bruxism, and cervical disorders. The EVA discomfort test tracks changes in tinnitus-related discomfort and five other criteria (hyperacusis, jaw issues, cervical problems, sleep, anxiety) throughout the protocol, highlighting symptoms that may influence RAPP training outcomes.

We explain this in the section Inclusion and exclusion criteria

Reviewer #3: Its a well-structured, innovative study protocol with clear clinical relevance, however several areas need strengthening.

1. Clarity of intervention (APPR) needs standardization, APPR is described conceptually but not operationally standardized. Exact exercise protocol (steps, duration per component, Dose consistency across participants, Fidelity monitoring.

Thank you for this comment. We acknowledge that the lack of a detailed description of the APPR training prevents the reproducibility of the protocol.

The procedures and timeline for the 5-week training are consistent across all participants in both the test and control groups. Comprehensive details regarding these steps have been provided in Section 5.2.4, which also includes a translated weekly schedule for the RAPP training program.

After the clinical study, our six daily training videos will be open source to standardize RAPP exercises in clear progressive steps:

- Proprioception exercises 1a and 1b,
- Sound exercises 2a and 2b,
- Residual inhibition exercises 3a and 3b.

Despite careful planning, standardising five consecutive weekly group sound mediation workshops is challenging. Variations in group dynamics, stemming from the therapists' and

patients' personalities, attitudes, and engagement, significantly affect each session, regardless of hearing impairment.

Fidelity to individual and group training is tracked after completion through two methods: patients submit a follow-up sheet during their consultation, and all protocol participants complete an online questionnaire detailing the number of views, exercises practiced from six training videos, experiences during five group sessions, and key takeaways from each session video.

We explain this in the section 5-2-4 RAPP Training

2. Control group design – Potential bias, delayed intervention control is acceptable, but: no active comparator (e.g., standard TRT or sound therapy), Risk of placebo / attention bias.

Thank you for this comment. We recognize that not using an active comparator could lead to non-specific effects like expectancy and attention bias. We will tackle this limitation in our upcoming analysis.

The selection of the delayed-intervention control group in this study was based on ethical considerations and practical feasibility. The primary objective is not to demonstrate that RAPP training is superior to other therapies, such as standard TRT or sound therapy, but rather to establish its effectiveness and potential as an alternative, particularly in cases where hearing loss is absent or limited to mild deafness that does not necessitate hearing aids.

We completed the methodology section (5.1) of the protocol in response to reviewer 1, to clarify the design.

3. Primary endpoint: THI at T1, Hypothesis (12-point difference) is stated but missing: clear minimal clinically important difference (MCID) justification, clear hypothesis comparison wording.

We thank the reviewer for this comment.

We have revised the sample size justification to explicitly state the published minimal clinically important difference (MCID) for the THI (7 points according to Zeeman et al., 2011) and to explain the rationale for selecting an expected between-group difference of 12 points. As no previous study has evaluated the effect of RAPP training on the THI, this effect size was considered clinically meaningful because it exceeds the established MCID while remaining more conservative than the treatment effects reported in previous randomized trials of other non-invasive tinnitus interventions.

We also clarified the wording of the primary hypothesis by explicitly stating that participants in the RAPP group are expected to demonstrate a 12-point greater reduction in THI score between T0 and T1 than participants with no training.

4. Statistical analysis needs clarity, overly verbose and repetitive, multiple tests listed without decision hierarchy.

We thank the reviewer for this comment.

The Statistical Analysis section has been revised to improve clarity and reduce unnecessary repetition while preserving the pre-specified statistical analysis plan. We have explicitly identified the primary THI analysis as the only confirmatory hypothesis test and clarified that all secondary outcome analyses are exploratory.

We also reorganized the description of the statistical methods to better reflect the decision hierarchy and clarified the approach for handling multiple comparisons among secondary outcomes. These revisions improve the readability of the statistical analysis plan without modifying the statistical methodology.

5. HD-EEG analysis is under-specified it is missing, exact pipelines, software/tools, primary EEG outcome.

We thank the reviewer for this comment and agree that the HD-EEG analysis section was under-specified in the previous version, particularly regarding the processing pipeline, software tools, and definition of the main EEG outcome.

We have therefore revised the Methods section to provide a more reproducible and transparent HD-EEG analysis plan.

6. Check from grammatical errors.

We have made grammatical corrections, especially in section 8.2.

7. Too many keywords restrict to 5-6 core terms.

We have reduced to 4 keywords:

- Subjective tinnitus,
- neuromodulation,
- HD EEG,
- audio-proprio-phonatory reinforcement

---

7. PLOS authors have the option to publish the peer review history of their article (what does this mean?). If published, this will include your full peer review and any attached files.

If you choose “no”, your identity will remain anonymous but your review may still be made public.

Do you want your identity to be public for this peer review? For information about this choice, including consent withdrawal, please see our Privacy Policy.

Reviewer #1: No

Reviewer #2: **Yes:** Christopher de Souza

Reviewer #3: No
