# Supplementary material for: Neuromodulatory effects of audio-proprio-phonatory reinforcement training on subjective tinnitus, a HD-EEG study protocol
Source: PLoS One. 2026 Nov 6;21(11):e0357455. doi: 10.1371/journal.pone.0357455 (PMC13742906; doi:10.1371/journal.pone.0357455)
Supplement: Peer review history

# Author comment: Author response to Decision Letter 0

**DOI:** 10.1371/journal.pone.0357455.r001

**Transfer Alert**

This paper was transferred from another journal. As a result, its full editorial history (including decision letters, peer reviews and author responses) may not be present.

20 Mar 2026

# Peer review: Decision Letter 0

**DOI:** 10.1371/journal.pone.0357455.r002

**Authors:** Gauri Mankekar

3 Jun 2026

Dear Dr. Marié-Bailly,

Thank you for submitting your manuscript to PLOS ONE. After careful consideration, we feel that it has merit but does not fully meet PLOS ONE’s publication criteria as it currently stands. Therefore, we invite you to submit a revised version of the manuscript that addresses the points raised during the review process.

Please submit your revised manuscript by Jul 18 2026 11:59PM. If you will need more time than this to complete your revisions, please reply to this message or contact the journal office at plosone@plos.org. When you're ready to submit your revision, log on to https://www.editorialmanager.com/pone/ and select the 'Submissions Needing Revision' folder to locate your manuscript file.

- A letter that responds to each point raised by the academic editor and reviewer(s). You should upload this letter as a separate file labeled 'Response to Reviewers'.
- A marked-up copy of your manuscript that highlights changes made to the original version. You should upload this as a separate file labeled 'Revised Manuscript with Track Changes'.
- An unmarked version of your revised paper without tracked changes. You should upload this as a separate file labeled 'Manuscript'.

If you would like to make changes to your financial disclosure, please include your updated statement in your cover letter. Guidelines for resubmitting your figure files are available below the reviewer comments at the end of this letter.

If applicable, we recommend that you deposit your laboratory protocols in protocols.io to enhance the reproducibility of your results. Protocols.io assigns your protocol its own identifier (DOI) so that it can be cited independently in the future. For instructions see: https://journals.plos.org/plosone/s/submission-guidelines#loc-laboratory-protocols. Additionally, PLOS ONE offers an option for publishing peer-reviewed Lab Protocol articles, which describe protocols hosted on protocols.io. Read more information on sharing protocols at https://plos.org/protocols?utm_medium=editorial-email&utm_source=authorletters&utm_campaign=protocols.

As the corresponding author, your ORCID iD is verified in the submission system and will appear in the published article. PLOS supports the use of ORCID, and we encourage all coauthors to register for an ORCID iD and use it as well. Please encourage your coauthors to verify their ORCID iD within the submission system before final acceptance, as unverified ORCID iDs will not appear in the published article. *Only*  the individual author can complete the verification step; PLOS staff *cannot*  verify ORCID iDs on behalf of authors.

We look forward to receiving your revised manuscript.

Kind regards,

Gauri Mankekar, MD,PhD,FACS

Academic Editor

PLOS One

Journal Requirements:

When submitting your revision, we need you to address these additional requirements.

1.Please ensure that your manuscript meets PLOS ONE's style requirements, including those for file naming. The PLOS ONE style templates can be found at

https://journals.plos.org/plosone/s/file?id=wjVg/PLOSOne_formatting_sample_main_body.pdf and

https://journals.plos.org/plosone/s/file?id=ba62/PLOSOne_formatting_sample_title_authors_affiliations.pdf

2. Thank you for stating the following in the Competing Interests section:

“The authors have declared that no competing interests exist.”

We note that one or more of the authors are employed by a commercial company: SIOPI President & Cofounder Grenoble Auvergne-Rhône-Alpes France and MINDIG Company.

1.        Please provide an amended Funding Statement declaring this commercial affiliation, as well as a statement regarding the Role of Funders in your study. If the funding organization did not play a role in the study design, data collection and analysis, decision to publish, or preparation of the manuscript and only provided financial support in the form of authors' salaries and/or research materials, please review your statements relating to the author contributions, and ensure you have specifically and accurately indicated the role(s) that these authors had in your study. You can update author roles in the Author Contributions section of the online submission form.

Please also include the following statement within your amended Funding Statement.

“The funder provided support in the form of salaries for authors [insert relevant initials], but did not have any additional role in the study design, data collection and analysis, decision to publish, or preparation of the manuscript. The specific roles of these authors are articulated in the ‘author contributions’ section.”

If your commercial affiliation did play a role in your study, please state and explain this role within your updated Funding Statement.

2. Please also provide an updated Competing Interests Statement declaring this commercial affiliation along with any other relevant declarations relating to employment, consultancy, patents, products in development, or marketed products, etc.

Within your Competing Interests Statement, please confirm that this commercial affiliation does not alter your adherence to all PLOS ONE policies on sharing data and materials by including the following statement: "This does not alter our adherence to  PLOS ONE policies on sharing data and materials.” (as detailed online in our guide for authors http://journals.plos.org/plosone/s/competing-interests) . If this adherence statement is not accurate and  there are restrictions on sharing of data and/or materials, please state these. Please note that we cannot proceed with consideration of your article until this information has been declared.

Please include both an updated Funding Statement and Competing Interests Statement in your cover letter. We will change the online submission form on your behalf.

3. Your ethics statement should only appear in the Methods section of your manuscript. If your ethics statement is written in any section besides the Methods, please move it to the Methods section and delete it from any other section. Please ensure that your ethics statement is included in your manuscript, as the ethics statement entered into the online submission form will not be published alongside your manuscript.

4. We note you have included a table to which you do not refer in the text of your manuscript. Please ensure that you refer to Table 1 and 2 in your text; if accepted, production will need this reference to link the reader to the Table.

5. We note that the original protocol file you uploaded contains a confidentiality notice indicating that the protocol may not be shared publicly or be published. Please note, however, that the PLOS Editorial Policy requires that the original protocol be published alongside your manuscript in the event of acceptance. Please note that should your paper be accepted, all content including the protocol will be published under the Creative Commons Attribution (CC BY) 4.0 license, which means that it will be freely available online, and any third party is permitted to access, download, copy, distribute, and use these materials in any way, even commercially, with proper attribution.

Therefore, we ask that you please seek permission from the study sponsor or body imposing the restriction on sharing this document to publish this protocol under CC BY 4.0 if your work is accepted. We kindly ask that you upload a formal statement signed by an institutional representative clarifying whether you will be able to comply with this policy. Additionally, please upload a clean copy of the protocol with the confidentiality notice (and any copyrighted institutional logos or signatures) removed.

6. We note that the original protocol that you have uploaded as a Supporting Information file contains an institutional logo. As this logo is likely copyrighted, we ask that you please remove it from this file and upload an updated version upon resubmission.

7. If the reviewer comments include a recommendation to cite specific previously published works, please review and evaluate these publications to determine whether they are relevant and should be cited. There is no requirement to cite these works unless the editor has indicated otherwise.

Additional Editor Comments:

Please see the reviewer's comments. Please respond to their comments, revise and resubmit your manuscript.

[Note: HTML markup is below. Please do not edit.]

Reviewers' comments:

Reviewer's Responses to Questions

**Comments to the Author**

1. Does the manuscript provide a valid rationale for the proposed study, with clearly identified and justified research questions?

Reviewer #1: Yes

Reviewer #2: Yes

Reviewer #3: Yes

**********

2. Is the protocol technically sound and planned in a manner that will lead to a meaningful outcome and allow testing the stated hypotheses?

Reviewer #1: Partly

Reviewer #2: Partly

Reviewer #3: Yes

**********

3. Is the methodology feasible and described in sufficient detail to allow the work to be replicable?

Reviewer #1: No

Reviewer #2: Yes

Reviewer #3: Yes

**********

4. Have the authors described where all data underlying the findings will be made available when the study is complete?

The PLOS Data policy

Reviewer #1: Yes

Reviewer #2: Yes

Reviewer #3: Yes

**********

5. Is the manuscript presented in an intelligible fashion and written in standard English?

Reviewer #1: Yes

Reviewer #2: Yes

Reviewer #3: Yes

**********

Please use the space provided to explain your answers to the questions above and, if applicable, provide comments about issues authors must address before this protocol can be accepted for publication. You may also include additional comments for the author, including concerns about research or publication ethics.

You may also provide optional suggestions and comments to authors that they might find helpful in planning their study.

Reviewer #1: The protocol elements have been thought out rather well. However, from the statistical perspective, there are clarifications and additions needed. Specifically:

1. The authors state that this is a prospective, controlled, block-randomized trial involving two parallel groups (1:1 ratio) of patients. According to Figure 1 it looks like a crossover design. Presumably the intent is the same. Please clarify.

2. The recruitment procedure looks fine. What about a retention strategy to minimize missing data.

3. The sample size calculation looks reasonable. However, the investigators note that they will compare the change in THI score from T0 to T1 in group A, which receives APPR training, with the change in THI score from T0 to T1 in group B, which does not receive training. According to Figure 1 and Table 1 Group B receives training later. Again, is this merely a crossover design?

4. The Data management section needs a quality control component to assure consistency of data collection and recording.

5. The Data analysis section looks reasonable with the univariate comparisons for change at T1. However, there are multiple tests being done. They are:

A. VAS-A tinnitus scales will be compared between the two groups (from T0 to T1) using the same statistical tests (Student's t-test, or Student's test, or Wilcoxon-Mann-Whitney test, as needed.

B. The VAS scales related to the five associated questions (noise, neck, jaw, sleep, stress-anxiety) will be compared between the two groups (from T0 to T1)

C. The stability over time of the VAS-A tinnitus scales and the THI score will be examined in group A, between T1 and T2 (post-training period).

One has to assure a proper post-hoc test for multiple comparisons adjustment for each A,B,C section testing. The mixed model procedure should accommodate the post hoc adjustment for part C. A Statistician can help with this. For parts A and B, a multiple testing caution should be noted since analysis within each of the multiple scales represents a need for p-value adjustment.

Reviewer #2: The authors need to include exclusion criteria ear disease, a history of ear surgery. This would explain the cause of the tinnitus. The authors also need to determine if it is single sided tinnitus or bilateral tinnitus. Other criteria such as conditions like hypertension, diabetes etc need to be clearly excluded as a possible cause of tinnitus. They also need to specify if the patients were also offered medications.Subjective Tinnitus is notoriously difficult to evaluate . While their approach / concept is novel I recommend that they revise their manuscript to attend to the deficiencies that are present. Otological examination and evaluation is an absolute must.Once these are attended to I suggest the authors resubmit their very interesting idea.

Reviewer #3: Its a well-structured, innovative study protocol with clear clinical relevance, however several areas need strengthening.

1. Clarity of intervention (APPR) needs standardization, APPR is described conceptually but not operationally standardized. Exact exercise protocol (steps, duration per component, Dose consistency across participants, Fidelity monitoring.

2. Control group design – Potential bias, delayed intervention control is acceptable, but: no active comparator (e.g., standard TRT or sound therapy), Risk of placebo / attention bias.

3. Primary endpoint: THI at T1, Hypothesis (12-point difference) is stated but missing: clear minimal clinically important difference (MCID) justification, clear hypothesis comparison wording.

4. Statistical analysis needs clarity, overly verbose and repetitive, multiple tests listed without decision hierarchy.

5. HD-EEG analysis is under-specified it is missing, exact pipelines, software/tools, primary EEG outcome.

6. Check from grammatical errors.

7. Two many keywords restrict to 5-6 core terms.

**********

what does this mean?). If published, this will include your full peer review and any attached files.

If you choose “no”, your identity will remain anonymous but your review may still be made public.

**Do you want your identity to be public for this peer review?**  For information about this choice, including consent withdrawal, please see our Privacy Policy

Reviewer #1: No

Reviewer #2: **Yes:**  Christopher de Souza

Reviewer #3: No

**********

[NOTE: If reviewer comments were submitted as an attachment file, they will be attached to this email and accessible via the submission site. Please log into your account, locate the manuscript record, and check for the action link "View Attachments". If this link does not appear, there are no attachment files.]

To ensure your figures meet our technical requirements, please review our figure guidelines: https://journals.plos.org/plosone/s/figures

You may also use PLOS’s free figure tool, NAAS, to help you prepare publication quality figures: https://journals.plos.org/plosone/s/figures#loc-tools-for-figure-preparation.

NAAS will assess whether your figures meet our technical requirements by comparing each figure against our figure specifications.

# Author comment: Author response to Decision Letter 1

**DOI:** 10.1371/journal.pone.0357455.r003

12 Aug 2026

RESPONS TO EDITOR COMMENTS

We have submitted in "Other" :

• A formal statement signed by the institutional representative confirming compliance with PLOS ONE policy, labeled “PHONACOU_Sponsor authorization_20260710”.

• A clean copy of the English protocol, with the confidentiality notice and institution’s logo removed, labeled “Revised Protocol with Track Changes”. This will be published as supporting information alongside the manuscript.

All authors were fully involved in the preparation of the manuscript. We confirm that neither the manuscript nor any parts of its contents are currently under consideration for publication with or published in another journal. All authors have approved the manuscript and agree with its submission to PLOS ONE. There is no commercial relationship which may lead to a conflict of interest. All authors have had full access to the data, they have the right to publish all the data, and they have had the right to obtain independent statistical analyses of the data.

We declare that no competing interests exist. These both commercial affiliations do not alter our adherence to all PLOS ONE policies on sharing data and materials:

• Robin Guillard – ORCID: 0000-0002-7557-5066: As president of Siopi, he contributed to setting up the digital tools on Siopi allowing the collection and secure storage of the questionnaire data necessary for the proper conduct of the study. We can mention that we have concluded a service provision contract between the Orléans University Hospital and Siopi.

• Mahmoud Hassan - ORCID: 0000-0003-0307-5086: MINDIG is involved in the planned HD-EEG data processing and analysis using pseudonymised EEG data. The company is involved in patient recruitment, clinical assessment, delivery of the intervention, or definition of the primary clinical endpoint. The primary outcome of the study is the change in Tinnitus Handicap Inventory score. The decision to submit and

RESPONS TO REVIEWERS

Reviewer #1: The protocol elements have been thought out rather well. However, from the statistical perspective, there are clarifications and additions needed. Specifically:

1. The authors state that this is a prospective, controlled, block-randomized trial involving two parallel groups (1:1 ratio) of patients. According to Figure 1 it looks like a crossover design. Presumably the intent is the same. Please clarify.

Thank you for this comment. The study is indeed a randomized two-sequence crossover trial. The Figure 1 is correct. The method section (5.1) has been revised accordingly to specify that participants are randomised (1:1) to one of two treatment sequences.

Using a parallel-group design would have meant that one group was excluded from receiving the experimental intervention, which raised ethical concerns. By implementing a crossover design, every participant was given the opportunity to access the intervention, and researchers could still compare outcomes between the RAPP training period and the period without training.

This has been clarified in the Methods section (5.1) of the revised protocol.

2. The recruitment procedure looks fine. What about a retention strategy to minimize missing data.

Thank you for this comment. Regarding the strategy for handling missing data, participants will receive verbal reminders during study visits and interactions only. More intensive reminders were deliberately avoided, as repeatedly prompting participants to assess their tinnitus may increase their focus on it and interfere with the habituation process (which is a key objective of the intervention).

3. The sample size calculation looks reasonable. However, the investigators note that they will compare the change in THI score from T0 to T1 in group A, which receives APPR training, with the change in THI score from T0 to T1 in group B, which does not receive training. According to Figure 1 and Table 1 Group B receives training later. Again, is this merely a crossover design?

Thank you for your comment. You are right; this study uses a two-sequence crossover design. We selected the crossover approach mainly for ethical reasons, so that every participant would have the opportunity to receive the experimental intervention.

We do not currently have a clear hypothesis regarding the duration or persistence of the intervention effects over time, and this aspect is itself one of the exploratory objectives of the study. Therefore, a residual or carry-over effect between periods cannot be excluded.

For this reason, the comparison of THI score after APPR training versus no training is intentionally restricted to the first study period only (T0 to T1).

This has been clarified in the Methods section (5.1) of the revised protocol.

4. The Data management section needs a quality control component to assure consistency of data collection and recording.

We thank the reviewer for this pertinent observation. We have completed the "Data Management" section by including a section dedicated to quality control. This specifies the procedures put in place to ensure consistency in data collection and recording, including systematic verification of data at input, the use of standardized formats and predefined value ranges, as well as regular audits of the data collected to detect any anomalies or inconsistencies. These measures are in line with the best practices in force in the field and aim to guarantee the reproducibility and reliability of the analyses.

We hope that these clarifications meet the reviewer's request.

5. The Data analysis section looks reasonable with the univariate comparisons for change at T1. However, there are multiple tests being done. They are:

A. VAS-A tinnitus scales will be compared between the two groups (from T0 to T1) using the same statistical tests (Student's t-test, or Student's test, or Wilcoxon-Mann-Whitney test, as needed.

B. The VAS scales related to the five associated questions (noise, neck, jaw, sleep, stress-anxiety) will be compared between the two groups (from T0 to T1)

C. The stability over time of the VAS-A tinnitus scales and the THI score will be examined in group A, between T1 and T2 (post-training period).

One has to assure a proper post-hoc test for multiple comparisons adjustment for each A, B, C section testing. The mixed model procedure should accommodate the post hoc adjustment for part C. A Statistician can help with this. For parts A and B, a multiple testing caution should be noted since analysis within each of the multiple scales represents a need for p-value adjustment.

We thank the reviewer for this helpful comment.

We acknowledge the need to clarify our approach to multiple comparisons. The Statistical Analysis section now specifies that the primary THI analysis is designated as the sole confirmatory hypothesis test, while secondary outcome analyses are categorized as exploratory. Furthermore, we have indicated that appropriate adjustments for multiple testing, such as the Holm–Bonferroni correction, will be applied whenever multiple pairwise comparisons occur within the same family of secondary outcomes (e.g., VAS scales). Similarly, post hoc comparisons following mixed-effects models will be adjusted using the multiple-comparison procedures provided by the statistical software.

Furthermore, in addressing feedback from an additional reviewer, we restructured the description of the statistical methods to more accurately represent the decision hierarchy and clarified our strategy for managing multiple comparisons. These modifications enhance the clarity of the statistical analysis plan while maintaining the integrity of the underlying methodology.

Reviewer #2: Christopher de Souza - The authors need to include exclusion criteria ear disease, a history of ear surgery. This would explain the cause of the tinnitus. The authors also need to determine if it is single sided tinnitus or bilateral tinnitus. Other criteria such as conditions like hypertension, diabetes etc need to be clearly excluded as a possible cause of tinnitus. They also need to specify if the patients were also offered medications. Subjective Tinnitus is notoriously difficult to evaluate. While their approach / concept is novel I recommend that they revise their manuscript to attend to the deficiencies that are present. Otological examination and evaluation is an absolute must. Once these are attended to I suggest the authors resubmit their very interesting idea.

We thank the reviewer for this helpful comment.

Although tinnitus is thought to result from abnormal neuroplastic changes in the central nervous system, its precise cause remains unclear (Zhang et al., 2020). Identifying neural correlates is challenging due to variability among tinnitus patients and controls (Elgoyhen et al., 2015). Differences such as hearing loss, hyperacusis, hypertension, or depression complicate efforts to pinpoint tinnitus-specific effects. Uncontrolled variables may explain inconsistent results in imaging studies linking tinnitus to neural abnormalities (Vanneste & De Ridder, 2015 ; Vanneste et al., 2010; Vanneste et al., 2011). Using within-subject designs where tinnitus can be toggled helps control variables and identify neural sources of tinnitus.

According to a recent clinical study (Chang-Dong Wang, 2023), patients with tinnitus associated with Meniere’s disease, pulsatile tinnitus, central nervous system disorders, otosclerosis, or a history of prior middle ear surgery were excluded from participation.

Our clinical research investigates how individual and group RAPP training influences psychological, physiological, and neuromodulatory outcomes. Therefore, we do not exclude patients with organic, metabolic, or psychological disorders, except for those experiencing auditory hallucinations, as these may impact EEG-HD data.

Patients are excluded from participation if they have uncorrected bilateral hearing loss greater than 40dB, possess a Current White Noise Generator Port, have received prescriptions for surgery, medications, or mind-body treatments within two months prior to enrolment, have previously participated in sound mediation group sessions, have consulted with a phoniatric specialist more than twice, or have attended more than two group workshops involving sound mediation.

All types of patients with subjective tinnitus lasting at least 3 months and THI above 38 at T0 are included, except for pulsatile tinnitus.

During medical recruitment consultation, audiometry with acouphenometry assesses the side, intensity, and frequency of tinnitus, residual inhibition, and somatosensory factors like SADAM, SROS, bruxism, and cervical disorders. The EVA discomfort test tracks changes in tinnitus-related discomfort and five other criteria (hyperacusis, jaw issues, cervical problems, sleep, anxiety) throughout the protocol, highlighting symptoms that may influence RAPP training outcomes.

We explain this in the section Inclusion and exclusion criteria

Reviewer #3: Its a well-structured, innovative study protocol with clear clinical relevance, however several areas need strengthening.

1. Clarity of intervention (APPR) needs standardization, APPR is described conceptually but not operationally standardized. Exact exercise protocol (steps, duration per component, Dose consistency across participants, Fidelity monitoring.

Thank you for this comment. We acknowledge that the lack of a detailed description of the APPR training prevents the reproducibility of the protocol.

The procedures and timeline for the 5-week training are consistent across all participants in both the test and control groups. Comprehensive details regarding these steps have been provided in Section 5.2.4, which also includes a translated weekly schedule for the RAPP training program.

After the clinical study, our six daily training videos will be open source to standardize RAPP exercises in clear progressive steps:

• Proprioception exercises 1a and 1b,

• Sound exercises 2a and 2b,

• Residual inhibition exercises 3a and 3b.

Despite careful planning, standardising five consecutive weekly group sound mediation workshops is challenging. Variations in group dynamics, stemming from the therapists' and patients' personalities, attitudes, and engagement, significantly affect each session, regardless of hearing impairment.

Fidelity to individual and group training is tracked after completion through two methods: patients submit a follow-up sheet during their consultation, and all protocol participants complete an online questionnaire detailing the number of views, exercises practiced from six training videos, experiences during five group sessions, and key takeaways from each session video.

We explain this in the section 5-2-4 RAPP Training

2. Control group design – Potential bias, delayed intervention control is acceptable, but: no active comparator (e.g., standard TRT or sound therapy), Risk of placebo / attention bias.

Thank you for this comment. We recognize that not using an active comparator could lead to non-specific effects like expectancy and attention bias. We will tackle this limitation in our upcoming analysis.

The selection of the delayed-intervention control group in this study was based on ethical considerations and practical feasibility. The primary objective is not to demonstrate that RAPP training is superior to other therapies, such as standard TRT or sound therapy, but rather to establish its effectiveness and potential as an alternative, particularly in cases where hearing loss is absent or limited to mild deafness that does not necessitate hearing aids.

We completed the methodology section (5.1) of the protocol in response to reviewer 1, to clarify the design.

3. Primary endpoint: THI at T1, Hypothesis (12-point difference) is stated but missing: clear minimal clinically important difference (MCID) justification, clear hypothesis comparison wording.

We thank the reviewer for this comment.

We have revised the sample size justification to explicitly state the published minimal clinically important difference (MCID) for the THI (7 points according to Zeeman et al., 2011) and to explain the rationale for selecting an expected between-group difference of 12 points. As no previous study has evaluated the effect of RAPP training on the THI, this effect size was considered clinically meaningful because it exceeds the established MCID while remaining more conservative than the treatment effects reported in previous randomized trials of other non-invasive tinnitus interventions.

We also clarified the wording of the primary hypothesis by explicitly stating that participants in the RAPP group are expected to demonstrate a 12-point greater reduction in THI score between T0 and T1 than participants with no training.

4. Statistical analysis needs clarity, overly verbose and repetitive, multiple tests listed without decision hierarchy.

We thank the reviewer for this comment.

The Statistical Analysis section has been revised to improve clarity and reduce unnecessary repetition while preserving the pre-specified statistical analysis plan. We have explicitly identified the primary THI analysis as the only confirmatory hypothesis test and clarified that all secondary outcome analyses are exploratory.

We also reorganized the description of the statistical methods to better reflect the decision hierarchy and clarified the approach for handling multiple comparisons among secondary outcomes. These revisions improve the readability of the statistical analysis plan without modifying the statistical methodology.

5. HD-EEG analysis is under-specified it is missing, exact pipelines, software/tools, primary EEG outcome.

We thank the reviewer for this comment and agree that the HD-EEG analysis section was under-specified in the previous version, particularly regarding the processing pipeline, software tools, and definition of the main EEG outcome.

We have therefore revised the Methods section to provide a more reproducible and transparent HD-EEG analysis plan.

6. Check from grammatical errors.

We have made grammatical corrections, especially in section 8.2.

7. Too many keywords restrict to 5-6 core terms.

We have reduced to 4 keywords:

- Subjective tinnitus,

- neuromodulation,

- HD EEG,

- audio-proprio-phonatory reinforcement

Attachment Submitted filename: Response to Reviewers.pdf

# Peer review: Decision Letter 1

**DOI:** 10.1371/journal.pone.0357455.r004

**Authors:** Gauri Mankekar

18 Aug 2026

Neuromodulatory effects of audio-proprio-phonatory reinforcement training on subjective tinnitus, a HD-EEG study protocol

PONE-D-26-07098R1

Dear Dr.Isabelle Marié-Bailly,

We’re pleased to inform you that your manuscript has been judged scientifically suitable for publication and will be formally accepted for publication once it meets all outstanding technical requirements.

Within one week, you’ll receive an e-mail detailing the required amendments. When these have been addressed, you’ll receive a formal acceptance letter and your manuscript will be scheduled for publication.

An invoice will be generated when your article is formally accepted. Please note, if your institution has a publishing partnership with PLOS and your article meets the relevant criteria, all or part of your publication costs will be covered. Please make sure your user information is up-to-date by logging into Editorial Manager at Editorial Manager® and clicking the ‘Update My Information' link at the top of the page. For questions related to billing, please contact billing support.

If your institution or institutions have a press office, please notify them about your upcoming paper to help maximize its impact. If they’ll be preparing press materials, please inform our press team as soon as possible -- no later than 48 hours after receiving the formal acceptance. Your manuscript will remain under strict press embargo until 2 pm Eastern Time on the date of publication. For more information, please contact onepress@plos.org.

Kind regards,

Gauri Mankekar, MD,PhD,FACS

Academic Editor

PLOS One

Additional Editor Comments (optional):

Reviewers' comments:

Reviewer's Responses to Questions

**Comments to the Author**

1. Does the manuscript provide a valid rationale for the proposed study, with clearly identified and justified research questions?

Reviewer #1: Yes

**********

2. Is the protocol technically sound and planned in a manner that will lead to a meaningful outcome and allow testing the stated hypotheses?

Reviewer #1: Yes

**********

3. Is the methodology feasible and described in sufficient detail to allow the work to be replicable?

Reviewer #1: Yes

**********

4. Have the authors described where all data underlying the findings will be made available when the study is complete?

The PLOS Data policy

Reviewer #1: Yes

**********

5. Is the manuscript presented in an intelligible fashion and written in standard English?

Reviewer #1: Yes

**********

Please use the space provided to explain your answers to the questions above and, if applicable, provide comments about issues authors must address before this protocol can be accepted for publication. You may also include additional comments for the author, including concerns about research or publication ethics.

You may also provide optional suggestions and comments to authors that they might find helpful in planning their study.

Reviewer #1: All comments have been addressed and the revisions noted.

XXXXXXXXXXXXXXXXXXXXXXXXXXXXXXXXXXXXXXXXXXXXXXXXXXXXXXXXXXXXXXXXXXXXXXXXXXXXXXXXXXXXXXXXXXXXXXXXXXXXXXXXXXXXXXXXXXXXXXXXXXXXXXXXXXXXXXXXXXXXXXXXXXXXXXXXXXXXXXXXXXXXXXXXXXXXXXXXXXXXXXXXXXXXXXXXXXX

**********

what does this mean?). If published, this will include your full peer review and any attached files.

If you choose “no”, your identity will remain anonymous but your review may still be made public.

**Do you want your identity to be public for this peer review?**  For information about this choice, including consent withdrawal, please see our Privacy Policy

Reviewer #1: No

**********

# Editor report: Acceptance letter

**DOI:** 10.1371/journal.pone.0357455.r005

**Authors:** Gauri Mankekar

PONE-D-26-07098R1

PLOS One

Dear Dr. Marié-Bailly,

I'm pleased to inform you that your manuscript has been deemed suitable for publication in PLOS One. Congratulations! Your manuscript is now being handed over to our production team.

At this stage, our production department will prepare your paper for publication. This includes ensuring the following:

* All references, tables, and figures are properly cited

* All relevant supporting information is included in the manuscript submission,

* There are no issues that prevent the paper from being properly typeset

You will receive further instructions from the production team, including instructions on how to review your proof when it is ready. Please keep in mind that we are working through a large volume of accepted articles, so please give us a few days to review your paper and let you know the next and final steps.

Lastly, if your institution or institutions have a press office, please let them know about your upcoming paper now to help maximize its impact. If they'll be preparing press materials, please inform our press team within the next 48 hours. Your manuscript will remain under strict press embargo until 2 pm Eastern Time on the date of publication. For more information, please contact onepress@plos.org.

You will receive an invoice from PLOS for your publication fee after your manuscript has reached the completed accept phase. If you receive an email requesting payment before acceptance or for any other service, this may be a phishing scheme. Learn how to identify phishing emails and protect your accounts at https://explore.plos.org/phishing.

If we can help with anything else, please email us at customercare@plos.org.

Thank you for submitting your work to PLOS One and supporting open access.

Kind regards,

PLOS One Editorial Office Staff

on behalf of

Dr. Gauri Mankekar

Academic Editor

PLOS One
